# Transcriptome and physiological analyses for revealing genes involved in wheat response to endoplasmic reticulum stress

**DOI:** 10.1186/s12870-019-1798-7

**Published:** 2019-05-09

**Authors:** Xing Yu, Tanchun Wang, Meichen Zhu, Liting Zhang, Fengzhi Zhang, Enen Jing, Yongzhe Ren, Zhiqiang Wang, Zeyu Xin, Tongbao Lin

**Affiliations:** 1 College of Agronomy, Henan Agricultural University, Zhengzhou, China; 2 Collaborative Innovation Center of Henan Grain Crops, Zhengzhou, China; 3 National Key Laboratory of Wheat and Maize Crop Science, Zhengzhou, China; 4 Department of Basic Biomedical Sciences, Touro College of Osteopathic Medicine – Middletown, NY, USA

**Keywords:** Antioxidant enzymes, DTT, TUDCA, RNA-seq, Transcription factors, Chlorophyll

## Abstract

**Background:**

Wheat production is largely restricted by adverse environmental stresses. Under many undesirable conditions, endoplasmic reticulum (ER) stress can be induced. However, the physiological and molecular responses of wheat to ER stress remain poorly understood. We used dithiothreitol (DTT) and tauroursodeoxycholic acid (TUDCA) to induce or suppress ER stress in wheat cells, respectively, with the aim to reveal the molecular background of ER stress responses using a combined approach of transcriptional profiling and morpho-physiological characterization.

**Methods:**

To understand the mechanism of wheat response to ER stress, three wheat cultivars were used in our pre-experiments. Among them, the cultivar with a moderate stress tolerance, Yunong211 was used in the following experiments. We used DTT (7.5 mM) to induce ER stress and TUDCA (25 μg·mL^− 1^) to suppress the stress. Under three treatment groups (Control, DTT and DTT + TUDCA), we firstly monitored the morphological, physiological and cytological changes of wheat seedlings. Then we collected leaf samples from each group for RNA extraction, library construction and RNA sequencing on an Illumina Hiseq platform. The sequencing data was then validated by qRT-PCR.

**Results:**

Morpho-physiological results showed DTT significantly reduced plant height and biomass, decreased contents of chlorophyll and water, increased electrolyte leakage rate and antioxidant enzymes activity, and accelerated the cell death ratio, whereas these changes were all remarkably alleviated after TUDCA co-treatment. Therefore, RNA sequencing was performed to determine the genes involved in regulating wheat response to stress. Transcriptomic analysis revealed that 8204 genes were differentially expressed in three treatment groups. Among these genes, 158 photosynthesis-related genes, 42 antioxidant enzyme genes, 318 plant hormone-related genes and 457 transcription factors (TFs) may play vital roles in regulating wheat response to ER stress. Based on the comprehensive analysis, we propose a hypothetical model to elucidate possible mechanisms of how plants adapt to environmental stresses.

**Conclusions:**

We identified several important genes that may play vital roles in wheat responding to ER stress. This work should lay the foundations of future studies in plant response to environmental stresses.

**Electronic supplementary material:**

The online version of this article (10.1186/s12870-019-1798-7) contains supplementary material, which is available to authorized users.

## Background

Wheat (*Triticum aestivum* L.) is one of the primary cereal plants in the world, globally providing a staple food for much of the human population. Because of the high level of resistance to most biotic and abiotic stresses, wheat is grown on more land area worldwide than any other grain crop. However, wheat production is largely restricted by adverse environmental conditions, including drought, heat, salt and pathogen infection. Under these conditions, endoplasmic reticulum (ER) stress can be induced [1–5]. To adapt to numerous stresses, plants evolved intricate mechanisms to help perceive the environmental signals and enable optimal responses. Among the mechanisms, ER stress signaling in plants plays a vital role as an adaptive mechanism and has been well studied in *Arabidopsis* [3, 6], tobacco [5], rice [7, 8] and maize [9] in recent years, while understanding of this pathway in crop plants remains limited. Therefore, research on wheat ER stress responses will be of great significance to improve environmental stress tolerance in crop plants.

The ER is a functional organelle for secreted proteins and membrane protein synthesis, folding, assembly, transportation and modification. The ER is also the primary reservoir for intracellular calcium ion storage. Additionally, the ER is highly sensitive to the effects of stress on intracellular energy levels, oxidative status, and calcium ion concentrations [10–12]. Therefore, a sophisticated ER quality control (ERQC) system guarantees unerring folding of proteins in cells [13, 14]. However, the harmonious state is often disturbed by environmental stresses. Consequently, the folding process is disturbed and compromised, and the demands for protein folding exceed the ER folding capacity, followed by the accumulation of misfolded and unfolded proteins in the ER lumen, leading to ER stress [6, 11]. When ER stress is mild or short-term, the cells can initiate the unfolded protein response (UPR) and regulate ER stress responsive genes encoding molecular chaperones and protein folding and protein degradation factors to help proteins fold properly or conduct ER-associated degradation (ERAD) [6, 14–16]. However, when ER stress is severe or chronic and cannot be released effectively, the stress can trigger cell death and disturb the growth of plants [6, 17, 18].

Molecular chaperones are proteins that increase the efficiency of the folding process [19]. The chaperones primarily include Bip, calnexin (CNX)/calreticulin (CRT), PDIs, GRP94, ER oxidoreductase 1 (Ero1), and DnaJ (Hsp40 family), among others. Bip, also known as glucose-regulated protein 78 (GRP78), which is a member of the Hsp70 family, is the most abundant chaperone protein in the ER lumen that prevents protein aggregation and assists in correctly folding proteins [3]. GRP94, in the Hsp90 family, similar to Bip, is also an abundant glucose-regulated chaperone protein in the ER lumen that functions in the processing and transport of secreted proteins [3]. Other chaperones, such as CNX/CRT, PDIs and DnaJ, also play important roles in ER protein folding. In mammals, inositol-requiring enzyme-1 (IRE1), protein kinase RNA (PKR)-like ER kinase (PERK), and activating transcription 6 (ATF6) are three different classes of membrane-associated sensor transducers, which are activated by a UPR [11, 17, 20]. According to studies in recent years, membrane-associated transcription factors (MTFs) of basic region/leucine zipper motif (bZIP) and NAC (NAM, ATAF, and CUC) families are involved in regulating ER stress signaling and play important roles in AtbZIP28 [21–23], IRE1-bZIP60 [4, 24, 25], ANAC103 [26], ANAC062 [27] and ANAC089 [28] pathways.

When plants are subjected to ER stress, reactive oxygen species (ROS) are induced, leading to oxidative damage, such as lipid peroxidation, protein oxidation and H_2_O_2_ accumulation [29]. ROS have also emerged as important signals in the activation of plant programmed cell death (PCD) [30–32]. Plants mediate ROS levels and oxidative damage through several mechanisms, including increasing the activity of antioxidant enzymes (e.g., SOD, POD and CAT) [29], accelerating the degradation of chloroplasts [33], and increasing the cytomembrane permeability and electrolyte leakage rate [10]. Furthermore, ER stress can stimulate the release of calcium from the ER to mitochondria, which can interfere with protein folding, and the increased levels of calcium ions in the mitochondria can lead to the release of cytochrome C (Cyt C), which promotes oxidative stress and finally leads to cell death [18, 32].

To facilitate ER stress-related research, in most cases, dithiothreitol (DTT) and tunicamycin (TM) are used as stress activators [10, 13, 34, 35]. DTT is a redox reagent that can destroy the oxidation conditions required for the formation of disulfide bonds, whereas TM can specifically block N-glycosylation by inhibiting key step information. On the other hand, the chemical chaperones tauroursodeoxycholic acid (TUDCA) and 4-phenylbutyrate (PBA) can alleviate ER stress by stabilizing unfolded proteins and preventing their aggregation [36] and reduce TM- or DTT-induced autophagy by preventing the accumulation of unfolded or misfolded proteins [35]. In addition, TUDCA and PBA can relieve ER stress-mediated cell death caused by TM [10]. Although well-studied cases on ER stress signaling have contributed outstanding advances, studies investigating this pathway in wheat remain limited. Therefore, to determine the mechanism of ER stress responses in wheat, we used DTT and TUDCA to induce or suppress ER stress, respectively. Morpho-physiological changes were examined under different treatments to confirm the effects of DTT and TUDCA, and we subsequently identified genes responsive to ER stress with RNA-seq.

## Methods

### Plant growth and treatments

Wheat cultivar Yunong211 was used in this study, which was cultivated by Henan Agricultural University, China through breeding Yunong201//Yunong9234903/Baiyingdong. In our previous studies, we found Yunong211 responded to both osmotic stress and ER stress with a moderate tolerance [37 and unpublished data].

Seeds were surface-sterilized, soaked in tap water for 24 h, and then germinated in a dark incubator at 25 °C for 3 days followed by 2 days under light condition. The plantlets with uniform sizes were transferred to containers filled with half Hoagland nutrient solution and grown in a growth chamber with a 12 h photoperiod (irradiance of 400 μmol·m^− 2^·s^− 1^), day/night temperature of 25 °C, and relative humidity of 60%.

Experiments were established with three treatment groups: the control (C, without any chemical treatment), DTT (D, treated with DTT only), and DTT + TUDCA (T, treated with DTT and TUDCA). According to the previous reports, we had standardized the dosages of DTT [35] and TUDCA [37] and the way of their application on wheat seedlings before we formally started this work. For DTT treatment, the nutrient medium was supplemented with 7.5 mM DTT (Additional file 1: Figure S1). For DTT + TUDCA co-treatment, 25 μg·mL^− 1^ TUDCA (Additional file 2: Figure S2) was added with 7.5 mM DTT. Wheat seedlings were sampled for measurements on different days, and three independent biological replicates were performed for each measurement.

To evaluate plant growth after treatments, seedling height and root length were measured with a centimeter scale daily throughout the entire experiment. To determine fresh weight (FW) and dry weight (DW), fresh plants were measured with a balance, subsequently oven-dried at 105 °C for 30 min, and then at 80 °C until constant weight was achieved. Water content was calculated using the following formula: Water content (%) = (FW - DW) / FW × 100.

### Evaluation of cell membrane permeability

Cell membrane permeability was represented by the electrolyte leakage rate, which was measured with a conductivity meter and calculated according to Bajji et al. [38]. Equal length of seedling leaf segments were placed into 10 mL of distilled water. Then shocked at room temperature for 24 h for estimating the initial electrical conductivity (R_1_), subsequently estimating the final electrical conductivity (R_2_) after boiling at 100 °C for 20 min and cooled to room temperature and also shocked at temperature for 24 h. R_0_ is the electrical conductivity of distilled water. So the electrolyte leakage rate was calculated as: Electrolyte leakage rate (%) = (R_1_ - R_0_) / (R_2_ - R_0_) × 100.

### Estimation of chlorophyll a and b contents

Chlorophyll a and b contents were measured with ethanol solvents according to Ritchie [39] with minor modifications. Fresh leaf sample (0.1 g) was soaked in 25 mL brown volumetric flask with 95% alcohol placed in the dark for 48 h at room temperature. Supernatant absorbance was measured at 665 nm and 649 nm wavelengths using the UV spectrophotometer. The chlorophyll a and b content was calculated as: chlorophyll a content (μg/g) = (13.95 × A665–6.88 × A649) × 25 / W; chlorophyll b content (μg/g) = (24.96 × A649–7.32 × A665) × 25 / W.

### Determination of antioxidant enzymes

Fresh wheat leaves (0.3 g) were put in 5 mL centrifuge tubes, grinding on a high-throughput tissue grinder. After grinding, homogenized with 3 mL 50 mM sodium phosphate buffer (pH 7.8) and centrifuged at 4000 rpm for 20 min at 4 °C. The supernatant fraction was designated as crude enzyme extract and stored at 4 °C for the assays of various antioxidant enzyme activity.

SOD (EC 1.15.1.1) activity was assayed following the method of Flohé and Otting [40] with some modifications. Briefly, SOD activity was performed by measurement of inhibition of photochemical reduction of nitroblue tetrazolium (NBT) at 560 nm. The assay mixture (3 mL) contained 50 mM sodium phosphate buffer (pH 7.8), 130 mM methionine, 100 μM EDTA-Na_2_, 20 μM riboflavin, 750 μM NBT, distilled water and enzyme extract (50 μL). The reaction was started by placing the tubes below two 15 W fluorescent lamps for 10 min and then stopped by switching off the light. One unit (U) of SOD activity is defined as the amount of enzyme required for 50% inhibition of NBT reduction under the experimental conditions.

CAT (EC 1.11.1.6) activity was measured according to the method of Chance and Maehly [41] with some modifications. CAT activity was assayed in a reaction mixture (3.1 mL) containing 150 mM sodium phosphate buffer (pH 7.0), 30% H_2_O_2_, and enzyme extract (100 μL). The reaction was started with the addition of the enzyme extract, and the CAT activity was assayed by monitoring the decrease in the absorbance at 240 nm as a consequence of H_2_O_2_ consumption. One unit (U) of CAT activity is defined as the OD value per minute is reduced by 0.01 under the experimental conditions.

### Detection of cell death

Trypan blue staining to detect cell death was conducted using a 0.04% trypan blue solution (Beijing Solarbio Science & Technology Co., Ltd., China) according to the procedure described by Desmond et al. [42]. For leaf staining, leaf segments were immersed and boiled in the diluted trypan blue solution (the proportion of 0.04% trypan blue solution and distilled water was 1:9) for 1 min and left to stain overnight before de-staining with chloral hydrate (1.25 g/mL) and viewed using a microscope (OLYMPUS BX-53; Japan). For root staining, root tissues were stained with diluted trypan blue solution for 1–3 min, rinsed in the tap water and recorded with a camera (Cannon EOS 70D; Japan) and microscope.

### RNA sequencing

Wheat leaves were collected after 2 days of treatment: control (C), DTT (D), and DTT + TUDCA (T). These samples were frozen immediately in liquid nitrogen and stored at − 80 °C until RNA extraction. Three independent biological replicates were performed, and a total of nine leaf samples were sent to a service company (Beijing Novogene Bioinformatics Technology Co. Ltd., China) for RNA extraction, library construction and RNA sequencing. Total RNA was extracted using TRNzol® Reagent (Tiangen Biotech, Beijing, China), according to the manufacturer’s instructions. RNA integrity was assessed using an Agilent 2100 Bioanalyzer (Agilent Technologies, CA, USA). The 9 libraries were sequenced on an Illumina Hiseq platform using 150 bp paired-end sequencing, generating an average of 68 million raw reads for each sample (Additional file 3: Table S1).

### Alignment of RNA sequencing reads and gene expression analysis

Clean data were obtained removing reads containing adapters, reads containing poly-N and low quality reads from raw data. The high-quality paired-end reads from each library were mapped to wheat reference genome (http://plants.ensembl.org/Triticum_aestivum) using TopHat v2.0.12. The transcripts were calculated and normalized to FPKM (fragments per kilobase of transcript sequence per million mapped reads), representing the gene expression levels [43]. DESeq software was used to identify differentially expressed genes (DEGs) in pair-wise comparisons. Genes with a *P*-value < 0.01 and an absolute value of log_2_ fold-change ≥1 found by DESeq were assigned as differentially expressed.

### GO and KEGG enrichment analysis

Gene Ontology (GO) enrichment analysis of DEGs was implemented by the GOseq R package in which gene length bias was corrected (http://www.geneontology.org/) [44]. GO terms with a corrected *P*-value < 0.05 were considered significantly enriched by DEGs. The *P*-values were adjusted using the Benjamini and Hochberg method [45]. KEGG (Kyoto Encyclopedia of Genes and Genomes) is a database used to understand the high-level functions and utilities of the biological system (http://www.genome.jp/kegg/) [46]. We mapped sequences to the reference authoritative pathways in KEGG to determine the active biological pathways in annotated unigene sequences, and *Oryza sativa japonica* (Japanese rice) was used as a reference species.

### Transcription factor analysis

Plant transcription factor (TFs) were predicted using iTAK software, which has the basic principle to identify TFs by hmmscan using well-defined families of TFs and rules in the database. The identification and classification of TFs are based on Pérez-Rodríguez et al. [47] and Jin et al. [48].

### Quantitative PCR

Quantitative real-time PCR (qRT-PCR) analysis was conducted using a Thermal Cycler CFX96 Real-Time System (BIO-RAD, USA). Each PCR reaction contained 2 μL of the diluted cDNA, 10 μL GoTaq® qPCR Master Mix (Promega, USA), 7.2 μL of nuclease-free water, and 0.8 μL of the forward and reverse primers in a 20 μL reaction mixture. The PCR cycling conditions were as follows: 95 °C for 2 min, followed by 40 cycles of 95 °C for 15 s, 60 °C for 30 s and 72 °C for 30 s. Three technical replicates and three independent biological replicates for each PCR reaction were performed for each gene. The *β-actin* gene was used for normalization of qRT-PCR data. The fold changes were calculated using the 2^-ΔΔCt^ method [49]. All primers for qRT-PCR are available in Additional file 4: Table S2.

### Statistical analyses

Morpho-physiological data were subjected to one-way ANOVA using the SPSS statistical software package 17.0 (SPSS Inc., Chicago, IL, USA). Duncan’s test was applied to assess the significant differences (*P*-value < 0.05) between treatments.

## Results

### Morphological and physiological changes under different treatments

**Fig. 1:**
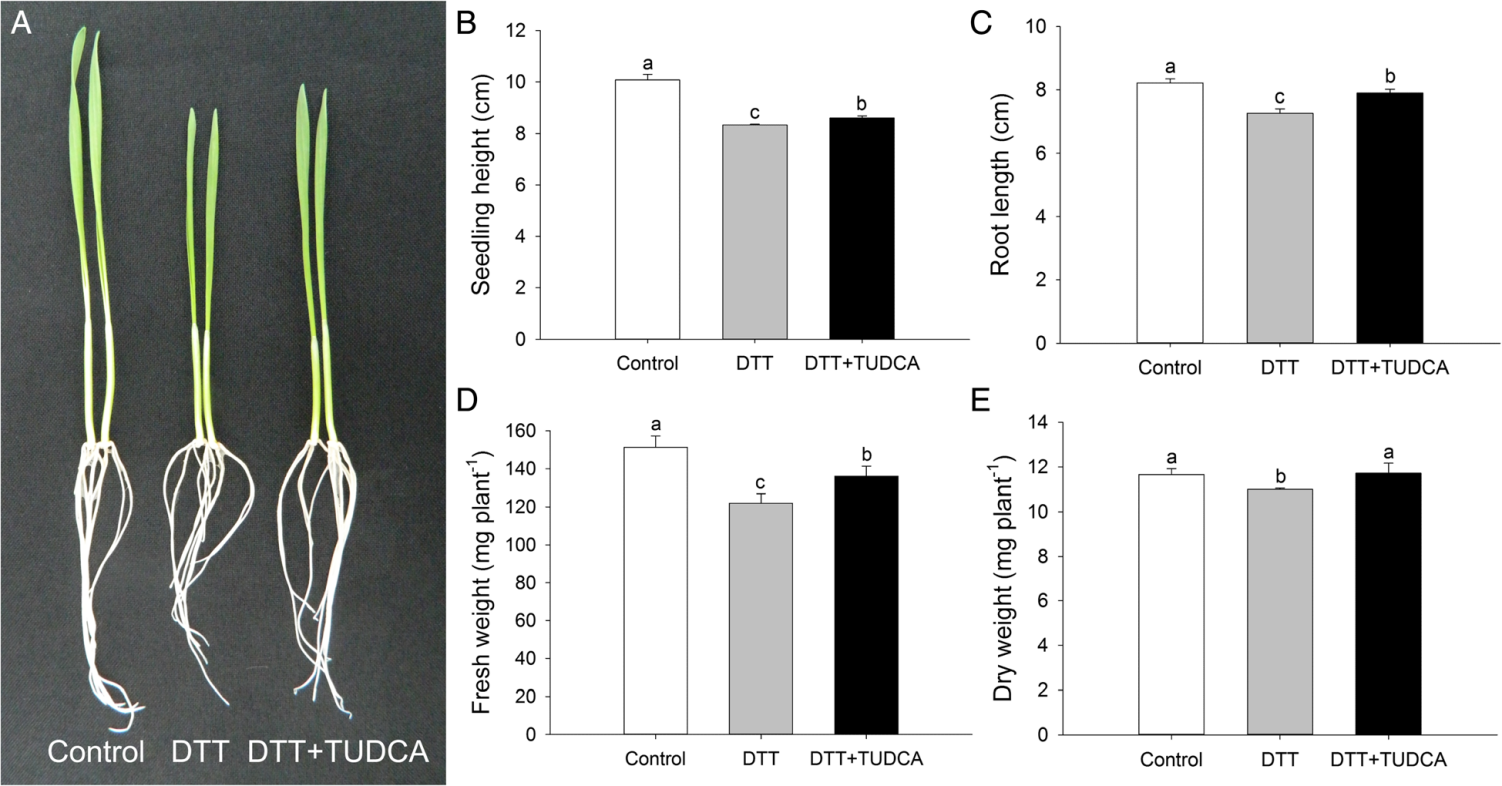
Morphological changes of wheat seedlings under different treatments after two days. (**a**) Whole view of wheat seedlings. (**b**, **c**) Seedling height and root length. (**d**, **e**) Fresh weight and dry weight. Different letters indicate significant difference among treatments at the 0.05 significance level based on Duncan’s multiple range tests. Bars represent the mean ± SD (*n* = 3)

To confirm the alleviating effects of TUDCA on DTT-induced ER stress, we observed and analyzed the morphological and physiological changes of wheat under three treatments: control, DTT, and DTT + TUDCA. The morphological changes were captured after two days’ treatments. Compared with those of the control, DTT treatment inhibited plant height, with the inhibition significantly alleviated by co-treatment with TUDCA (Fig. 1A). For measurements of seedling height and root length, significant differences were identified among the three treatments. Compared with the control, the seedling height and root length decreased 17.3 and 11.6%, respectively, in the DTT treatment but decreased 14.6 and 4.0%, respectively, in the DTT + TUDCA treatment (Figs. 1B, C). Simultaneously, the fresh weight and dry weight were also measured. Compared with the control, the fresh weight of the DTT treatment decreased 19.4%, whereas the decrease was 10.1% in DTT + TUDCA (Fig. 1D). Additionally, biomass in the DTT treatment decreased 5.7%, whereas in DTT + TUDCA, biomass increased 0.5% (Fig. 1E).

**Fig. 2:**
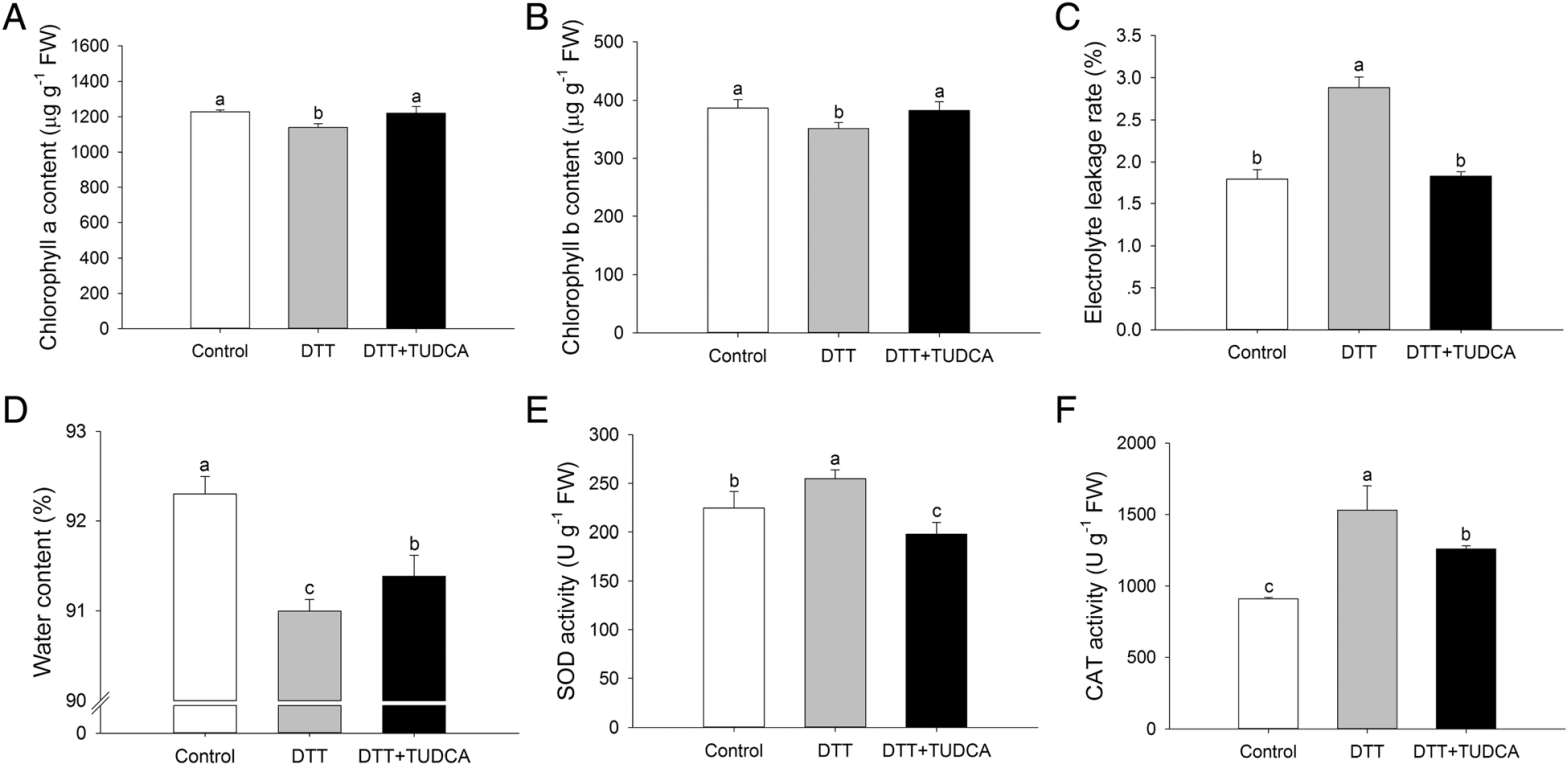
Physiological and biochemical changes under different treatments after two days. (**a**) Chlorophyll a content. (**b**) Chlorophyll b content. (**c**) Electrolyte leakage rate. (**d**) Water content. (**e**) SOD activity. (**f**) CAT activity. Different letters indicate significant difference among treatments at the 0.05 significance level based on Duncan’s multiple range tests. Bars represent the mean ± SD (n = 3)

To understand the physiological mechanism of the TUDCA alleviation of DTT-induced ER stress, we examined the physiological and biochemical indices after two days of treatments. To evaluate plant health and cell damage of wheat seedlings under DTT treatment, chlorophyll a and b contents and the electrolyte leakage rate were monitored in the presence or absence of TUDCA. Compared with the control, the chlorophyll a and b contents in the DTT treatment decreased 7.3 and 9.1%, respectively, whereas the decrease was only 0.7 and 1.1%, respectively, under DTT + TUDCA co-treatment (Figs. 2A, B). Additionally, compared with the control, the electrolyte leakage rate in the DTT treatment increased 60.8%, whereas with DTT + TUDCA co-treatment, the increase was only 2.0% (Fig. 2C). The water content and activity of antioxidant enzymes among the three treatments also had noteworthy differences. Compared with the control, the water content in the DTT treatment decreased 1.4%, with the decrease 1.0% under DTT + TUDCA co-treatment (Fig. 2D). Additionally, compared with the control, SOD and CAT activity of the DTT treatment increased 13.4 and 67.8%, respectively, whereas the increase was − 12.0 and 38.4%, respectively, under DTT + TUDCA co-treatment (Figs. 2E, F). Moreover, we monitored the dynamic changes of several physiological and biochemical parameters, and similar results were obtained (Additional file 5: Figure S3).

**Fig. 3:**
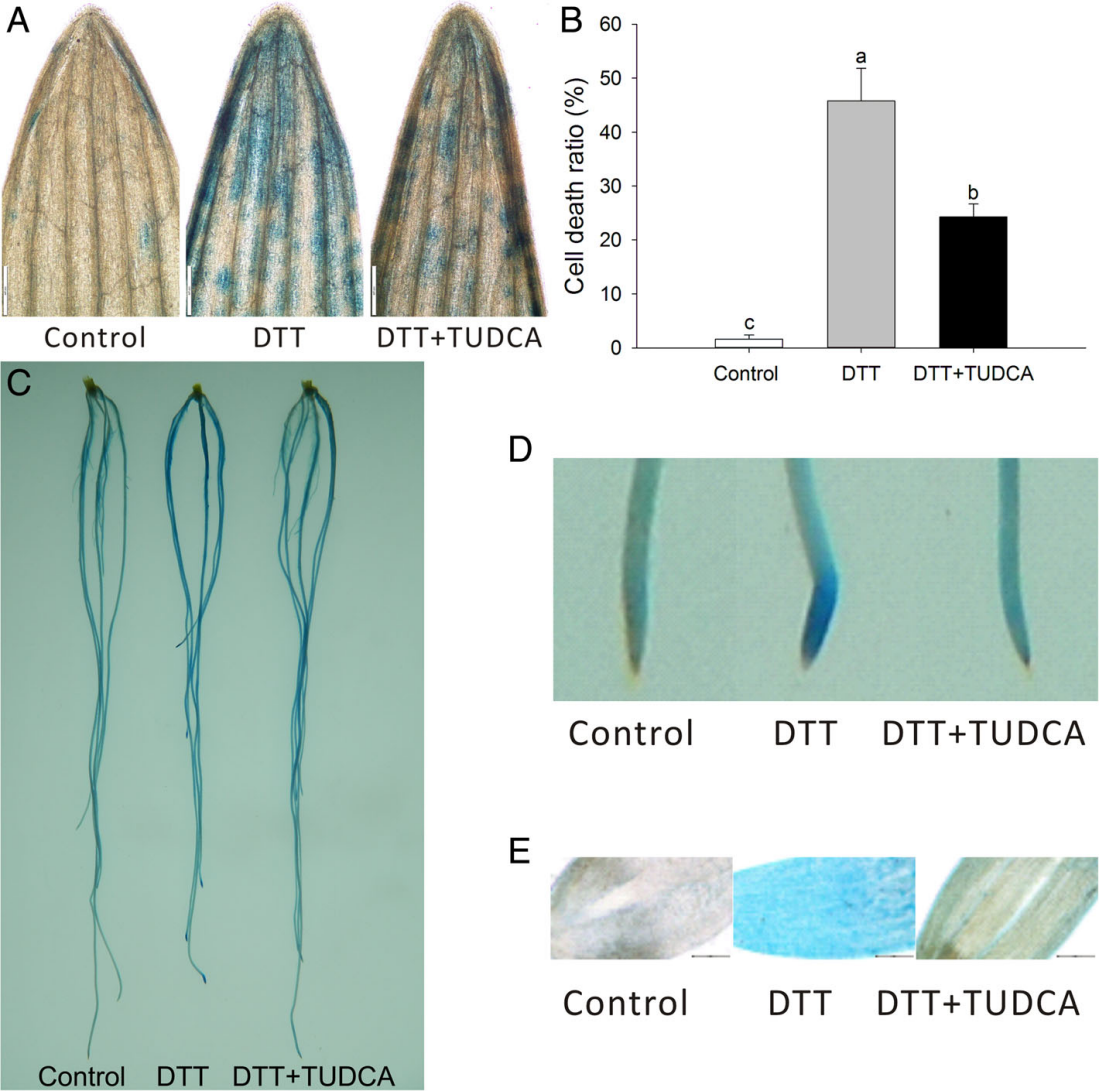
Comparison of wheat leaf and root under different treatments by trypan blue staining. (**a**) Trypan blue staining in leaf after 4-day’s treatment under microscope (X4). (**b**) Cell death ratio of leaf after 4-day’s treatment. (**c**, **d**) Trypan blue staining in seedling root after 1-day’s treatment under digital camera (**c**) Root system; (**d**) Root tip. (**e**) Root tip under microscope (X10). Bar = 500 μm in A and bar = 200 μm in E. Different letters of B indicate significant difference among treatments at the 0.05 significance level based on Duncan’s multiple range tests. Bars represent the mean ± SD (n = 3)

We also observed cell death in wheat seedlings using trypan blue staining. Under a normal condition, almost no cell death occurred in the leaf-tip zone. However, after treatment with DTT, approximately half the cells died at the tips of leaves, whereas under DTT + TUDCA co-treatment, the number of cell deaths reduced by half (Figs. 3A, B). We also observed the same phenomenon using trypan blue staining in roots (Figs. 3C, D). Based on microscopic observations of the root tip, the cell death under DTT was severe; whereas the number of dead cells was markedly reduced by TUDCA co-treatment (Fig. 3E), indicating that TUDCA alleviated DTT-induced ER stress in wheat.

### Identification of differentially expressed genes

**Fig. 4:**
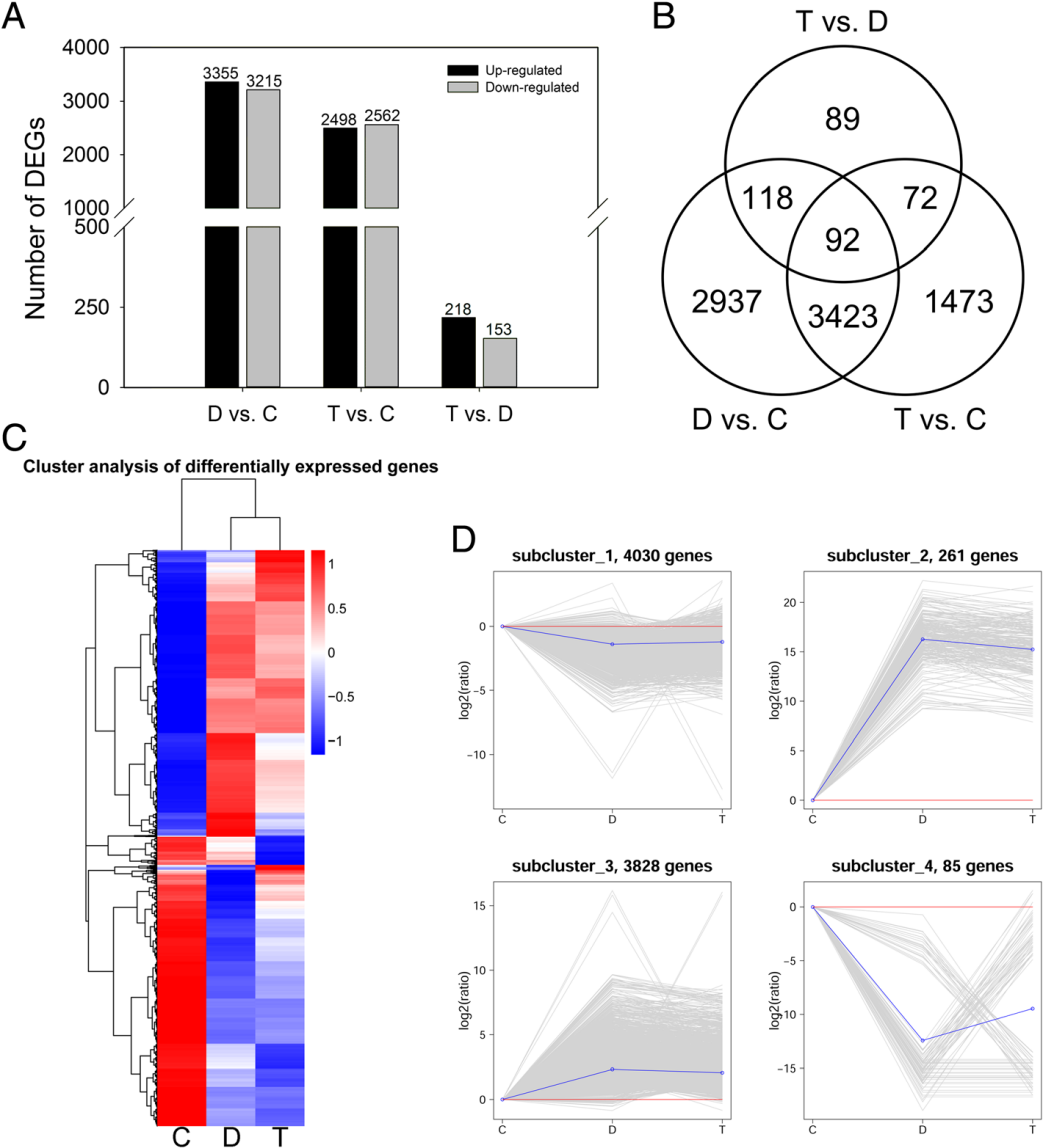
Summary of RNA sequencing data. (**a**) Histogram of DEGs number under different groups. (**b**) Venn diagram showing the number of DEGs between every two groups and the number of joint DEGs. (**c**) Hierarchical clustering of DEGs, using the RNA sequencing data derived from three treatments based on log_10_ (FPKM+ 1) values. The red bands indicate the higher expression, and the blue bands show the lower expression. (**d**) Gene expression pattern analysis of DEGs. The four subclusters obtained by K-means algorithm. Expression ratios are expressed as log2 values. The X-axis represents different treatments and the Y-axis represents the relative gene expression. C, control; D, DTT; T, DTT + TUDCA

To identify the genes involved in regulating ER stress responses in wheat, RNA-seq was performed. After removing contaminated and low-quality sequences and trimming the adapter sequences, 592 million (88.93 GB) clean reads were obtained with an average of 65 million reads (9.88 GB) for each sample (Additional file 3: Table S1). All reads were mapped onto the reference wheat genome. In the control, DTT and DTT + TUDCA treatments, 66.51, 66.11 and 63.07% of the total reads from RNA-seq data were uniquely mapped to the genome, respectively (Table 1). We used FPKM to normalize the expression level of genes, and genes with FPKM values larger than 1 were considered to be expressed in this study (Additional file 6: Table S3). We screened 6570, 5060, and 371 DEGs in pairs of DTT (D) vs. control (C), DTT + TUDCA (T) vs. control (C), and DTT + TUDCA (T) vs. DTT (D), respectively (Fig. 4A). A total of 8204 DEGs were obtained from the three treatment groups. The Venn diagram showed that only 92 DEGs were commonly expressed across the three treatment groups (Fig. 4B). We performed a hierarchical clustering of all DEGs based on their log_10_ (FPKM+ 1) values from all three treatments (Fig. 4C). The expression profiles of DEGs were illustrated by a cluster analysis based on the K-means algorithm, and four expression patterns of DEGs were obtained under the three treatments (Fig. 4D). Compared with the control, the expression levels of DEGs in clusters 1 and 4 decreased under DTT treatment, while the expression levels of DEGs in clusters 2 and 3 increased under DTT treatment. However, under DTT + TUDCA co-treatment, the expression levels of DEGs in clusters 2 reduced but those in clusters 4 enhanced, with others in clusters 1 and 3 slightly changed. The most abundant DEGs of clusters 1, 3 and 4 were mainly involved in “metabolic pathways”, while the DEGs of cluster 2 were mainly participated in “plant hormone signal transduction”, “protein processing in endoplasmic reticulum” and “biosynthesis of secondary metabolites”.

**Table 1:** RNA Sequencing overview

Sample	C	D	T
Total reads	69,442,035	66,764,717	61,407,087
Total mapped	53,580,308 (77.16%)	50,946,122 (76.31%)	44,975,015 (73.24%)
Multiple mapped	7,391,229 (10.64%)	6,805,826 (10.19%)	6,245,750 (10.17%)
Uniquely mapped	46,189,079 (66.51%)	44,140,296 (66.11%)	38,729,266 (63.07%)
Non-splice reads	28,691,618 (41.32%)	27,197,221 (40.74%)	24,721,906 (40.26%)
Splice reads	17,497,460 (25.20%)	16,943,074 (25.38%)	14,007,360 (22.81%)

Notes: The data represent the average value of three biological replicates. C, control; D, DTT; T, DTT + TUDCA

### Differentially expressed genes induced by DTT

**Fig. 5:**
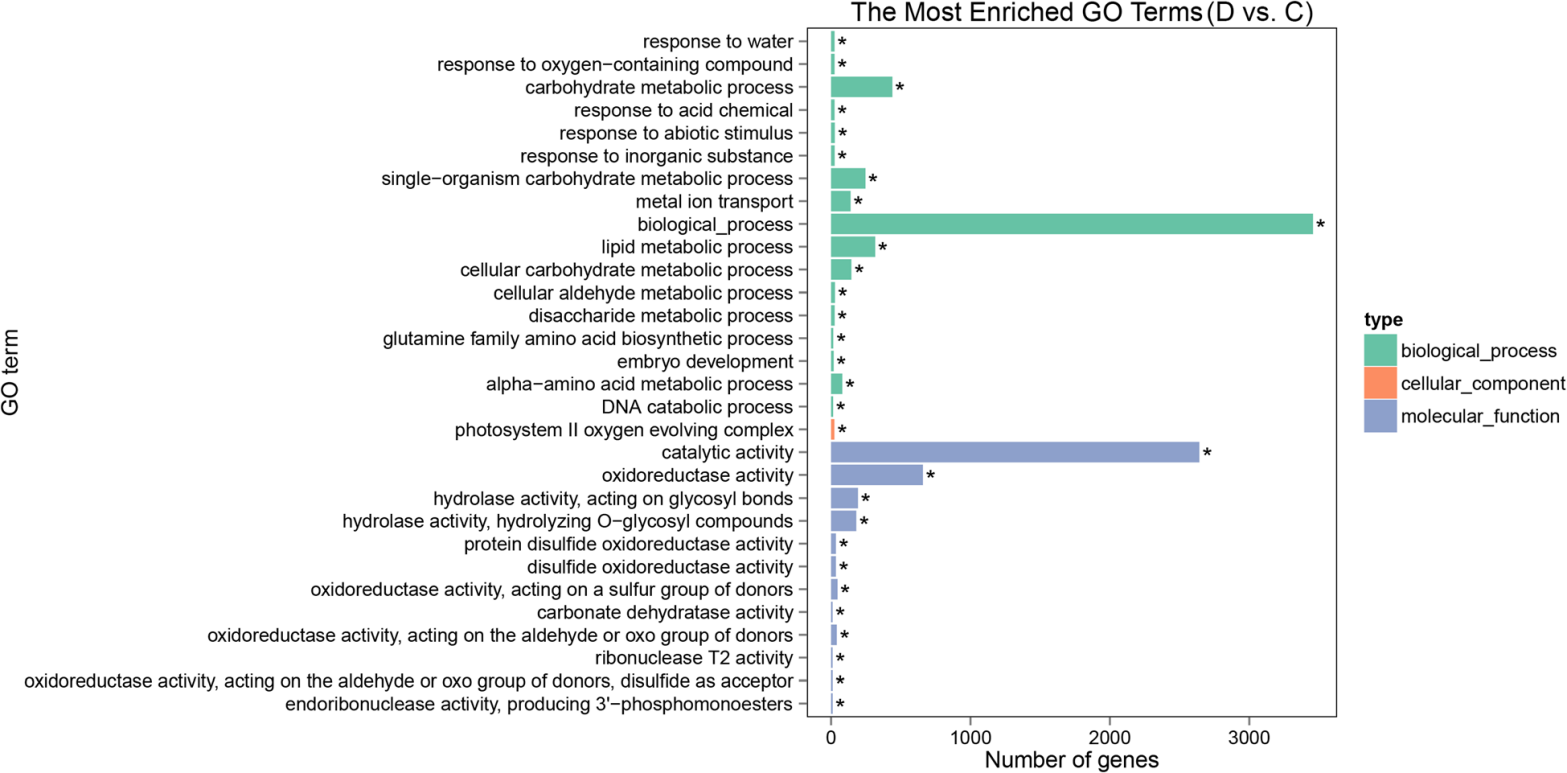
GO classification of DEGs under group “D vs. C”. The top 30 GO terms were determined by the corrected *P*-values. The X-axis indicates the number of genes, and the Y-axis is the enriched GO terms. Different colors are used to distinguish biological process, cell component, and molecular function, with “*” as the significantly enriched GO terms. C, control; D, DTT

A total of 6570 DEGs were identified under group “D vs. C”, with 3355 up-regulated and 3215 down-regulated (Fig. 4A). Using GO analysis, the 6570 DEGs were categorized into 3070 GO terms, including three classifications: “biological processes” (1748), “cellular components” (362) and “molecular functions” (960) (Additional file 7: Table S4). Based on the corrected *P*-values, we selected the 30 most enriched GO terms (Fig. 5). Among these terms, the primary terms of the biological processes category were “biological process” (3456 genes), “carbohydrate metabolic process” (418 genes) and “lipid metabolic process” (317 genes). In the cellular component category, the only enriched GO term was “photosystem II oxygen evolving complex” (24 genes). The primary terms of the molecular functions category were “catalytic activity” (2642 genes), “oxidoreductase activity” (659 genes), “hydrolase activity” (374 genes) and “protein disulfide oxidoreductase activity” (35 genes). Moreover, enriched GO terms were displayed by directed acyclic graph (DAG), which showed associated GO terms together. In terms of biological process, cellular component, and molecular function categories, the most significantly enriched GO terms were “response to water” (GO: 0009415), “photosystem II oxygen evolving complex” (GO: 0009654), and “catalytic activity” (GO: 0003824), respectively (Additional file 8: Figure S4).

**Fig. 6:**
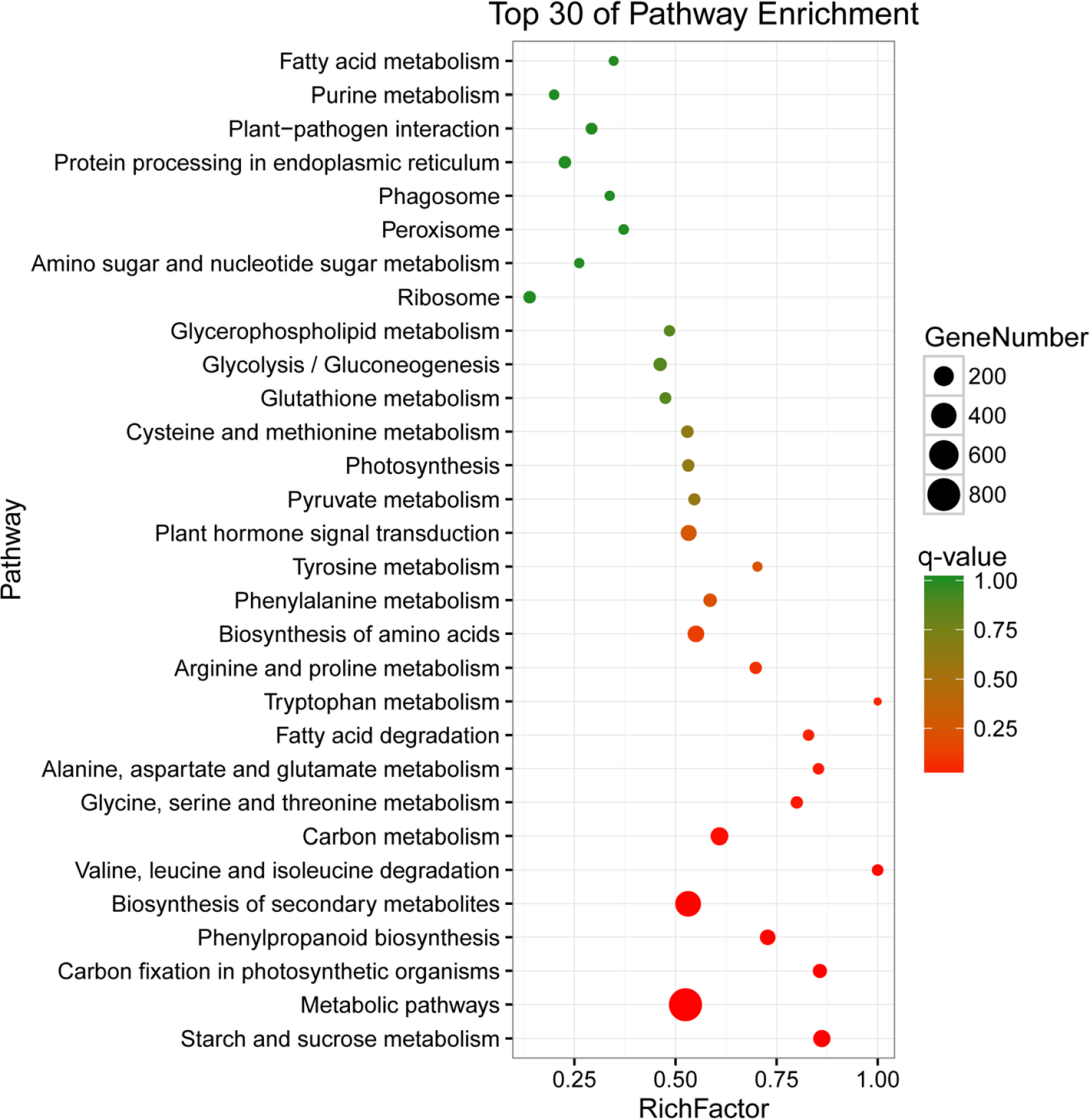
KEGG pathway classification of DEGs under group “D vs. C”. The Y-axis represents the pathway name, and the X-axis represents the rich factor. The size of the dot represents the number of DEGs in the pathway, and the color of the dot corresponds to a differently *q*-value (corrected *P*-value) range. The senior bubble was obtained by OmicShare tools, a free online platform for data analysis (www.omicshare.com/tools)

To investigate the biological pathways that were actively involved in DTT-induced ER stress, we mapped DEGs to reference canonical pathways in the KEGG database. The DEGs under group “D vs. C” were assigned to 110 KEGG pathways. Based on abundantly enriched DEG numbers, we listed the top 30 pathways. The “metabolic pathway” (813 genes), “biosynthesis of secondary metabolites” (414 genes), and “carbon metabolism” (137 genes) pathways were the most highly represented groups. From the listed pathways, we were most interested in “protein processing in endoplasmic reticulum” (46 genes), “plant hormone signal transduction” (98 genes), and “photosynthesis” (42 genes) pathways (Fig. 6).

**Fig. 7:**
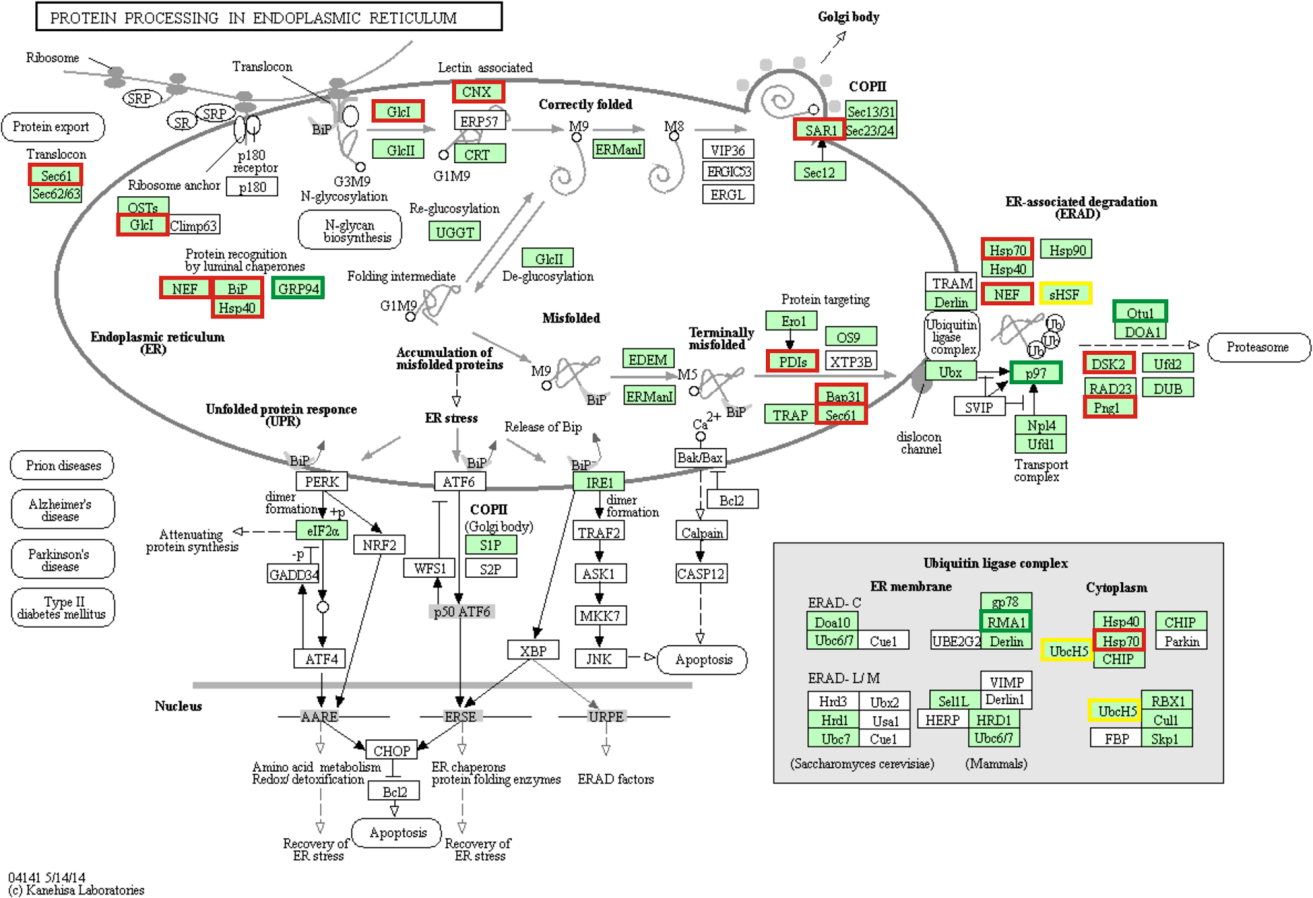
DEGs relevant to the “protein processing in endoplasmic reticulum” pathway under group “D vs. C”. Red boxes refer to genes whose associated DEGs were un-regulated under DTT treatment, and green boxes refer to genes whose associated DEGs were down-regulated. Boxes with yellow color indicate genes that might have isoforms

**Fig. 8:**
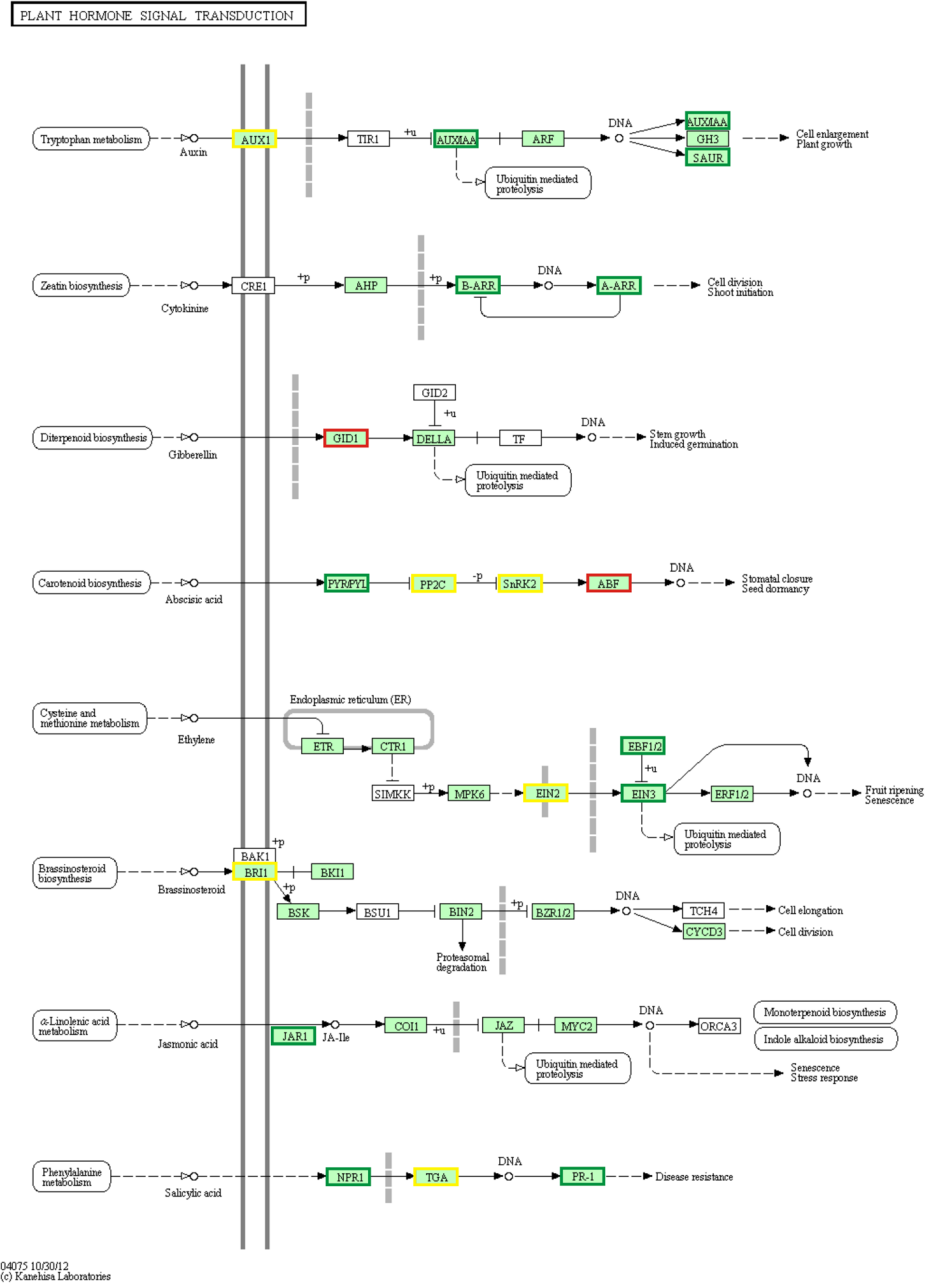
DEGs relevant to the “plant hormone signal transduction” pathway under group “D vs. C”. Red boxes refer to genes whose associated DEGs were un-regulated under DTT treatment, and green boxes refer to genes whose associated DEGs were down-regulated. Boxes with yellow color indicate genes that might have isoforms

In the “protein processing in endoplasmic reticulum” pathway, 46 DEGs were identified within group “D vs. C”, primarily including molecular chaperones, such as Bips, GRP94, CNX, PDIs, Hsp40s, Hsp70s and sHSFs, and ubiquitin-ligase complexes, such as RMA1 and UbcH5 (Fig. 7 and Additional file 9: Table S5). The “plant hormone signal transduction” pathway was represented by 98 genes and contained salicylic acid (SA)-, ethylene (ET)-, auxin-, jasmonic acid (JA)-, gibberellin (GA)-, abscisic acid (ABA)-, cytokinin-, and brassinosteroid (BR)-associated signaling genes that were induced via ER stress (Fig. 8 and Additional file 10: Table S6). Additionally, for the “photosynthesis” pathway, 42 genes were identified, primarily containing photosystem I subunits (17 genes), photosystem II oxygen-evolving enhancer proteins (9 genes), and ferredoxin (5 genes), among others.

### Differentially expressed genes mediated by TUDCA

**Fig. 9:**
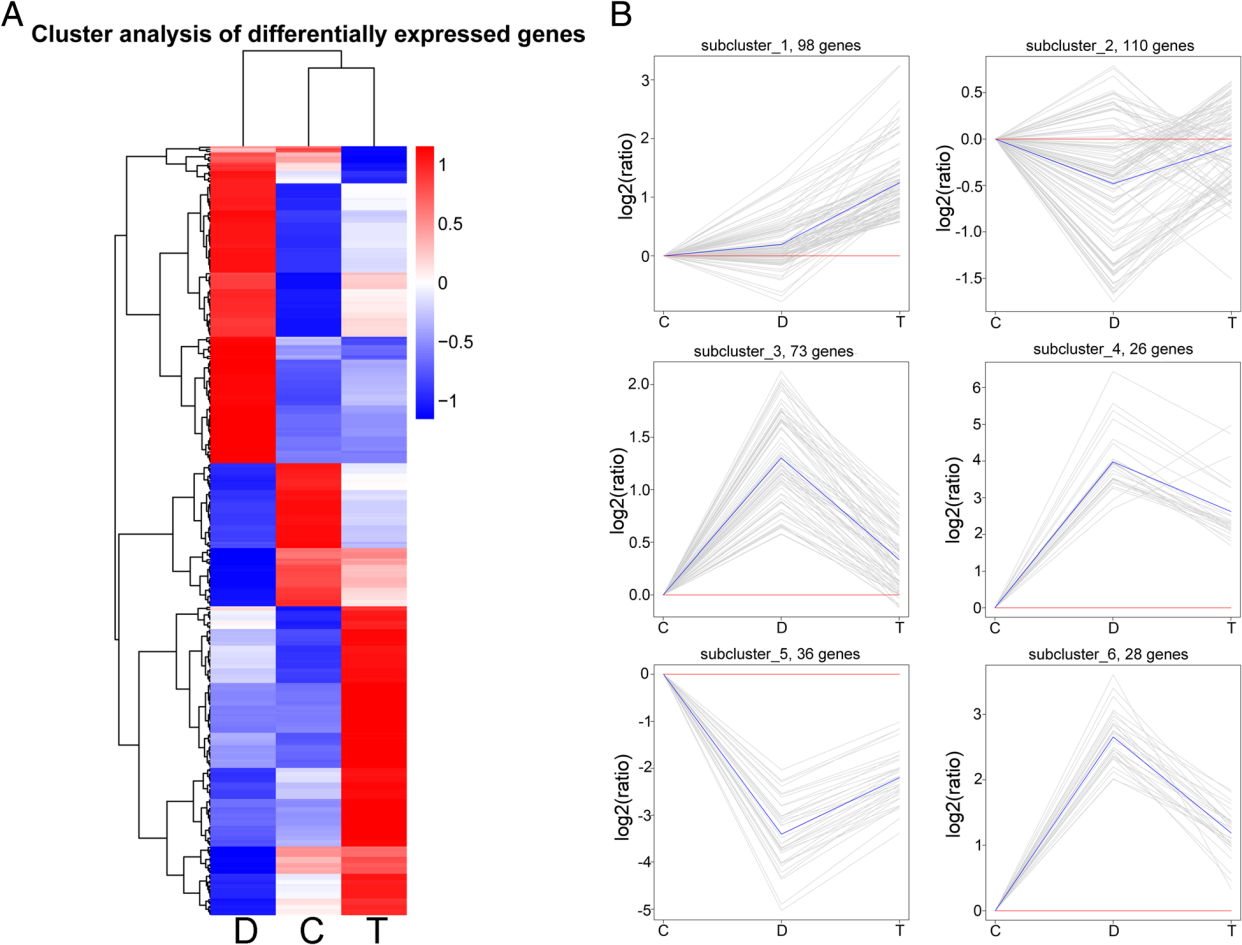
Cluster analysis of 371 DEGs under group “T vs. D” among three treatments. (**a**) Hierarchical clustering of 371 DEGs under group “T vs. D”, using the RNA sequencing data derived from three treatments based on log_10_ (FPKM+ 1) values. The red bands indicate the higher expression, and the blue bands show the lower expression. (**b**) Gene expression pattern analysis of 371 DEGs between DTT and DTT + TUDCA under different treatments. The 6 subclusters obtained by h-cluster algorithm. Expression ratios are expressed as log2 values. The X-axis represents different treatments and the Y-axis represents the relative gene expression. C, control; D, DTT; T, DTT + TUDCA

A total of 371 DEGs were identified under group “T vs. D”, with 218 up-regulated and 153 down-regulated (Fig. 4A). These DEGs were specifically mediated by TUDCA and therefore may play central roles in the ER stress-alleviating process. We performed a hierarchical clustering of the 371 DEGs to show their expression patterns under the three treatments (Fig. 9A), and the heatmap showed the expression pattern of group “T” was closer to that of the control. We also obtained the expression profiles of the 371 DEGs using a cluster analysis based on the h-cluster algorithm and found the DEGs were primarily classified into six subclusters (Fig. 9B). Compared with the control, the expression levels of DEGs belonging to clusters 2 and 5 decreased under DTT treatment and increased under DTT + TUDCA co-treatment, while DEGs belonging to clusters 3, 4 and 6 showed a reverse trend under DTT treatment and DTT + TUDCA co-treatment, respectively. Among these clusters, cluster 1 were mainly involved in “protein processing in endoplasmic reticulum” and “protein export”, and cluster 3 were involved in “protein processing in endoplasmic reticulum” and “plant hormone signal transduction”, and other clusters were mostly involved in “metabolic pathways”.

**Fig. 10:**
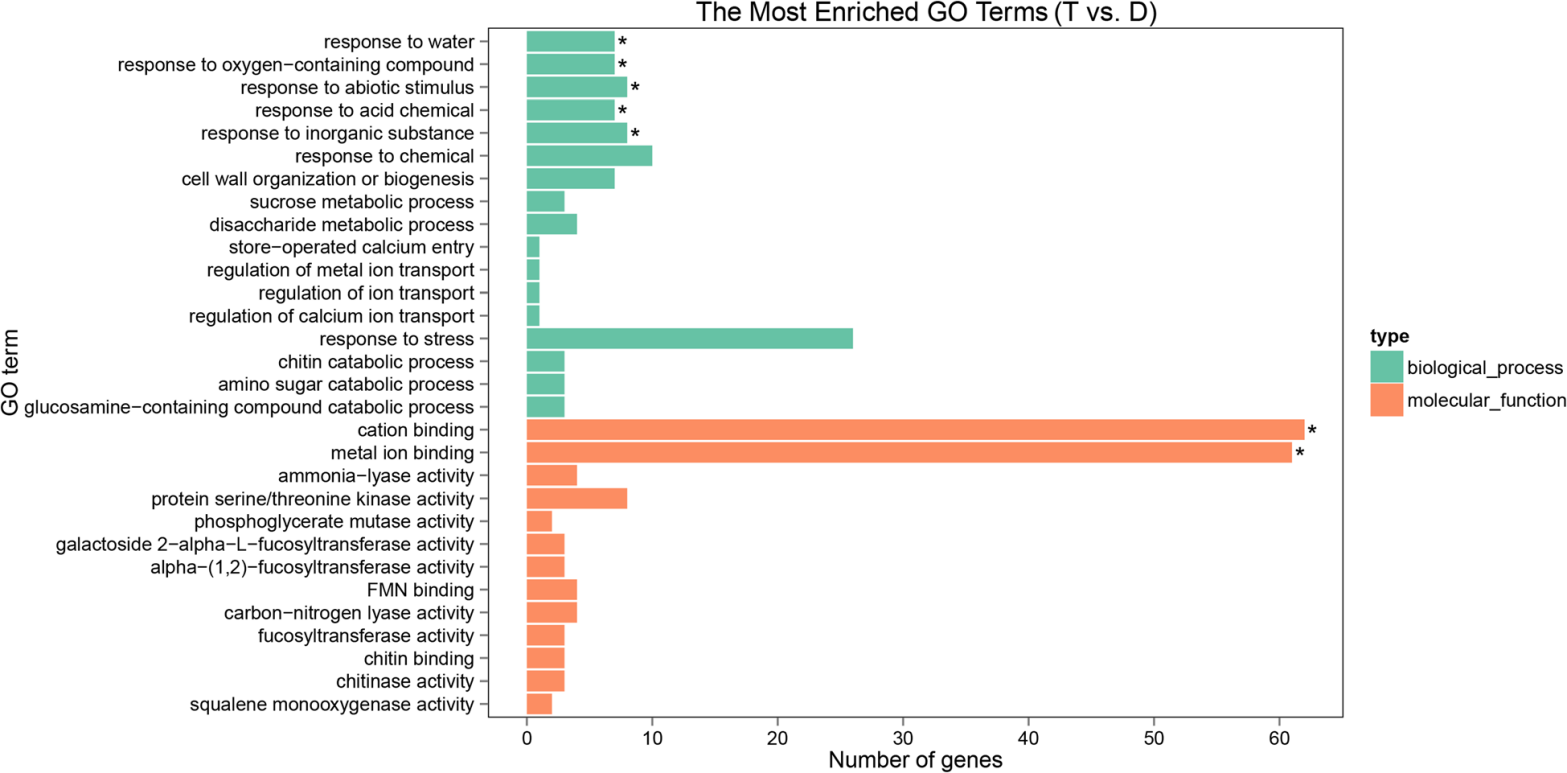
GO classification of DEGs under group ‘T vs. D’. The top 30 GO terms were determined by the corrected *P*-values. The X-axis indicates the number of genes, and the Y-axis is the enriched GO terms. Different colors are used to distinguish biological process, cell component, and molecular function, with “*” as the significantly enriched GO terms. D, DTT; T, DTT + TUDCA

**Fig. 11:**
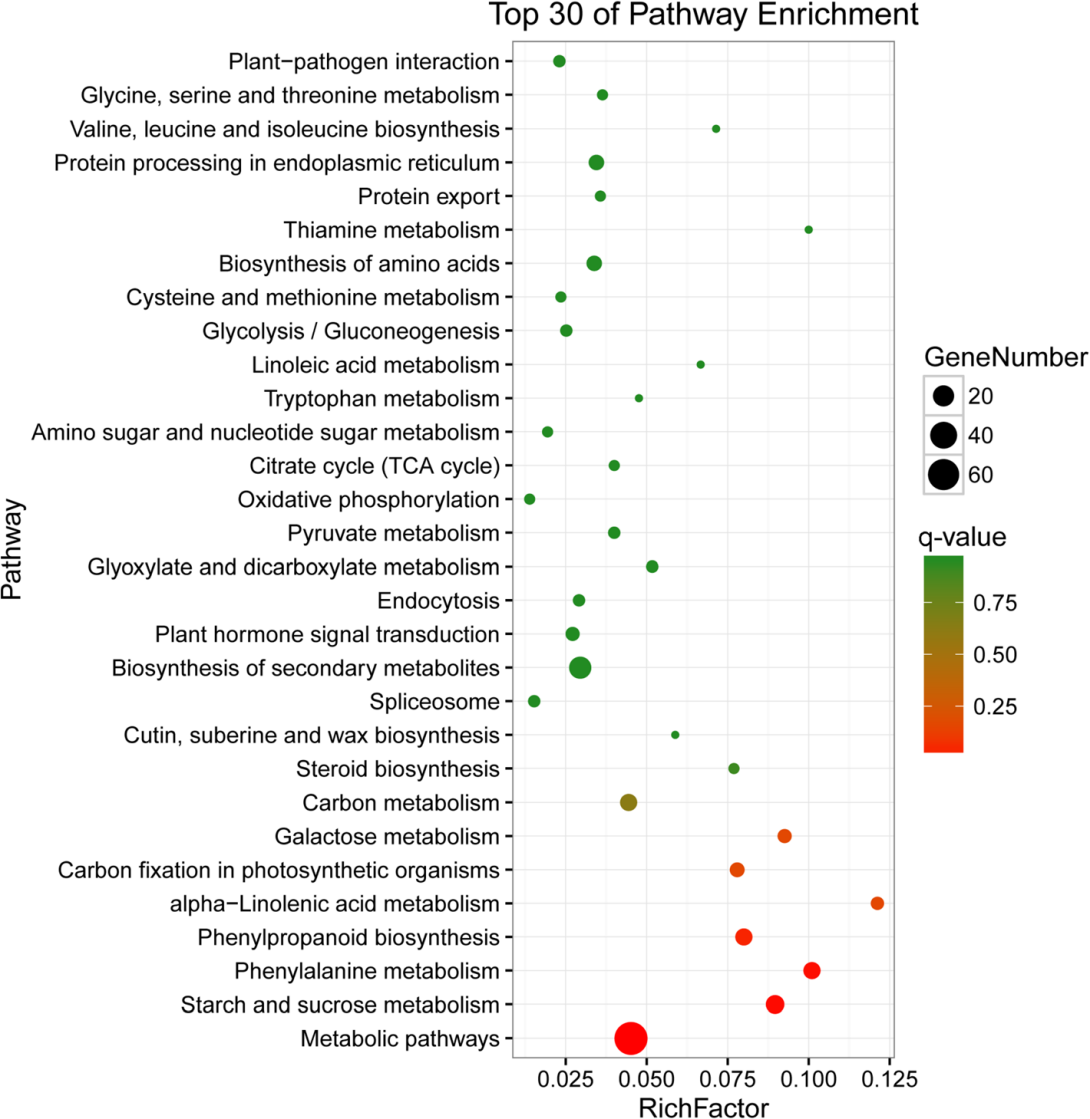
KEGG pathway classification of DEGs under group “T vs. D”. The Y-axis represents the pathway name, and the X-axis represents the rich factor. The size of the dot represents the number of DEGs in the pathway, and the color of the dot corresponds to a different *q*-value (a corrected *P*-value) range. The senior bubble was obtained by OmicShare tools, a free online platform for data analysis (www.omicshare.com/tools)

Using GO analysis, the 371 DEGs were categorized into 924 GO terms, including “biological processes” (515), “cellular components” (125) and “molecular functions” (284) (Additional file 11: Table S7). The top 30 most enriched GO terms were selected for exhibition based on the corrected *P*-values (Fig. 10). Among the enriched terms, the primary GO terms in the biological processes class were “response to stress” (26 genes) and “response to chemical” (10 genes); the primary terms in the molecular function category were “cation binding” (62 genes) and “metal ion binding” (61 genes). Based on the DAG, in the biological process and molecular function classes, the most significantly enriched GO terms were “response to water” (GO: 0009415) and “cation binding” (GO: 0043169), respectively (Additional file 12: Figure S5). We mapped the 371 DEGs to reference canonical pathways in the KEGG database and obtained 46 enriched pathways. Among the top 30 pathways, the greatest representation was the “metabolic pathways” with 70 DEGs (Fig. 11). Additionally, in the “protein processing in endoplasmic reticulum” pathway, 7 genes were enriched, containing molecular chaperones and ubiquitin-ligase complexes. In the “plant hormone signal transduction” pathway, 5 genes were enriched, including four PP2Cs and one B-ARR.

### Identification of transcription factors

**Fig. 12:**
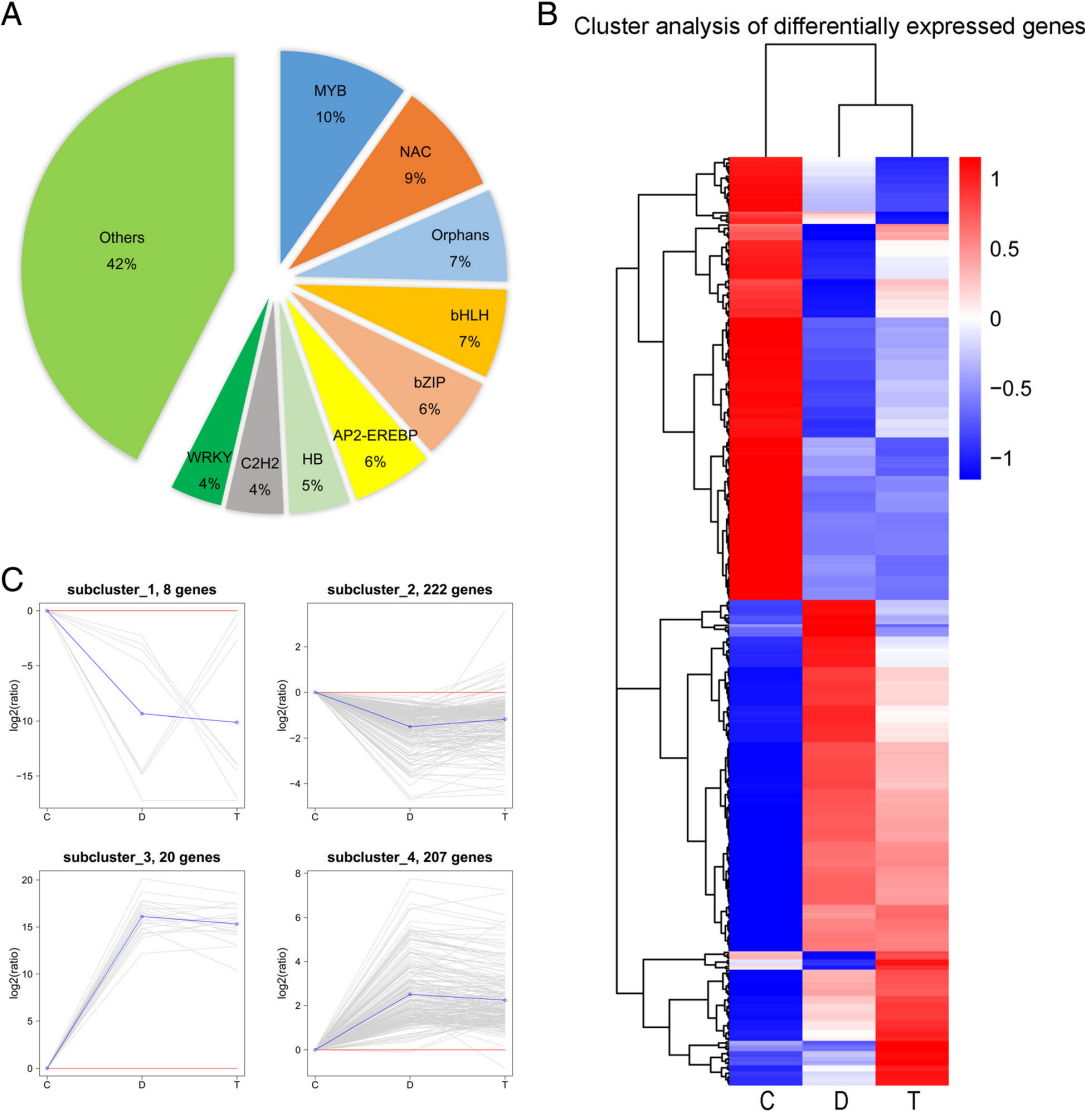
Summary of transcription factor data. (**a**) Pie chart showing top 9 TF families which contain more than 50% of differentially expressed TFs. (**b**) Hierarchical clustering of TFs, using the RNA sequencing data derived from three treatments based on log_10_ (FPKM+ 1) values. The red bands indicate the higher expression, and the blue bands show the lower expression. (**c**) Gene expression pattern analysis of TFs. The 4 subclusters obtained by K-means algorithm. Expression ratios are expressed as log2 values. The X-axis represents different treatments and the Y-axis represents the relative gene expression. C, control; D, DTT; T, DTT + TUDCA

To identify the TFs involved in the regulation of plant response to ER stress, 457 wheat TFs distributed in 47 families were identified using iTAK software (Additional file 13: Table S8). Among the TFs, the top 9 families accounted for more than half of the total stress responsive TFs, which included MYB, NAC, orphans, bHLH, bZIP, AP2/ERF, HB, C2H2, and WRKY (Fig. 12A). To understand the gene expression patterns of these TFs under the three treatments, we constructed a hierarchical clustering map (Fig. 12B). Further, we classified these TFs into four subclusters based on the K-means algorithm (Fig. 12C). Those in clusters 2 and 4, including 429 DEGs, were mainly encoding bHLH, MYB, bZIP, NAC and WRKY TFs, in cluster 1 were mainly MYB TFs and in Clusters 3 were mainly C2H2 TFs.

### Validation of RNA sequencing data

**Fig. 13:**
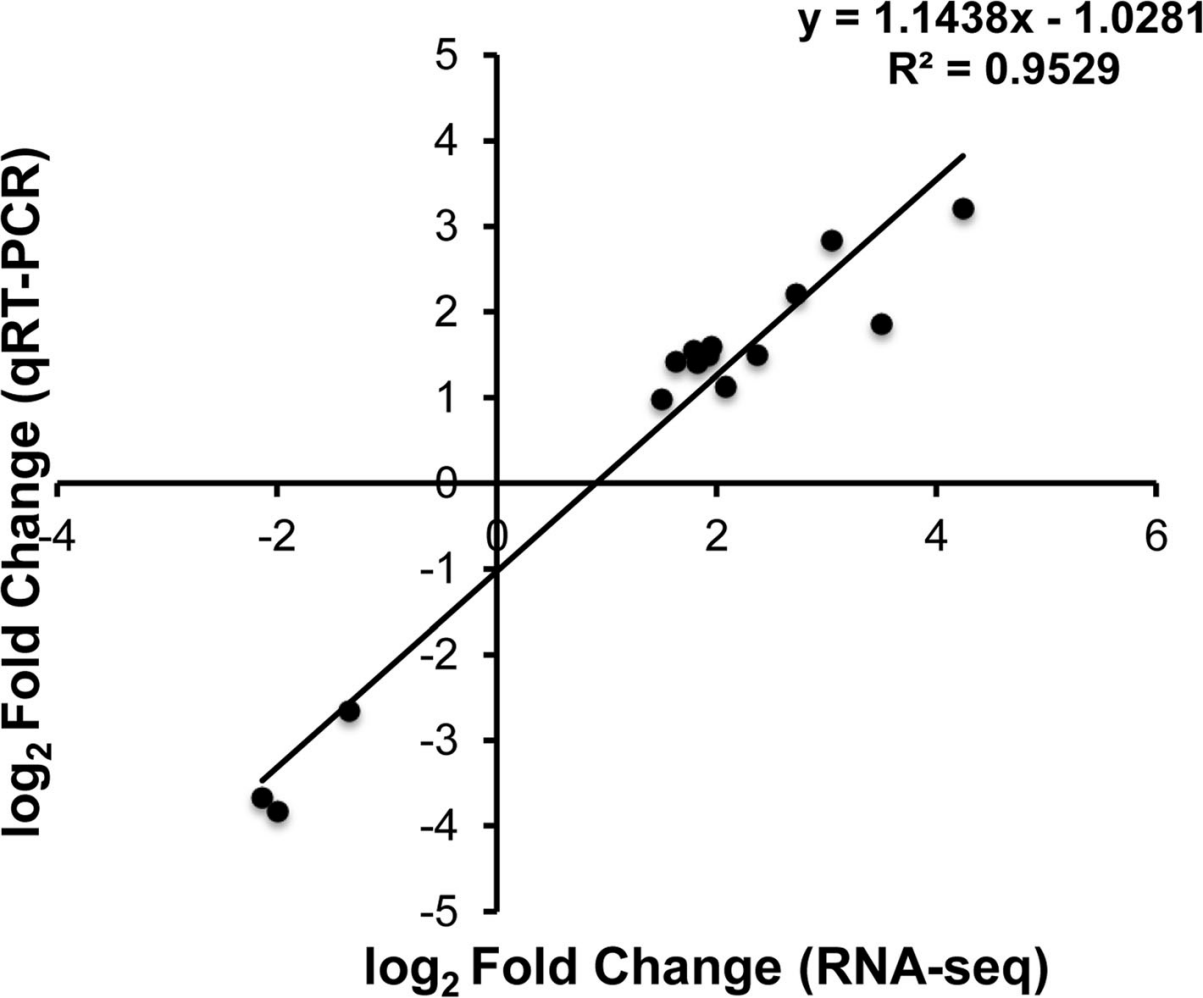
Validation of RNA sequencing data using qRT-PCR. The log_2_ fold changes between group “D vs. C” or group “T vs. D” (Y-axis), were plotted against the log_2_ fold changes of the same comparison, and determined through RNA-seq (X-axis). The function of the regression line and the *R*^*2*^ are given

To validate the data determined by RNA sequencing, qRT-PCR was performed with 15 randomly chosen genes. The results of qRT-PCR were generally consistent with the RNA-seq data, and the correlation coefficient was very high (*R*^*2*^ = 0.95), which confirmed the data of RNA-seq (Fig. 13).

## Discussion

### Potential candidate genes conferring wheat ER stress response

In this study, we analyzed the responses of wheat seedlings to ER stress from four biological levels: morphological, physiological, cytological and molecular, and we identified the genes likely involved in the regulation of stress.

#### Genes related to protein processing in ER

In the process of protein folding, molecular chaperones are indispensable. The aforementioned molecular chaperones, such as Bip, CNX/CRT, GRP94, and PDIs, play critical roles in protein folding, particularly when cells are subjected to malfolded proteins and unassembled complexes. Under numerous stresses, *Bip* expression is significantly up-regulated, including ER stress agents such as TM and DTT [4, 21, 25], drought [50], cold [51], Cd^2+^ [5] and insect and pathogen attack [1]. Overexpression of *Bip* confers drought tolerance in soybean (*Glycine max*) [52] and tobacco (*Nicotiana tabacum*) [52, 53] and increases the tolerance to Cd^2+^ in tobacco [5]. In this study, we found the number of chaperones accounted for more than half of the 66 DEGs in the “protein processing in endoplasmic reticulum” pathway within the three treatment groups (Additional file 14: Table S9). Among these DEGs, Bip genes were significantly up-regulated under DTT, and the expression was even higher after co-treatment with TUDCA; additionally, we detected the dynamic changes of Bip genes and similar results were obtained (Additional file 14: Table S9 and Additional file 15: Figures S6A-B), implying a role in the regulation of wheat response to ER stress.

The expression of *SHD,* the only ortholog of *GRP94* in *Arabidopsis,* was up-regulated after 2 and 4 h of treatment with ER stress agents TM or DTT [22, 27]. However, in the present study, compared with the control, we found *GRP94* expression was down-regulated under DTT treatment in wheat at 48 h and was up-regulated after TUDCA co-treatment (Additional file 14: Table S9 and Additional file 15: Figure S6C). Therefore, we further studied the expression of *GRP94* under different time points, and we found *GRP94* was up-regulated under DTT treatment at 4 h and 24 h, and then down-regulated at 48 h and 96 h. However, under DTT + TUDCA co-treatment, *GRP94* was up-regulated at 4 h, but down-regulated at 24 h, and then up-regulated at 48 h and 96 h (Additional file 15: Figure S6C).

CNX and CRT are ER chaperone proteins, bind calcium ions and participate in protein folding. Rice CNX (*Os-CNX*) is induced by various abiotic stresses, and overexpression of *Os-CNX* in tobacco confers drought tolerance [54]. Furthermore, wheat CRT (*Ta-CRT*) is induced by drought, and overexpression of *Ta-CRT* in tobacco plants increases drought resistance [55]. In our study, *Ta-CRT* was not induced by DTT treatment at 48 h, but the homologous ER-lumenal protein, *Ta-CNX*, was significantly up-regulated under DTT and further up-regulated after co-treatment with TUDCA (Additional file 14: Table S9 and Additional file 16: Figure S7A).

PDIs are molecular chaperones that catalyze the formation of disulfide bonds between unfolded proteins. In our study, eight genes related to PDI were obtained in wheat. Except for *Traes_6AS_5896DC565*, the other seven genes exhibited the same trend: *PDIs* were markedly up-regulated under DTT and further up-regulated under TUDCA co-treatment (Additional file 14: Table S9, Additional file 15: Figure S6D and Additional file 16: Figures S7B-C).

Other chaperones, such as DnaJ and those in Hsp70 and Hsp20 families, participate in ERAD (Fig. 7). Except for chaperones, other genes were related to ubiquitin-ligase complexes, including two E3 ubiquitin-protein ligase RNF5 genes (RMA1) and two ubiquitin-conjugating enzyme E2 D/E genes (UbcH5). Compared with the control, *RMA1s* (*Novel07430* and *Novel07785*) were down-regulated under DTT and up-regulated after co-treatment with TUDCA, whereas *UbcH5s* (*Traes_6BS_72F59E261* and *Traes_6AS_E011BC5BB*) showed the opposite trend (Additional file 14: Table S9). These results indicate that molecular chaperones actively participate in the regulation of plant response to ER stress.

#### Photosynthesis-related genes

A total of 158 photosynthesis-related genes were identified by RNA-seq. In addition to the aforementioned genes identified in the “photosynthesis” pathway, 65 DEGs were involved in “photosynthesis-antenna proteins” pathway. In this pathway, the DEGs were chlorophyll *a/b*-binding proteins, which primarily collect and transfer light energy to photosynthetic reaction centers and are down-regulated when plants are subjected to environmental stresses [56]. In this study, chlorophyll *a/b*-binding proteins were down-regulated under DTT but up-regulated after co-treatment with TUDCA (Additional file 16: Figures S7D-F). Correspondingly, chlorophyll *a* and *b* contents also showed a marked decrease under DTT treatment, whereas the effects were alleviated by TUDCA co-treatment (Figs. 2A, B). Furthermore, chlorophyll is the most important pigment in plant photosynthesis for the absorption and transmission of light energy, and the pathway of chlorophyll biosynthesis is completed by a series of enzymatic reactions. We identified 49 DEGs involved in “porphyrin and chlorophyll metabolism” pathway, and they were key enzyme genes in chlorophyll biosynthesis and might play critical roles in maintaining plant growth under stress conditions.

#### Antioxidant enzyme genes

One of the common responses when plants are subjected to a wide range of biotic and abiotic stresses is the generation of ROS [57], which cause oxidative damage to plants [58]. Fortunately, plants developed an antioxidant defense system, which primarily consists of antioxidant enzymes to scavenge ROS and protect cells from oxidative injury [58]. The over-accumulation of ROS can result in cytomembrane damage and even cell death [31, 58]. In this study, a total of 42 DEGs were identified that were related to antioxidant enzymes, including *PODs* (39 genes), *SODs* (2 genes) and *CAT* (1 gene). The relative expression of *SODs* was down-regulated under DTT treatment and up-regulated after DTT + TUDCA co-treatment. We monitored the dynamic changes of one SOD gene (*Traes_7AL_E14A72218*), and similar results were obtained over time (Additional file 15: Figure S6E). *CAT* expression exhibited a trend of continuous down-regulation. For *PODs*, the expression patterns were different under the three treatments (Additional file 16: Figures S7G-I). Correspondingly, the activity of antioxidant enzymes SOD and CAT increased under DTT treatment compared with that of the control, whereas activity level was eased by TUDCA co-treatment (Figs. 2E, F), indicating the ROS levels were reduced by TUDCA. Thus, these antioxidant enzyme genes may play critical roles in the process of wheat response to stress.

#### Plant hormone-related genes

A total of 318 genes were identified with involvement in plant hormone biosynthesis and signal transduction. Among these genes, 110 genes were involved in the signal transduction of plant hormones, with those related to ABA signaling pathway predominantly induced, including protein phosphatase 2Cs (PP2Cs), serine/threonine-protein kinase SRK2s (SnRK2s), ABA-responsive element binding factors (ABFs), auxin-responsive protein IAAs (AUX/IAAs), and abscisic acid receptor PYR/PYL families (PYR/PYLs). Researchers identify these genes as involved in stress responses. For example, *TaPP2C* expression is induced by water stress, and *TaPP2C* may be an early signal molecule [59]. ABFs act as bZIP TFs and play important roles in responding to environmental stresses. Similarly, *OsABF1* is induced by abiotic stress and increases stress signaling in rice [60], and *SnRK2* is involved in dehydration stress signaling in *Arabidopsis* [61]. In this study, compared with the control, the expression of almost all *TaPP2Cs*, *ABFs*, *SnRK2s* was up-regulated under DTT, whereas almost all were down-regulated after TUDCA co-treatment.

The other 208 genes were related to plant hormone biosynthesis. Among these genes, the most abundant genes were involved in SA, ET, auxin and JA biosynthesis. For example, JA is an important signaling molecule and is an endogenous regulator in plant defense against environmental stresses. In rice, the 12-oxo-phytodienoic acid reductase gene (*OsOPR1*) encodes 12-oxo-phytodienoate reductase, which is involved in the biosynthesis of JA, and *OsOPR1* may play a regulatory role in rice defense, stress response and reproductive development [62]. In foxtail millet, *SiOPR1* encodes a putative 12-oxophytodienoic acid reductase 1, which plays an important role in response to drought stress [63]. In this study, 9 JA biosynthesis-related genes (*12-oxophytodienoate reductase 1*, *OPR1*) were identified that participated in “alpha-linolenic acid metabolism” pathway. Furthermore, the 9 *OPR1* genes exhibited different expression patterns. For example, the relative expression of *Traes_2AS_A9F768C2B* and *Traes_2DS_A886F6C92* was down-regulated under DTT treatment but was up-regulated under DTT + TUDCA co-treatment. Additionally, the expression of *Traes_6DL_94DCF0B70* exhibited a trend of continuous up-regulation (Additional file 16: Figures S7J-L). The results showed TUDCA increased the expression of *OPR1*, which was followed by an increase in resistance of wheat to ER stress. Therefore, plant hormone-related genes might play important roles in wheat response to ER stress and could act as signal molecules.

#### Transcription factors

Based on the RNA-seq data in this study, we found the top five most abundant TF families were MYB, NAC, orphans, bHLH, and bZIP. MYB families play an important role in regulatory networks that control metabolism, development and response to environmental stresses [64]. For example, *Arabidopsis* MYB112 promotes anthocyanin formation under salinity and high light stress [65]. The orthologous gene of *AtMYB112*, *Traes_1AS_36AF74187*, was up-regulated under DTT and further up-regulated after TUDCA co-treatment, and we detected the dynamic changes of this gene and similar results were obtained over time (Additional file 15: Figure S6F); however, another MYB-related family gene, *Novel12259*, exhibited a different expression pattern under DTT treatment (Additional file 16: Figure S7M). Additionally, MYBs often combine with bHLHs in plant gene regulation under stress [66]. In plants, bHLHs regulate abiotic stress response and tolerance, and *TabHLH39* improves tolerance to drought, salt and cold stress in transgenic *Arabidopsis* [67]. In our study, compared with the control, the expression of *TabHLH39* (*Novel07753*) was down-regulated under DTT, whereas expression was up-regulated after TUDCA co-treatment (Additional file 16: Figure S7N).

In plants, NAC TFs are a large family of regulators and play vital roles in plant development and in response to environmental stresses [68]. In *Arabidopsis*, NAC062, NAC089 and NAC103 are identified as ER stress-related MTFs, playing important roles in responding to ER stress. In this study, the aforementioned MTFs were not detected, but other important NACs were identified, such as ANAC102, TaNAC6, TaNAC8 and TaNAC29. ANAC102 affects viability of *Arabidopsis* seeds after low-oxygen treatment [69], and *GmNAC6* (*Glycine max NAC6*) is induced by ER stress and osmotic stress and participates in the NRP-mediated cell-death signaling pathway induced by ER stress and osmotic stress [70]. TaNAC8 functions as a transcriptional activator and is involved in resisting abiotic and biotic stresses in wheat [71], and overexpression of *TaNAC29* in plants increases tolerance to high salinity and dehydration [72]. Therefore, these NAC genes may play important roles in wheat response to ER stress. Compared with the control, almost all of these TF genes were similarly up-regulated under DTT and down-regulated after TUDCA co-treatment.

bZIP TFs regulate processes that include pathogen defense, light and stress signaling, seed maturation and flower development [73]. In *Arabidopsis*, bZIP60, bZIP28 and bZIP17 are also MTFs. For example, bZIP60 plays an important role in ER stress responses in *Arabidopsis* through the up-regulation of genes encoding factors that aid in protein folding and degradation [24, 25]. In our study, compared with the control, the orthologous genes of *AtbZIP60* (*Traes_7AL_25850F96F*, *Traes_7BL_625F55A12* and *Traes_7DL_3CE000E38*) were all up-regulated under DTT and were further up-regulated after TUDCA co-treatment.

WRKYs are also widely involved in biotic and abiotic stress responses [74]. For example, WRKY33 is a TF that plays an important role in plant defense against environmental stresses. In *Arabidopsis*, WRKY33 is vital for plant resistance to necrotrophic pathogens [75], and *WRKY33* is also an autophagy regulatory gene, which is up-regulated by ER stress [28]. WRKY33 participates in heat tolerance in *Arabidopsis* [76], and overexpression of *AtWRKY33* increases salt stress tolerance in *Arabidopsis* [77]. In our study, compared with the control, the expression of *Novel13869* (*AtWRKY33/TaWRKY27*) was down-regulated under DTT but up-regulated by TUDCA co-treatment (Additional file 16: Figure S7O).

#### Other-related genes

In addition to the potential candidate genes mentioned above, we screened another 10 genes with fold changes greater than 4, such as MLO, TPP, P5CS and SRG1 (Additional file 17: Table S10).

For example, MLO protein, which is a calmodulin-binding protein (CBP), is involved in biotic and abiotic stress responses of plants [78]. *Mlo* is a key gene for resistance to powdery mildew in barley, and the wild-type gene has a negative regulatory function in plant defense, whereas *mlo* mutants show greatly increased resistance [78, 79]. Additionally, *mlo* mutants exhibited spontaneous mesophyll cell death, indicating *Mlo* likely has a functional role in cell death protection during environmental stresses [78]. We speculate that the MLO protein may play a part in inhibiting the progress of cell death; thus, the cell death ratio was reduced.

Trehalose 6-phosphate phosphatase (TPP) is involved in trehalose biosynthesis during chilling stress in rice [80], and overexpression of *OsTPP1* confers stress tolerance in rice [81]. Moreover, yeast *TPP* expressed in tobacco results in drought tolerance [82].

Delta-1-pyrroline-5-carboxylate synthetase (P5CS) is a bifunctional enzyme involved in proline biosynthesis [83]. Proline is accumulated by overexpressed *P5CS*, which confers salt tolerance in transgenic potato [84] and water and salt tolerance in transgenic rice [85].

Additionally, the other genes may also play indispensable roles in ER stress response in wheat. For example, plant SRG1 is a senescence-related gene 1 and a member of the Fe (II) / ascorbate oxidase superfamily [86], which plays an important role in anti-oxidative stress [87], and pectin lyase-like is a superfamily protein that is related to cell wall degradation and fruit softening [88].

### Comprehensive analysis of possible mechanism of ER stress regulation in wheat

To further understand ER stress regulation in wheat, other two wheat genotypes with contrasting tolerance to PEG stress, Hanxuan10 and Zhengyin1 which were used in our previous study [89], were utilized to observe the changes of their morphological, physiological and molecular index. The treatments were performed on Hanxuan10 and Zhengyin1 seedlings as that on Yunong211. And the gene expressions were monitored by qRT-PCR at 4 h, 1 d, 2 d and 4 d after treatments. Similar results were observed in Hanxuan10 and Zhengyin1 as that in Yunong211 under DTT treatment and DTT + TUDCA co-treatment (Additional files 18, 19, 20: Figures S8-S10).

Although the chemical chaperone TUDCA has been widely employed to ease ER stress in mammals and *Arabidopsis*, we conducted a set of experiments to evaluate the effects of TUDCA on wheat normal growth. We found no obvious side effects of a single TUDCA treatment, based on molecular, cellular, physiological and morphological changes (Additional files 21, 22, 23, 24: Figures S11–14). We also studied the effects of TUDCA on seedling growth of Hanxuan10 and Zhengyin1 and we observed a similar result as that of Yunong211 (Additional file 20: Figures S10 and Additional file 25-26: Figures S15-S16). In our previous study, we have reported TUDCA could alleviate osmotic stress induced cell death in which ER stress related genes were involved [37]. And we also revealed that foliar spraying 100 μg/mL of TUDCA solution had no obvious effect on seeding growth except for slightly improving the physiological characteristics of wheat leaves and enhancing the expression of *TabZIP60* under normal growing conditions. And in this study, we didn’t observe obvious side effects of TUDCA on seedling growth either except for slightly reducing root length (at 2 day), and increasing fresh weight (at 3 d and 4 d) and dry weight (at 2 d, 3 d and 4 d). Interestingly, after analyzing the RNA-seq and qPCR data using samples collected from 4 groups (Control, single DTT treated, single TUDCA treated and DTT and TUDCA co-treated), we did find several genes responded to single TUDCA treatment. We speculated the function of these genes might be a possible reason to explain the slight changes of seedling when they were treated with TUDCA only but the solid evidence is lack at present. Therefore, we need to further study these genes to understand their functions on regulation plant growth under ER stress.

**Fig. 14:**
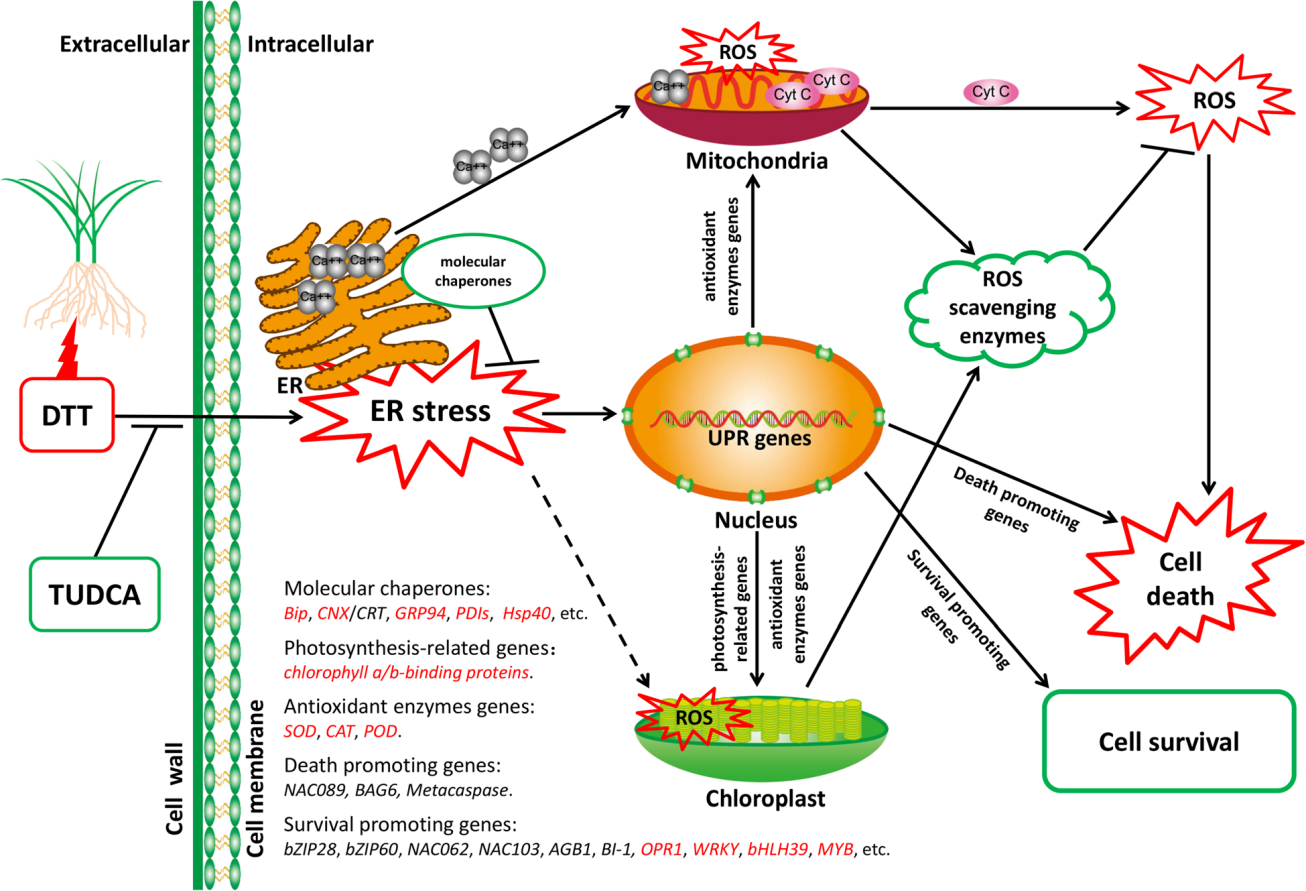
A hypothetical model for wheat response to ER stress

As discussed earlier, we analyzed the potential candidate genes conferring wheat ER stress resistance. To confirm whether these genes really respond to ER stress, we randomly chose several genes to detect their expression under various treatments (control, 20% PEG, 20% PEG+TUDCA, 42 °C heat, 42 °C heat+TUDCA, TUDCA), time courses (4 h, 1 d, 2 d and 4 d) and wheat cultivars (Yunong211, Hanxuan10 and Zhengyin1) (Additional file 27: Figure S17). Combined the changes of these genes under DTT treated and DTT + TUDCA co-treated, *chlorophyll a/b-binding protein*, *SOD* and *bHLH39* are conserved across species. Here, based on the response of potential candidate genes to ER stress, we develop a hypothetical model to elucidate a possible mechanism of wheat response to ER stress (Fig. 14).

In this study, we used DTT and TUDCA to induce or suppress ER stress, respectively. When plants are subjected to ER stress, ROS are induced. Under ER stress, calcium homeostasis is imbalanced and calcium ions are released from the ER into mitochondria. Subsequently, calcium accumulation in the mitochondria leads to the release of Cyt C and the decrease of Cyt C oxidase activity, and finally, ROS accumulate and cell death is induced [18, 32]. To protect against the damage of ROS, plant cells and its organelles such as mitochondria and chloroplasts employ antioxidant defense systems to scavenge the ROS. The cells also initiate UPR and regulate ER stress responsive genes, such as molecular chaperones, to help correct folding of proteins in the ER. In *Arabidopsis,* the cells regulate genes related to survival, such as *bZIP28* [21–23], *bZIP60* [24, 25], *NAC103* [26], *NAC062* [27], *AGB1* [90] and *BI-1* [10] in response to ER stress. However, the ability of plants to suppress ER stress is limited. Therefore, when ER stress is too severe or chronic, the cells will initiate genes associated with death, such as *NAC089* [28], *BAG6* [91] and *Metacaspase* [92], with subsequent induction of cell death. Here, based on aforementioned analyses, we provide some new insights about potential candidate genes that are involved in ER stress responses for cell survival in wheat. We conclude that genes related to molecular chaperones, photosynthesis, antioxidant enzymes, plant hormones, TFs, and others may play vital roles in responding to ER stress and promoting cell survival.

## Conclusions

Based on the collective results of the present study, the important genes involved in ER stress responses were identified and analyzed comprehensively to determine a possible mechanism of ER stress regulation in plants. Therefore, with the powerful aid of transcriptome sequence data, our understanding of the molecular mechanisms governing plant ER stress signaling will continue to increase. Furthermore, this work should provide a foundation for extended research on crop plants to improve their tolerance to environmental stresses.

## Additional files

Additional file 1: **Figure S1.** Dose effects of DTT on the growth of wheat seedlings. (A) Whole view of wheat seedlings after 6-day’s treatment. (B, C) Dynamic changes of seedling height and root length. (D) Dynamic changes of chlorophyll content. Different letters indicate significant difference among treatments at the 0.05 significance level based on Duncan’s multiple range tests. Bars represent the mean ± SD (*n* = 3). (TIF 7298 kb)

Additional file 2: **Figure S2.** Effect of DTT + TUDCA co-treatment on chlorophyll content and electrolyte leakage rate. (A) Dynamic changes of seedling chlorophyll content under various DTT + TUDCA concentrations. (B) Electrolyte leakage rate at 6-day under various treatments. Different letters indicate significant difference among treatments at the 0.05 significance level based on Duncan’s multiple range tests. Bars represent the mean ± SD (*n* = 3). (TIF 408 kb)

Additional file 3: **Table S1.** Quality summary of transcriptome data. (DOCX 14 kb)

Additional file 4: **Table S2.** List of primers used for qRT-PCR. (DOCX 16 kb)

Additional file 5: **Figure S3.** Dynamic changes of several physiological and biochemical parameters under different treatment. (A) chlorophyll a content. (B) chlorophyll b content. (C) Electrolyte leakage rate. (D) CAT activity. Different letters indicate significant difference among treatments at the 0.05 significance level based on Duncan’s multiple range tests. Bars represent the mean ± SD (*n* = 3). (TIF 724 kb)

Additional file 6: **Table S3.** Statistics of genes in different expression level intervals. (DOCX 13 kb)

Additional file 7: **Table S4.** GO enrichment of DEGs under group “D vs. C”. (XLSX 1113 kb)

Additional file 8: **Figure S4.** Enriched GO terms under group “D vs. C” displayed by directed acyclic graph (DAG). (A) Biological process; (B) Cellular component; (C) Molecular function. (TIF 1716 kb)

Additional file 9: **Table S5.** DEGs relevant to the “protein processing in endoplasmic reticulum” pathway under group “D vs. C”. (DOCX 17 kb)

Additional file 10: **Table S6.** DEGs relevant to the “plant hormone signal transduction” pathway under group “D vs. C”. (DOCX 18 kb)

Additional file 11: **Table S7.** GO enrichment of DEGs under group “T vs. D”. (XLSX 104 kb)

Additional file 12: **Figure S5.** Enriched GO terms under group “T vs. D” displayed by directed acyclic graph (DAG). (A) Biological process; (B) Molecular function. (TIF 717 kb)

Additional file 13: **Table S8.** Prediction of wheat transcription factors. (XLSX 27 kb)

Additional file 14: **Table S9.** The FPKM of DEGs related to the “protein processing in endoplasmic reticulum” pathway. (DOCX 17 kb)

Additional file 15: **Figure S6.** Several genes relative expression at four time points under different treatments. (A) *Bip1*. (B) *Bip2*. (C) *GRP94*. (D) *PDI2*. (E) *SOD*. (F) *MYB family protein*. *β-actin* was used as the internal control. The relative expression of control was normalized to 1 and Y-axis indicated the expression of each gene under DTT or DTT + TUDCA treatment relative to control by the value of 2^-ΔΔCt^. Bars represent the mean ± SD (*n* = 3). (TIF 447 kb)

Additional file 16: **Figure S7.** Several genes relative expression at 48 h under three treatments. (A) *CNX*. (B) *PDIL-1*. (C) *PDIL-5*. (D-F) *chlorophyll a/b-binding proteins*. (G) *CAT*. (H, I) *PODs*. (J-L) *OPR1s*. (M) *MYB_related family protein*. (N) *bHLH39*. (O) *WRKY family protein*. *β-actin* was used as the internal control. The relative expression of control was normalized to 1 and Y-axis indicated the expression of each gene under DTT or DTT + TUDCA treatment relative to control by the value of 2^-ΔΔCt^. Bars represent the mean ± SD (*n* = 3). (TIF 1108 kb)

Additional file 17: **Table S10.** The FPKM and annotation of DEGs that may be served as potential candidate genes. (DOCX 14 kb)

Additional file 18: **Figure S8.** Morphological changes of wheat seedlings (Hanxuan10 and Zhengyin1) after two days’ treatment. (A, D) Whole view of wheat seedlings. (B, E) Seedling height. (C, F) Root length. Different letters indicate significant difference among treatments at the 0.05 significance level based on Duncan’s multiple range tests. Bars represent the mean ± SD (*n* = 3). (TIF 4755 kb)

Additional file 19: **Figure S9.** Changes of several physiological and biochemical index under different treatments at 2-day. (A, F) Chlorophyll a content. (B, G) Chlorophyll b content. (C, H) Electrolyte leakage rate. (D, I) SOD activity. (E, J) CAT activity. Different letters indicate significant difference among treatments at the 0.05 significance level based on Duncan’s multiple range tests. Bars represent the mean ± SD (*n* = 3). (TIF 781 kb)

Additional file 20: **Figure S10.** Expression of several candidate genes under different treatments at four time points. (A, G) *Bip1*. (B, H) *chlorophyll a/b-binding protein*. (C, I) *SOD*. (D, J) *OPR1*. (E, K) *MYB family protein*. (F, L) *bHLH39*. *β-actin* was used as the internal control. The relative expression of control was normalized to 1 and Y-axis indicated the expression of each gene under DTT or DTT + TUDCA treatment relative to control by the value of 2^-ΔΔCt^. Bars represent the mean ± SD (n = 3). (TIF 1014 kb)

Additional file 21: **Figure S11.** Morphological changes of wheat seedlings under TUDCA treatment after different days. (A) Whole view of wheat seedlings after 2-day’s treatment. (B, C) Seedling height and root length. (D, E) Fresh weight and dry weight. Different letters indicate significant difference among treatments at the 0.05 significance level based on Duncan’s multiple range tests. Bars represent the mean ± SD (n = 3) (TIF 3492 kb)

Additional file 22: **Figure S12.** Physiological and biochemical changes under TUDCA treatment after different days. (A) Chlorophyll a content. (B) Chlorophyll b content. (C) Electrolyte leakage rate. (D) Water content. (E) SOD activity. (F) CAT activity. Different letters indicate significant difference among treatments at the 0.05 significance level based on Duncan’s multiple range tests. Bars represent the mean ± SD (n = 3). (TIF 832 kb)

Additional file 23: **Figure S13.** Comparison of wheat leaf and root under TUDCA treatment by trypan blue staining. (A) Trypan blue staining in leaf after 4-day’s treatment under microscope (X4). (B) Cell death ratio of leaf after 4-day’s treatment. (C, D) Trypan blue staining in seedling root after 1-day’s treatment under digital camera (C) Root system; (D) Root tip. (E) Root tip under microscope (X10). Bar = 500 μm in A and bar = 200 μm in E. Different letters of B indicate significant difference among treatments at the 0.05 significance level based on Duncan’s multiple range tests. Bars represent the mean ± SD (n = 3). (TIF 4868 kb)

Additional file 24: **Figure S14.** Relative expression of several genes under TUDCA treatment. (A, B, C) *Chlorophyll a-b binding proteins*. (D, E) *SODs*. (F) *GRP94*. (G) *CNX*. (H, I) *PDIs*. *β-actin* was used as the internal control. The relative expression of control was normalized to 1 and Y-axis indicated the expression of each gene under TUDCA treatment relative to control by the value of 2^-ΔΔCt^. Bars represent the mean ± SD (n = 3). (TIF 1016 kb)

Additional file 25: **Figure S15.** Morphological changes of wheat seedlings (Hanxuan10 and Zhengyin1) under TUDCA treatment at 2-day. (A, D) Whole view of wheat seedlings. (B, E) Seedling height. (C, F) Root length. Different letters indicate significant difference among treatments at the 0.05 significance level based on Duncan’s multiple range tests. Bars represent the mean ± SD (n = 3) (TIF 4160 kb)

Additional file 26: **Figure S16.** Physiological and biochemical changes under TUDCA treatment after different days. (A, F) Chlorophyll a content. (B, G) Chlorophyll b content. (C, H) Electrolyte leakage rate. (D, I) SOD activity. (E, J) CAT activity. Different letters indicate significant difference among treatments at the 0.05 significance level based on Duncan’s multiple range tests. Bars represent the mean ± SD (n = 3). (TIF 893 kb)

Additional file 27: **Figure S17.** Expression of several genes at 4 h under different treatments. (A-C) *Bip1*. (D-F) *Chlorophyll a/b-binding protein*. (G-I) *SOD*. (J-L) *OPR1*. (M-O) *MYB family protein*. (P-R) *bHLH39*. *β-actin* was used as the internal control. The relative expression of control was normalized to 1 and Y-axis indicated the expression of each gene under PEG (20% PEG) or PEG (20% PEG) + TUDCA or heat (42 °C) or heat (42 °C) + TUDCA or TUDCA treatment relative to control by the value of 2^-ΔΔCt^. Bars represent the mean ± SD (n = 3). (TIF 1382 kb)

## Abbreviations

CNX: Calnexin
CRT: Calreticulin
DEGs: Differentially expressed genes
DTT: Dithiothreitol
ER: Endoplasmic reticulum
GO: Gene ontology
KEGG: Kyoto Encyclopedia of Genes and Genomes
MTFs: Membrane-associated transcription factors
PDI: Protein disulfide-isomerase
qRT-PCR: Quantitative real-time PCR
TM: Tunicamycin
TUDCA: Tauroursodeoxycholic acid
