# Supplementary figures and images for: Transcriptome and physiological analyses for revealing genes involved in wheat response to endoplasmic reticulum stress
Source: BMC Plant Biol. 2019 May 9;19:193. doi: 10.1186/s12870-019-1798-7 (PMC6509841; doi:10.1186/s12870-019-1798-7)

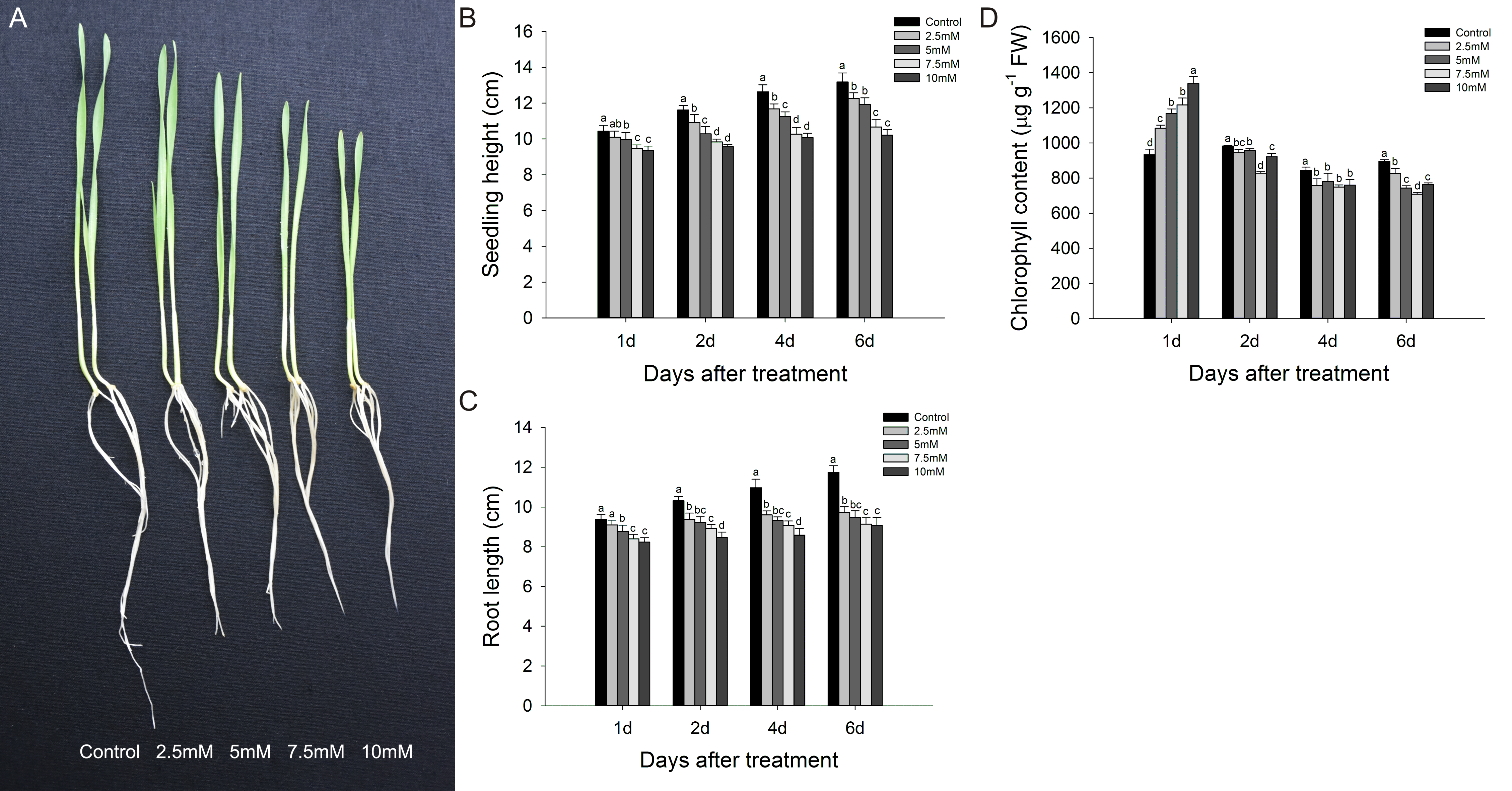

Supplement: Supplementary file 1 — Figure S1. Dose effects of DTT on the growth of wheat seedlings. (A) Whole view of wheat seedlings after 6-day’s treatment. (B, C) Dynamic changes of seedling height and root length. (D) Dynamic changes of chlorophyll content. Different letters indicate significant difference among treatments at the 0.05 significance level based on Duncan’s multiple range tests. Bars represent the mean ± SD (n = 3). (TIF 7298 kb) [file 12870_2019_1798_MOESM1_ESM.tif]

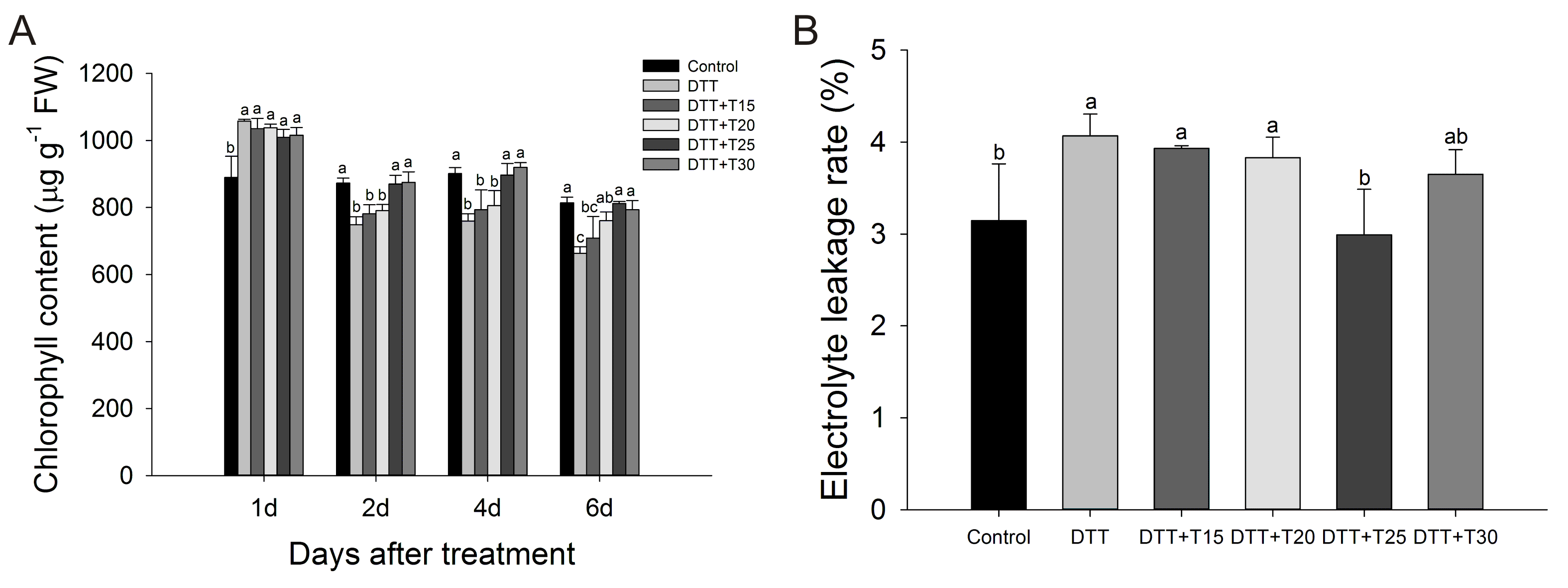

Supplement: Supplementary file 2 — Figure S2. Effect of DTT + TUDCA co-treatment on chlorophyll content and electrolyte leakage rate. (A) Dynamic changes of seedling chlorophyll content under various DTT + TUDCA concentrations. (B) Electrolyte leakage rate at 6-day under various treatments. Different letters indicate significant difference among treatments at the 0.05 significance level based on Duncan’s multiple range tests. Bars represent the mean ± SD (n = 3). (TIF 408 kb) [file 12870_2019_1798_MOESM2_ESM.tif]

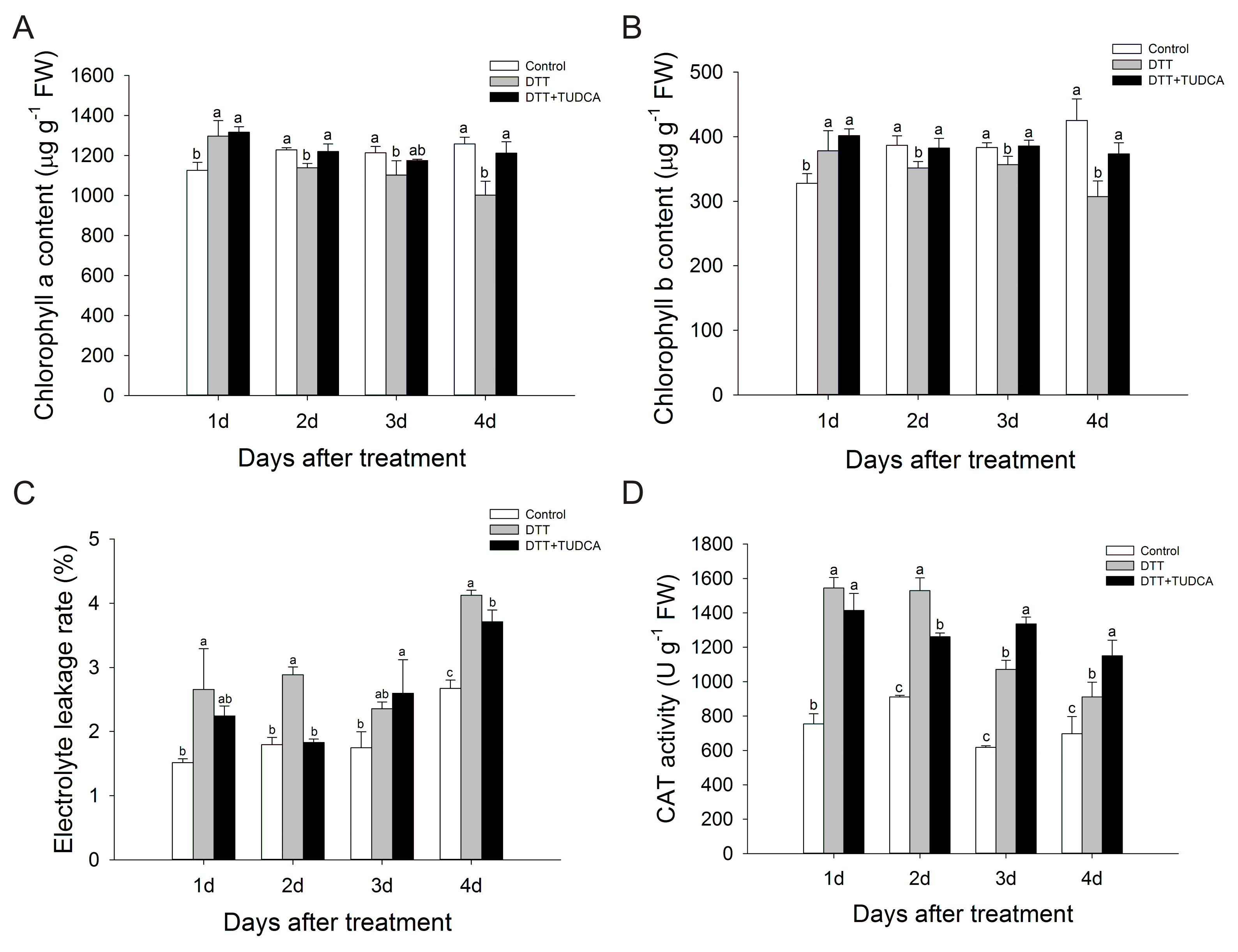

Supplement: Supplementary file 5 — Figure S3. Dynamic changes of several physiological and biochemical parameters under different treatment. (A) chlorophyll a content. (B) chlorophyll b content. (C) Electrolyte leakage rate. (D) CAT activity. Different letters indicate significant difference among treatments at the 0.05 significance level based on Duncan’s multiple range tests. Bars represent the mean ± SD (n = 3). (TIF 724 kb) [file 12870_2019_1798_MOESM5_ESM.tif]

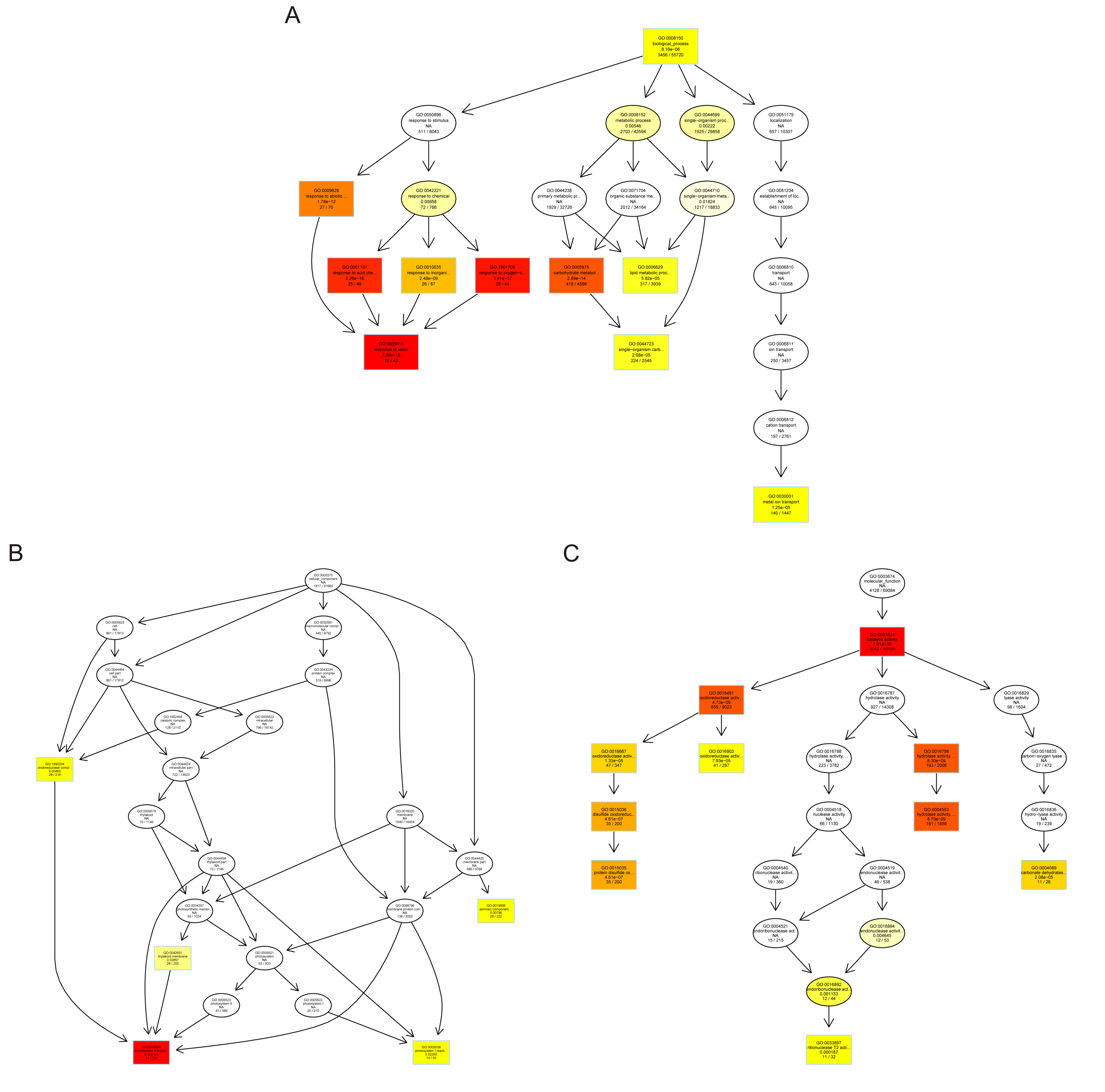

Supplement: Supplementary file 8 — Figure S4. Enriched GO terms under group “D vs. C” displayed by directed acyclic graph (DAG). (A) Biological process; (B) Cellular component; (C) Molecular function. (TIF 1716 kb) [file 12870_2019_1798_MOESM8_ESM.tif]

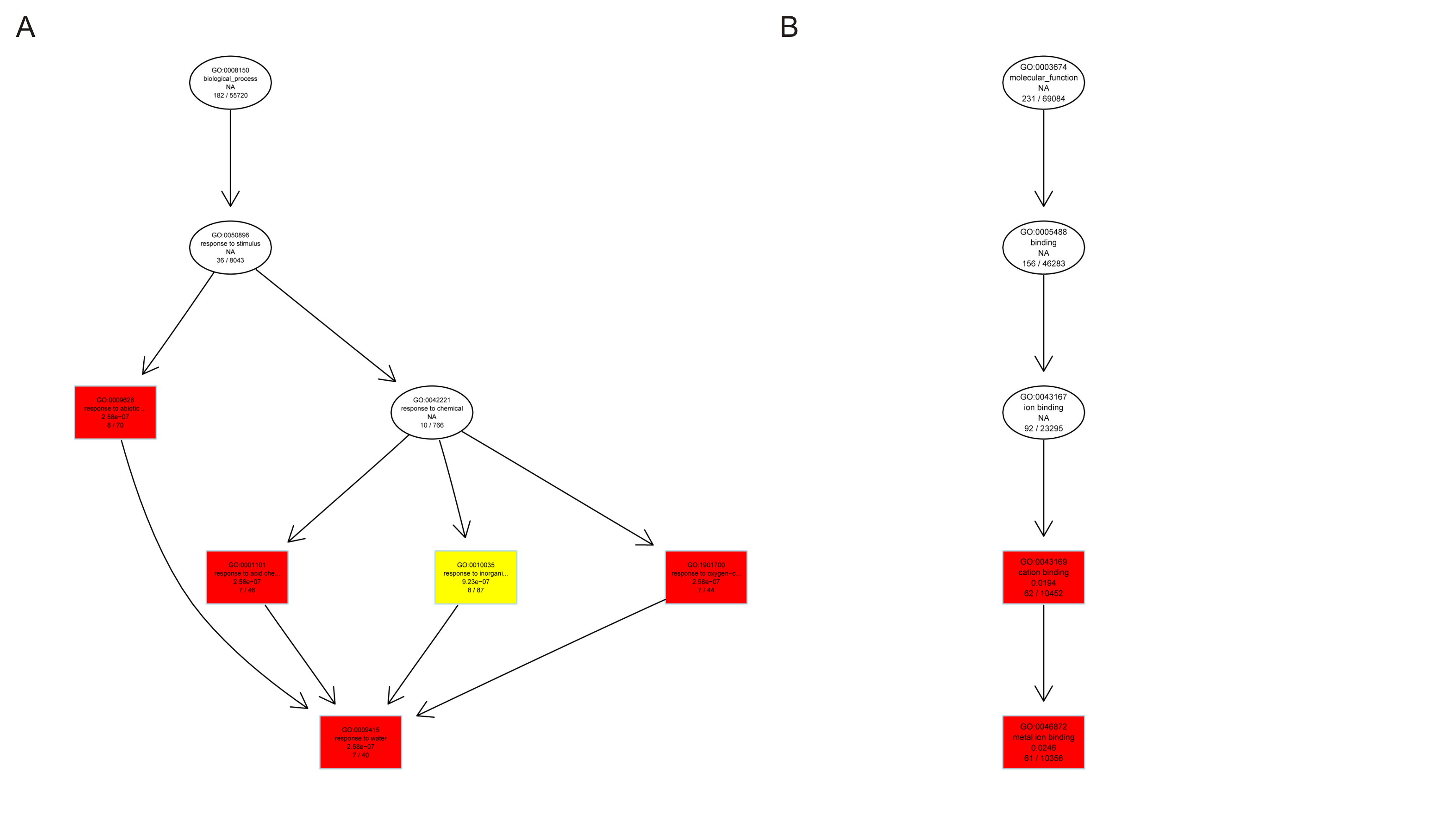

Supplement: Supplementary file 12 — Figure S5. Enriched GO terms under group “T vs. D” displayed by directed acyclic graph (DAG). (A) Biological process; (B) Molecular function. (TIF 717 kb) [file 12870_2019_1798_MOESM12_ESM.tif]

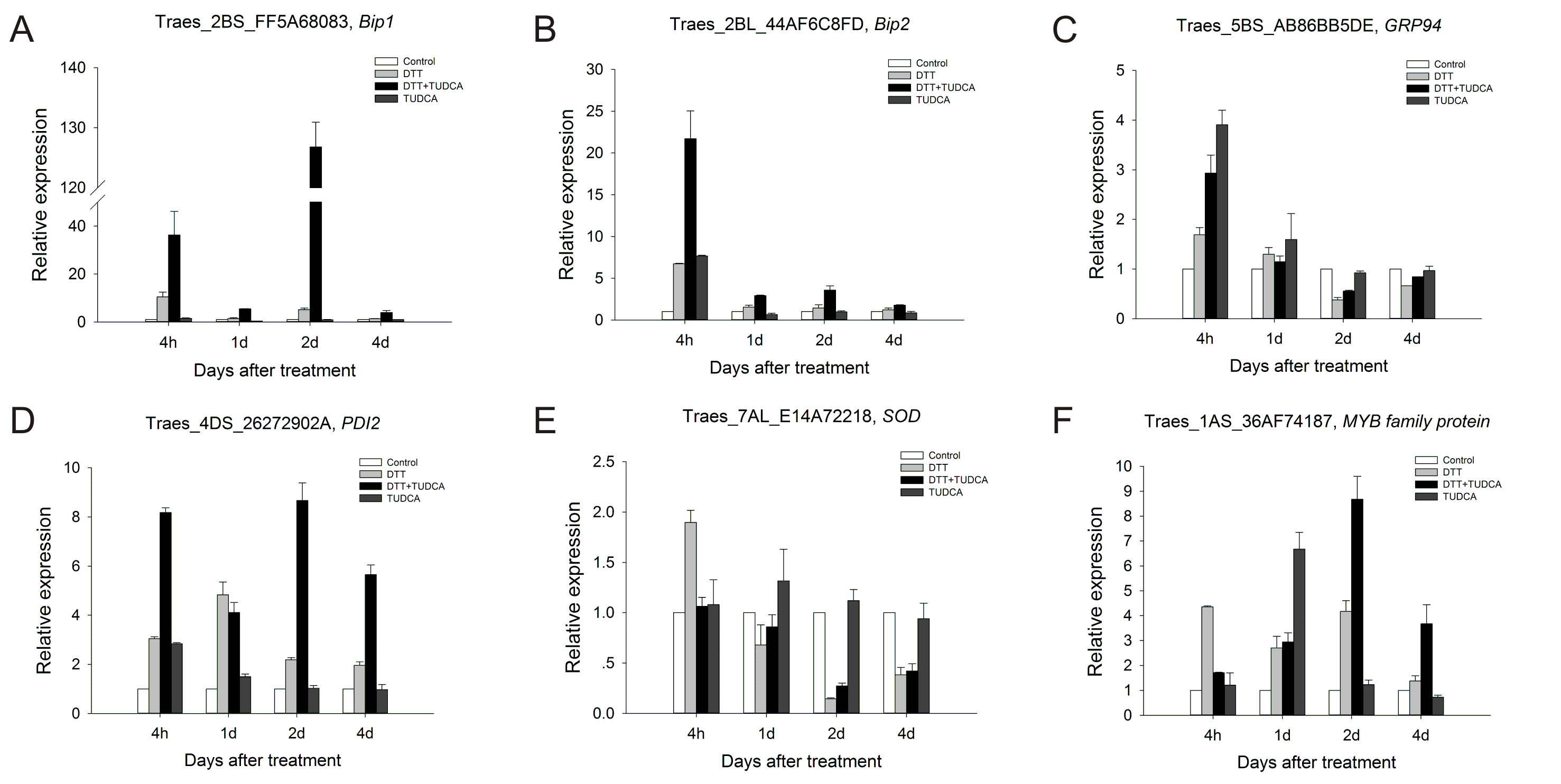

Supplement: Supplementary file 15 — Figure S6. Several genes relative expression at four time points under different treatments. (A) Bip1. (B) Bip2. (C) GRP94. (D) PDI2. (E) SOD. (F) MYB family protein. β-actin was used as the internal control. The relative expression of control was normalized to 1 and Y-axis indicated the expression of each gene under DTT or DTT + TUDCA treatment relative to control by the value of 2-ΔΔCt. Bars represent the mean ± SD (n = 3). (TIF 447 kb) [file 12870_2019_1798_MOESM15_ESM.tif]

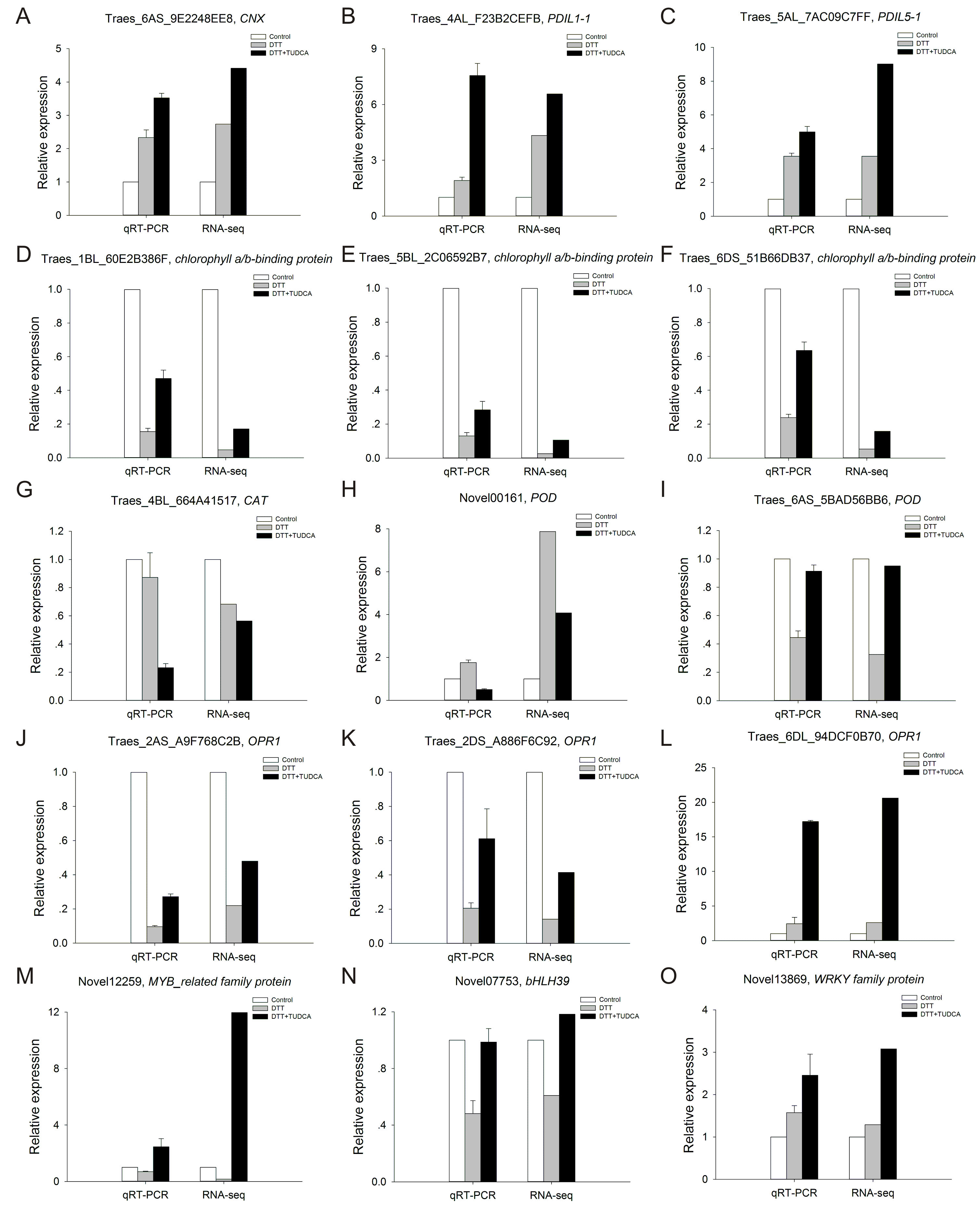

Supplement: Supplementary file 16 — Figure S7. Several genes relative expression at 48 h under three treatments. (A) CNX. (B) PDIL-1. (C) PDIL-5. (D-F) chlorophyll a/b-binding proteins. (G) CAT. (H, I) PODs. (J-L) OPR1s. (M) MYB_related family protein. (N) bHLH39. (O) WRKY family protein. β-actin was used as the internal control. The relative expression of control was normalized to 1 and Y-axis indicated the expression of each gene under DTT or DTT + TUDCA treatment relative to control by the value of 2-ΔΔCt. Bars represent the mean ± SD (n = 3). (TIF 1108 kb) [file 12870_2019_1798_MOESM16_ESM.tif]

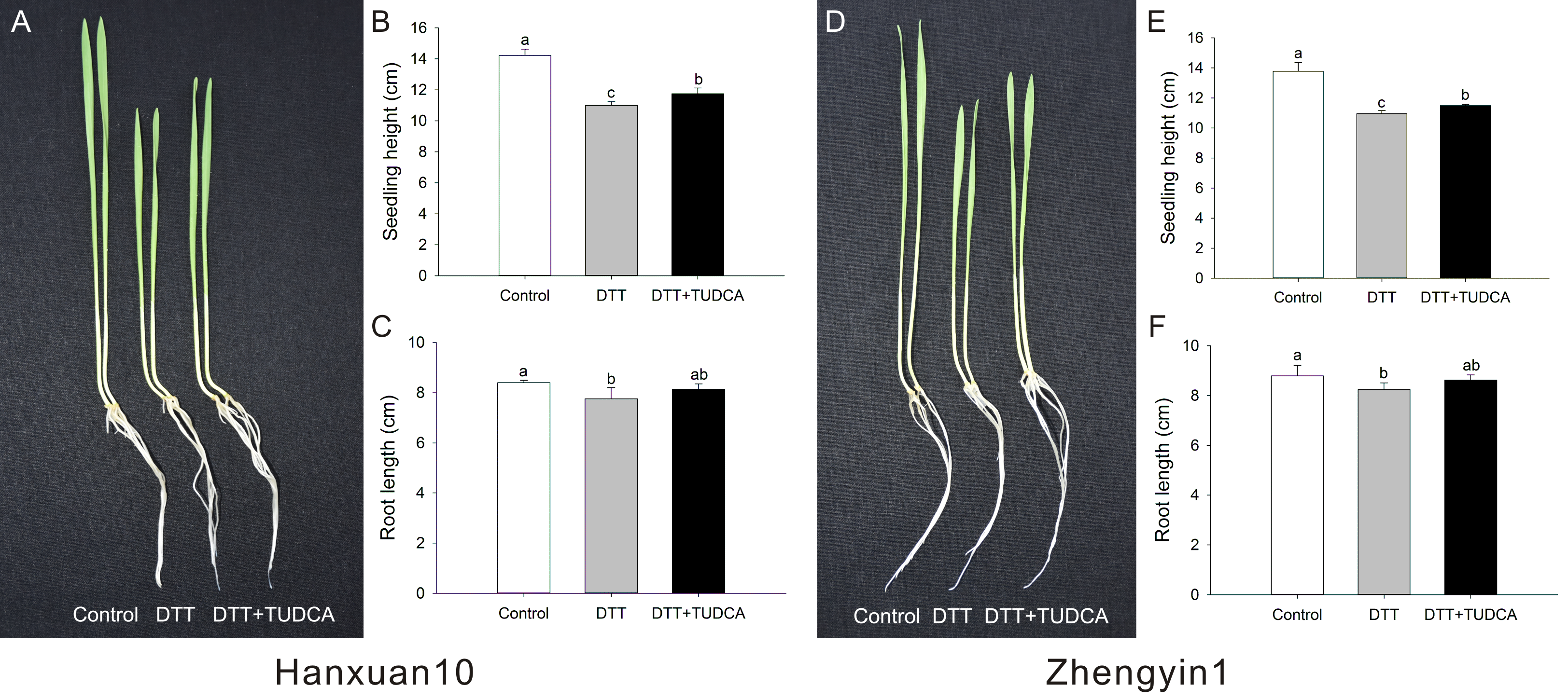

Supplement: Supplementary file 18 — Figure S8. Morphological changes of wheat seedlings (Hanxuan10 and Zhengyin1) after two days’ treatment. (A, D) Whole view of wheat seedlings. (B, E) Seedling height. (C, F) Root length. Different letters indicate significant difference among treatments at the 0.05 significance level based on Duncan’s multiple range tests. Bars represent the mean ± SD (n = 3). (TIF 4755 kb) [file 12870_2019_1798_MOESM18_ESM.tif]

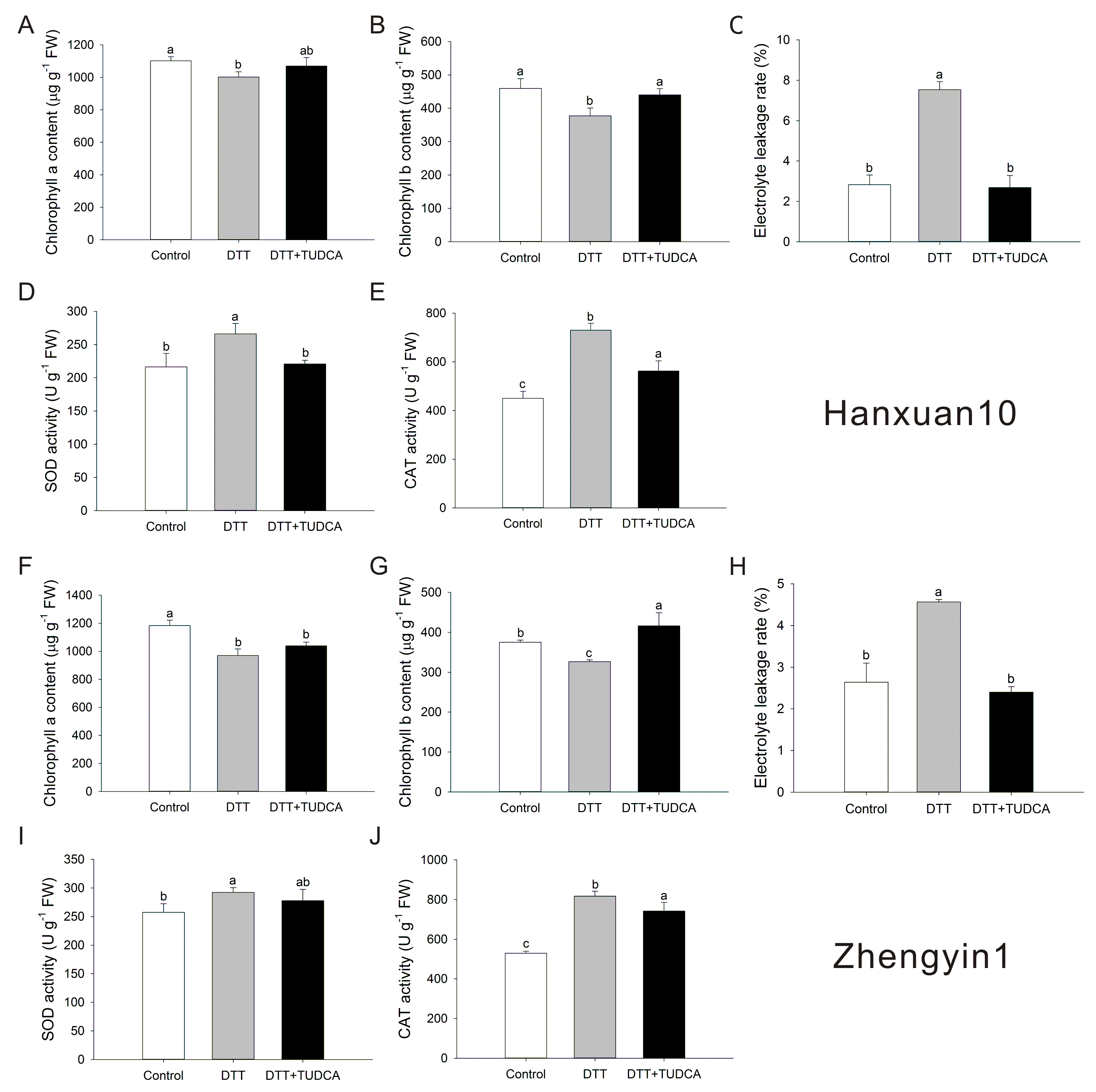

Supplement: Supplementary file 19 — Figure S9. Changes of several physiological and biochemical index under different treatments at 2-day. (A, F) Chlorophyll a content. (B, G) Chlorophyll b content. (C, H) Electrolyte leakage rate. (D, I) SOD activity. (E, J) CAT activity. Different letters indicate significant difference among treatments at the 0.05 significance level based on Duncan’s multiple range tests. Bars represent the mean ± SD (n = 3). (TIF 781 kb) [file 12870_2019_1798_MOESM19_ESM.tif]

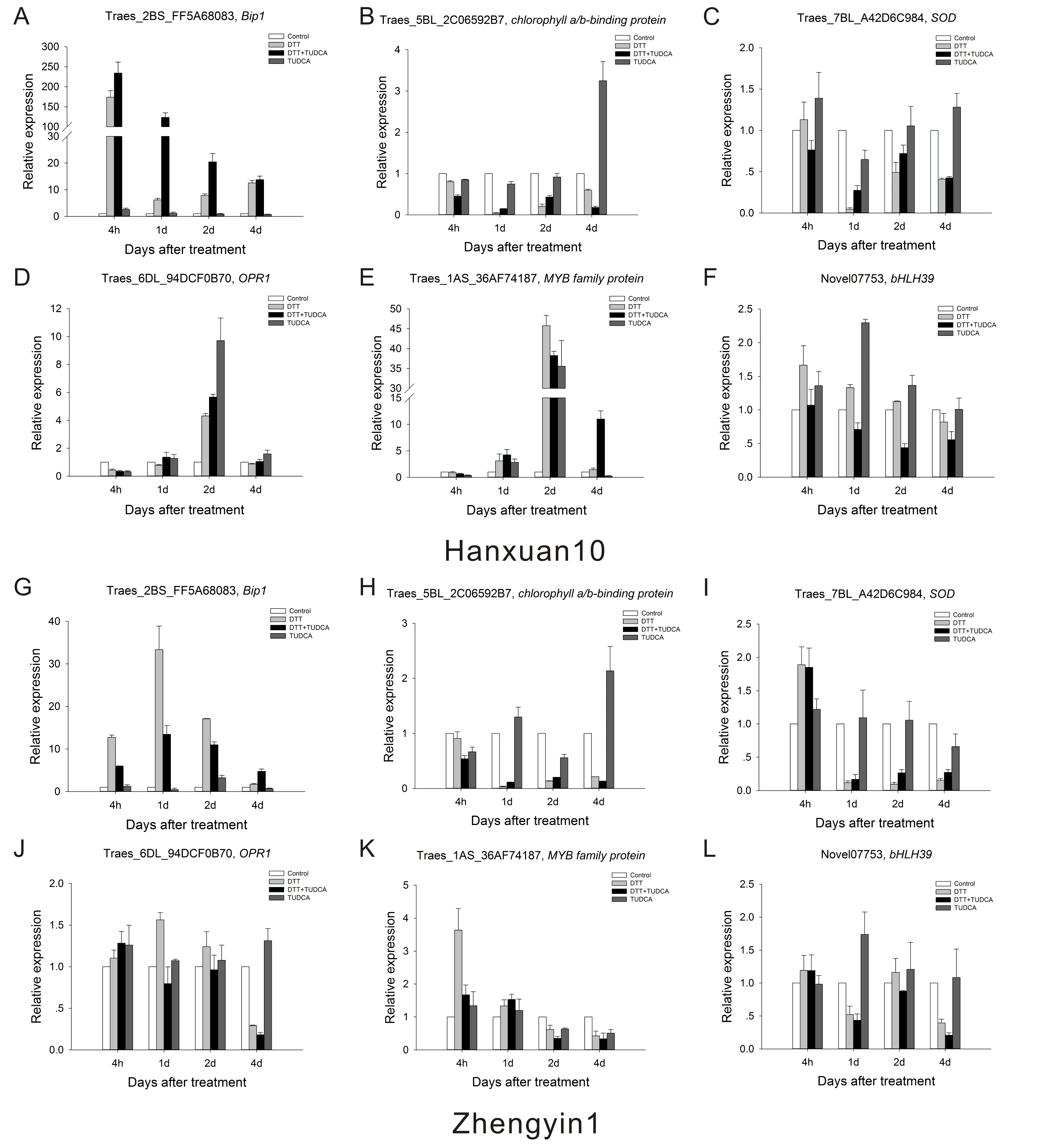

Supplement: Supplementary file 20 — Figure S10. Expression of several candidate genes under different treatments at four time points. (A, G) Bip1. (B, H) chlorophyll a/b-binding protein. (C, I) SOD. (D, J) OPR1. (E, K) MYB family protein. (F, L) bHLH39. β-actin was used as the internal control. The relative expression of control was normalized to 1 and Y-axis indicated the expression of each gene under DTT or DTT + TUDCA treatment relative to control by the value of 2-ΔΔCt. Bars represent the mean ± SD (n = 3). (TIF 1014 kb) [file 12870_2019_1798_MOESM20_ESM.tif]

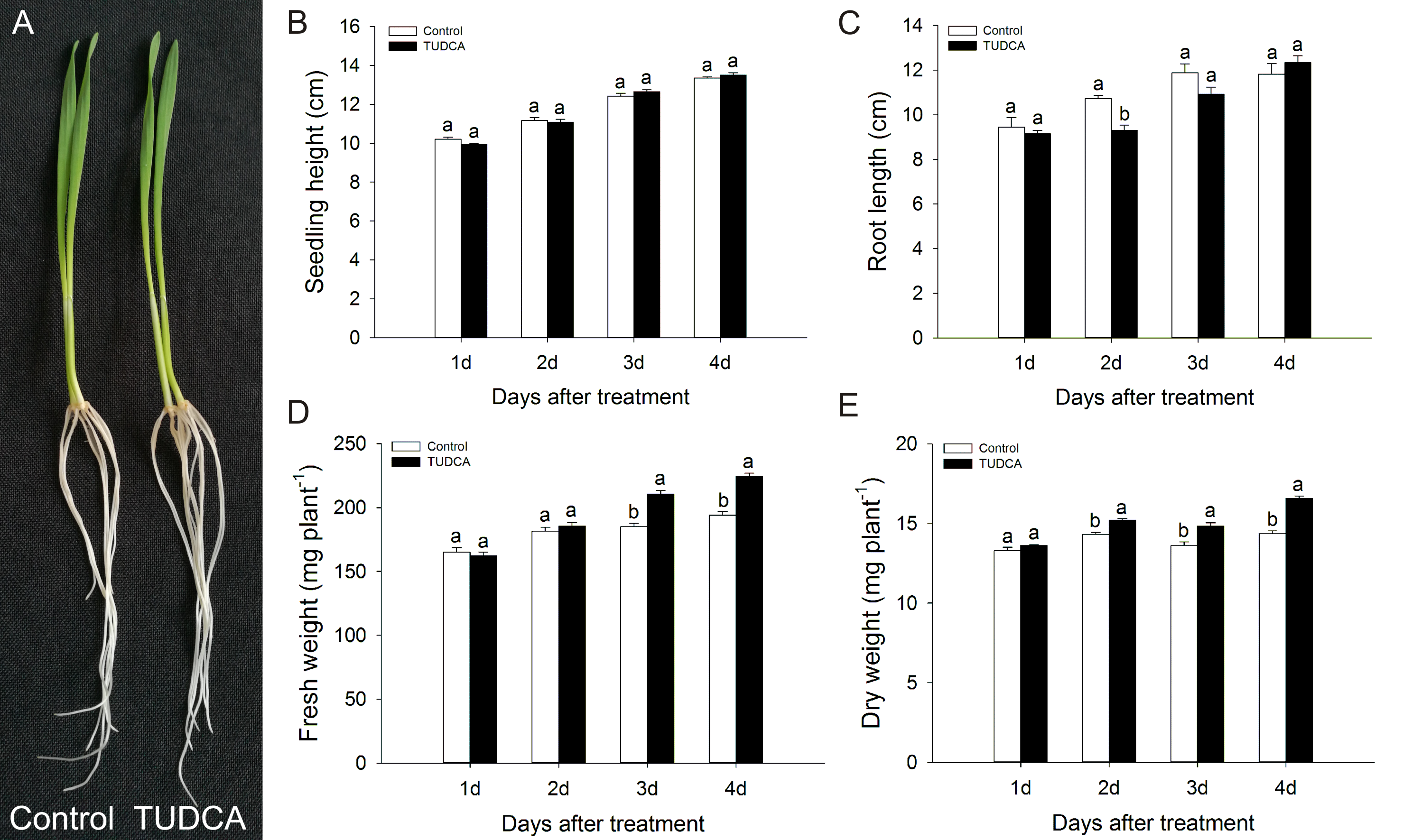

Supplement: Supplementary file 21 — Figure S11. Morphological changes of wheat seedlings under TUDCA treatment after different days. (A) Whole view of wheat seedlings after 2-day’s treatment. (B, C) Seedling height and root length. (D, E) Fresh weight and dry weight. Different letters indicate significant difference among treatments at the 0.05 significance level based on Duncan’s multiple range tests. Bars represent the mean ± SD (n = 3) (TIF 3492 kb) [file 12870_2019_1798_MOESM21_ESM.tif]

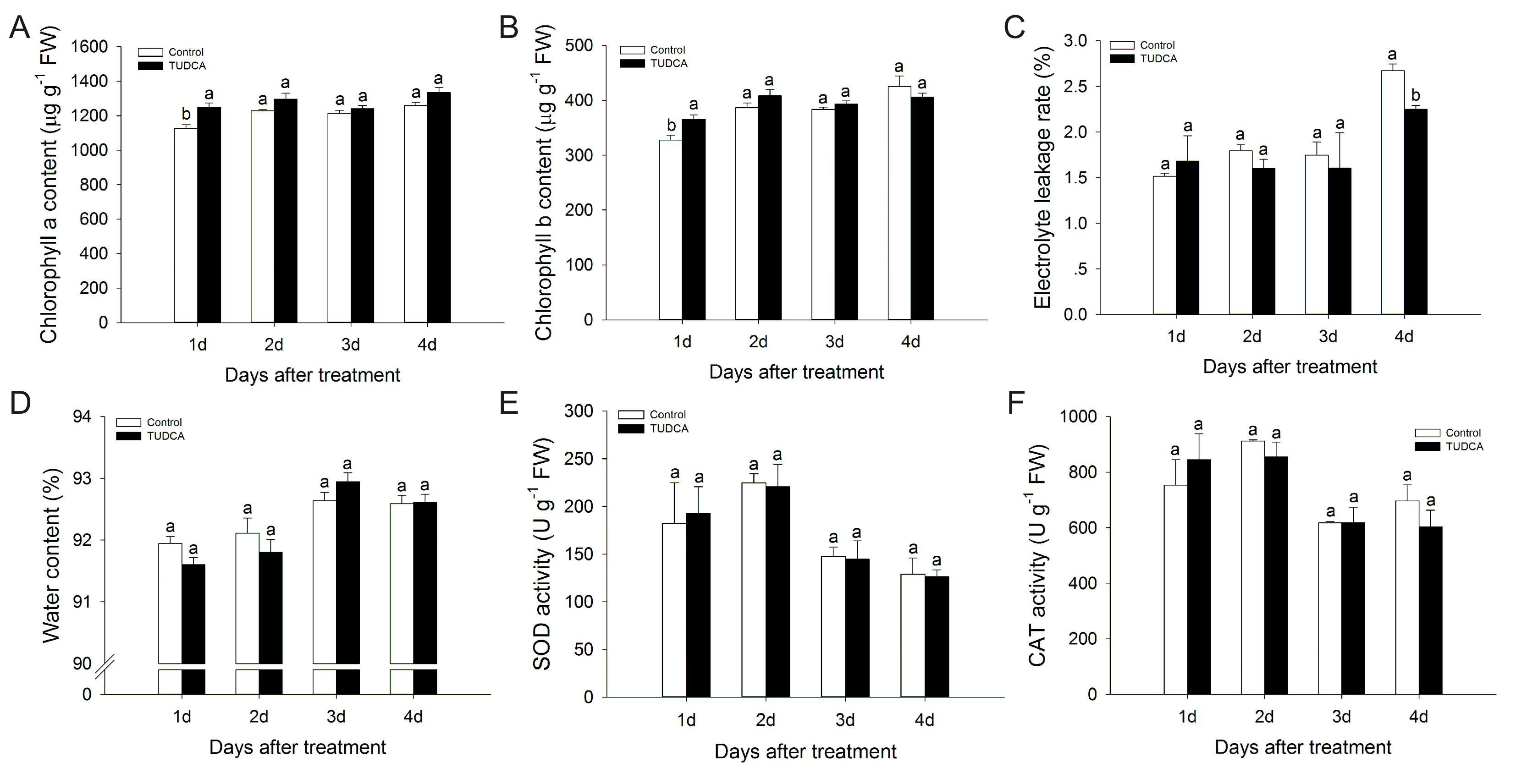

Supplement: Supplementary file 22 — Figure S12. Physiological and biochemical changes under TUDCA treatment after different days. (A) Chlorophyll a content. (B) Chlorophyll b content. (C) Electrolyte leakage rate. (D) Water content. (E) SOD activity. (F) CAT activity. Different letters indicate significant difference among treatments at the 0.05 significance level based on Duncan’s multiple range tests. Bars represent the mean ± SD (n = 3). (TIF 832 kb) [file 12870_2019_1798_MOESM22_ESM.tif]

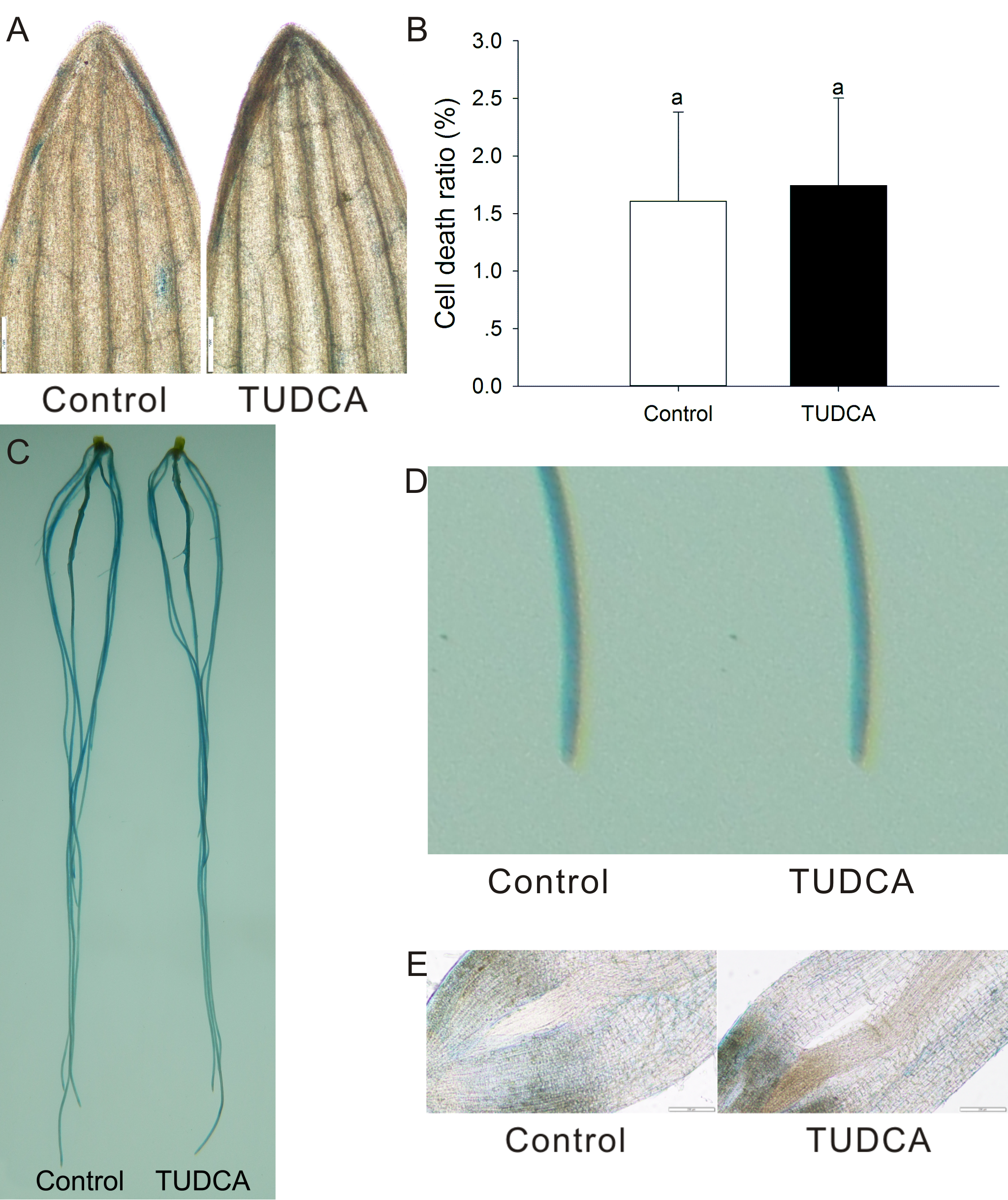

Supplement: Supplementary file 23 — Figure S13. Comparison of wheat leaf and root under TUDCA treatment by trypan blue staining. (A) Trypan blue staining in leaf after 4-day’s treatment under microscope (X4). (B) Cell death ratio of leaf after 4-day’s treatment. (C, D) Trypan blue staining in seedling root after 1-day’s treatment under digital camera (C) Root system; (D) Root tip. (E) Root tip under microscope (X10). Bar = 500 μm in A and bar = 200 μm in E. Different letters of B indicate significant difference among treatments at the 0.05 significance level based on Duncan’s multiple range tests. Bars represent the mean ± SD (n = 3). (TIF 4868 kb) [file 12870_2019_1798_MOESM23_ESM.tif]

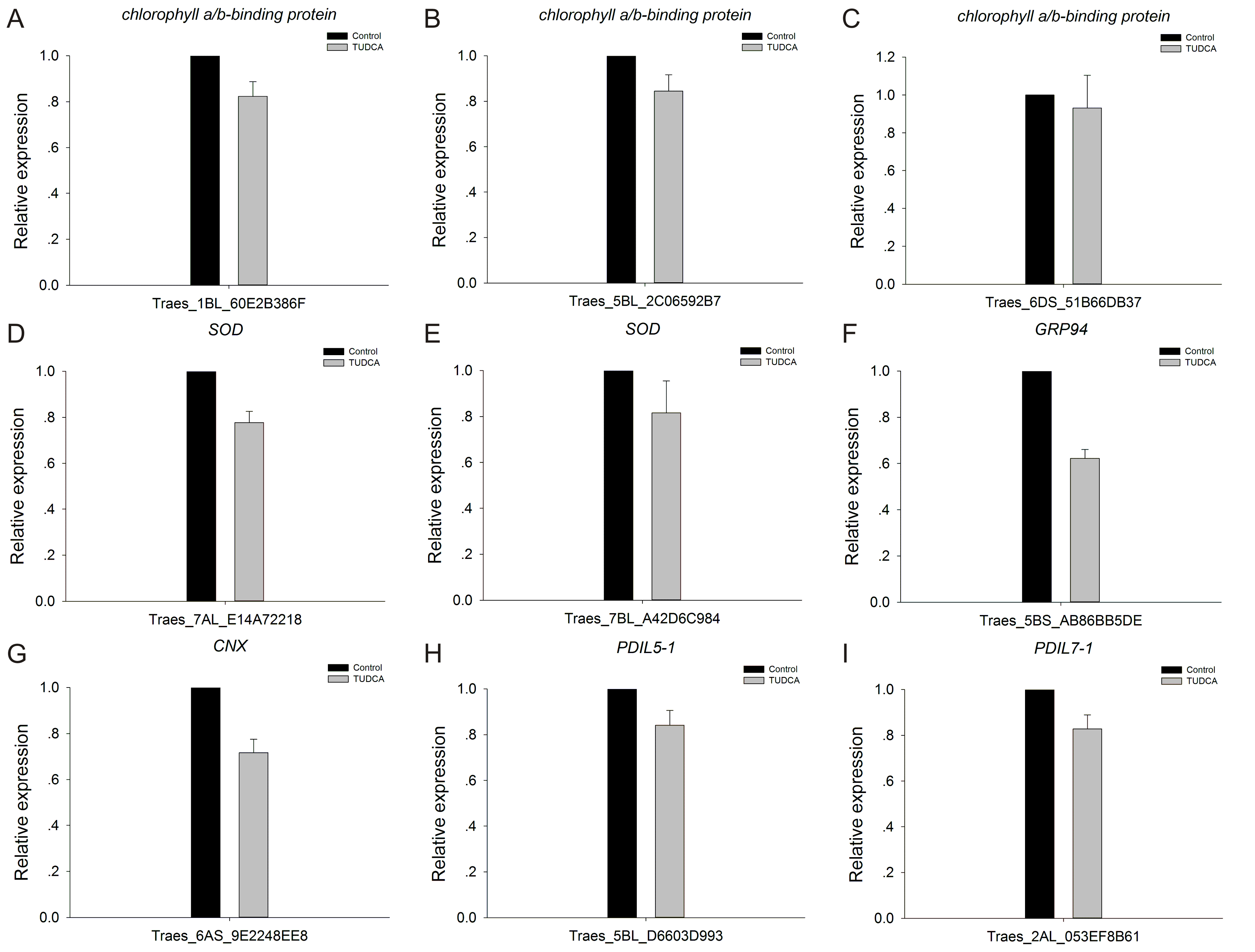

Supplement: Supplementary file 24 — Figure S14. Relative expression of several genes under TUDCA treatment. (A, B, C) Chlorophyll a-b binding proteins. (D, E) SODs. (F) GRP94. (G) CNX. (H, I) PDIs. β-actin was used as the internal control. The relative expression of control was normalized to 1 and Y-axis indicated the expression of each gene under TUDCA treatment relative to control by the value of 2-ΔΔCt. Bars represent the mean ± SD (n = 3). (TIF 1016 kb) [file 12870_2019_1798_MOESM24_ESM.tif]

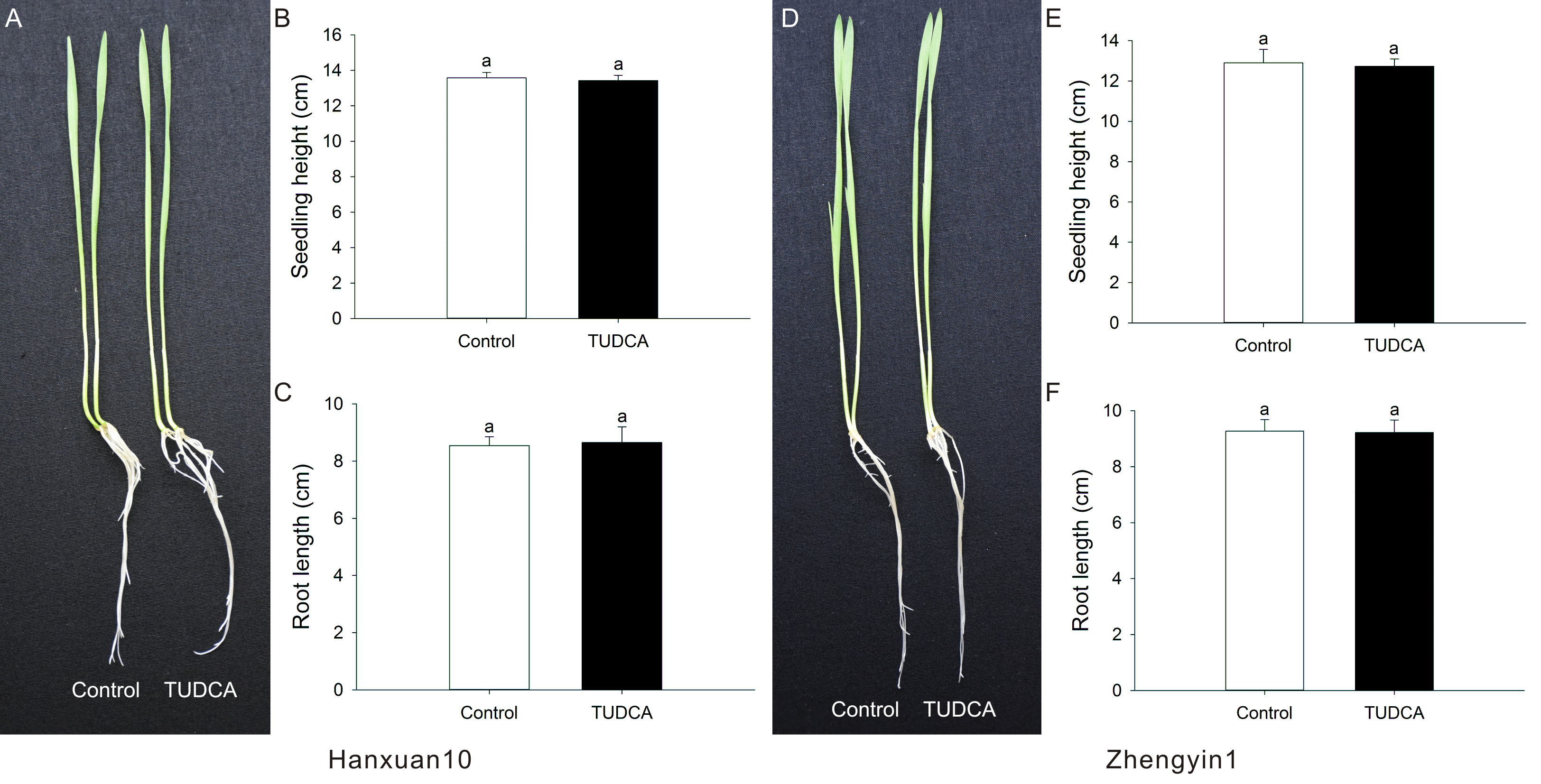

Supplement: Supplementary file 25 — Figure S15. Morphological changes of wheat seedlings (Hanxuan10 and Zhengyin1) under TUDCA treatment at 2-day. (A, D) Whole view of wheat seedlings. (B, E) Seedling height. (C, F) Root length. Different letters indicate significant difference among treatments at the 0.05 significance level based on Duncan’s multiple range tests. Bars represent the mean ± SD (n = 3) (TIF 4160 kb) [file 12870_2019_1798_MOESM25_ESM.tif]

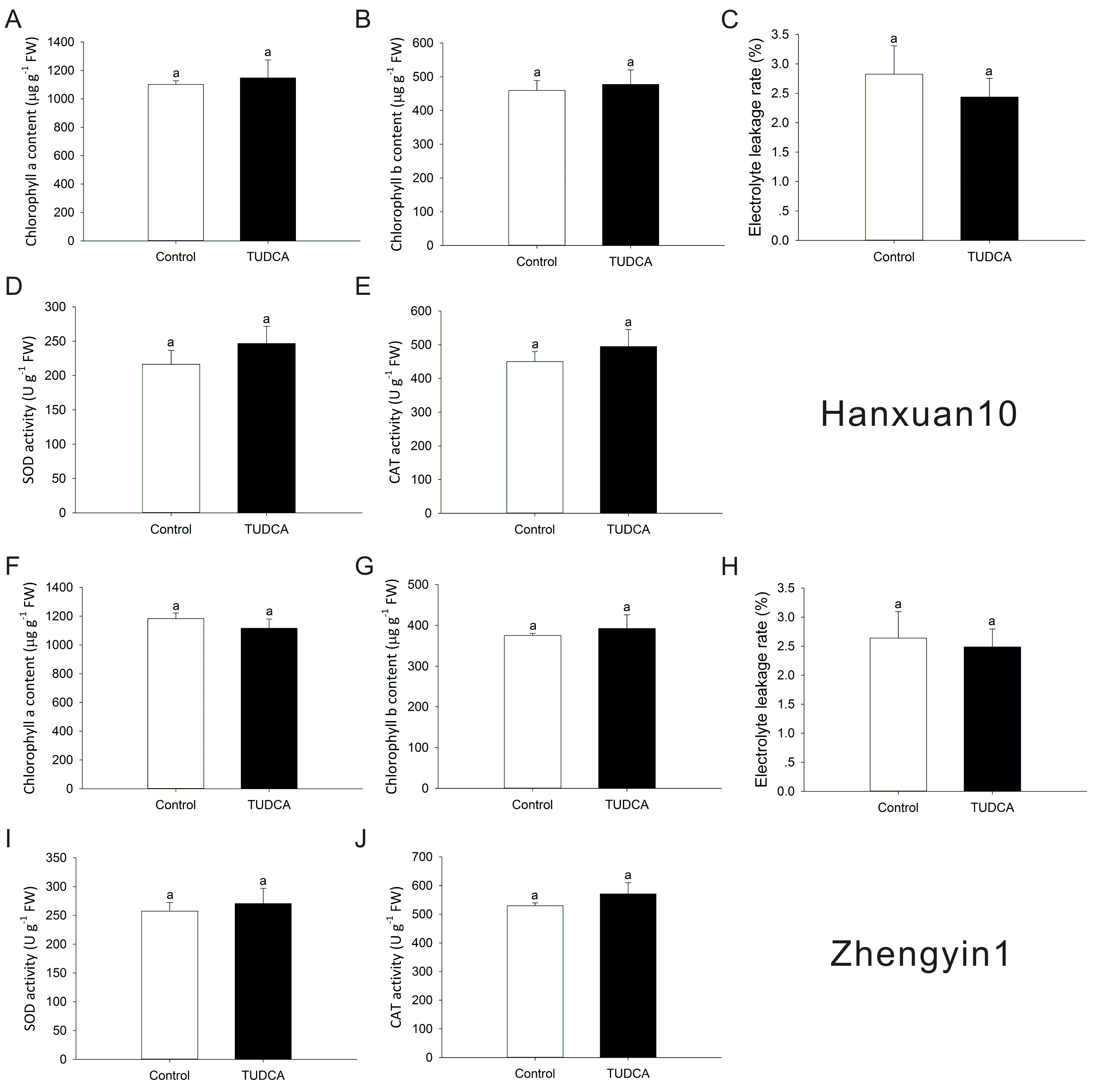

Supplement: Supplementary file 26 — Figure S16. Physiological and biochemical changes under TUDCA treatment after different days. (A, F) Chlorophyll a content. (B, G) Chlorophyll b content. (C, H) Electrolyte leakage rate. (D, I) SOD activity. (E, J) CAT activity. Different letters indicate significant difference among treatments at the 0.05 significance level based on Duncan’s multiple range tests. Bars represent the mean ± SD (n = 3). (TIF 893 kb) [file 12870_2019_1798_MOESM26_ESM.tif]

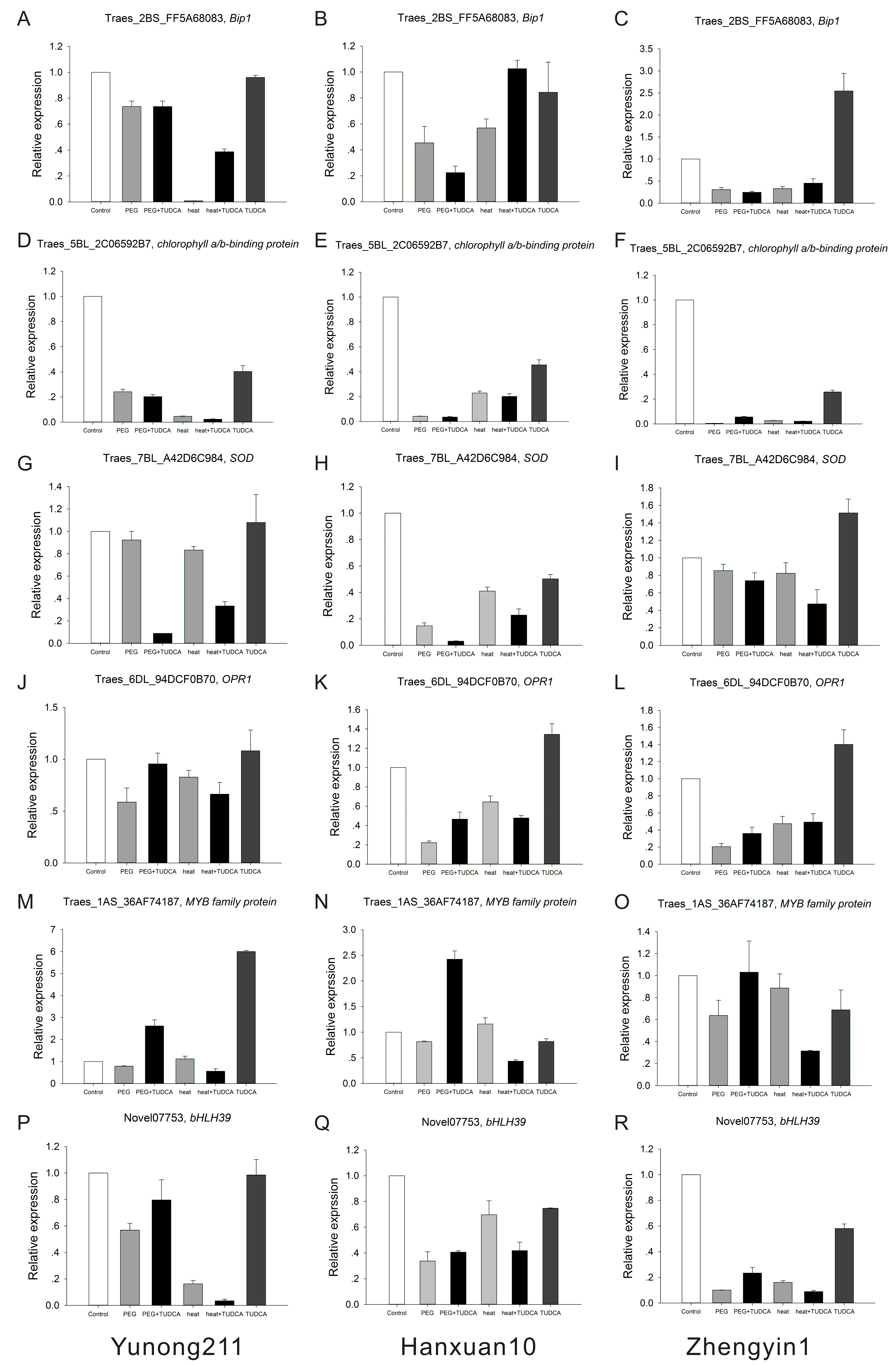

Supplement: Supplementary file 27 — Figure S17. Expression of several genes at 4 h under different treatments. (A-C) Bip1. (D-F) Chlorophyll a/b-binding protein. (G-I) SOD. (J-L) OPR1. (M-O) MYB family protein. (P-R) bHLH39. β-actin was used as the internal control. The relative expression of control was normalized to 1 and Y-axis indicated the expression of each gene under PEG (20% PEG) or PEG (20% PEG) + TUDCA or heat (42 °C) or heat (42 °C) + TUDCA or TUDCA treatment relative to control by the value of 2-ΔΔCt. Bars represent the mean ± SD (n = 3). (TIF 1382 kb) [file 12870_2019_1798_MOESM27_ESM.tif]
