# Supplementary material for: Transcriptome and physiological analyses for revealing genes involved in wheat response to endoplasmic reticulum stress
Source: BMC Plant Biol. 2019 May 9;19:193. doi: 10.1186/s12870-019-1798-7 (PMC6509841; doi:10.1186/s12870-019-1798-7)
Supplement: Supplementary file 3 — Table S1. Quality summary of transcriptome data. (DOCX 14 kb) [file 12870_2019_1798_MOESM3_ESM.docx]

| **Table S1** Quality summary of transcriptome data | | | | | | | |
| --- | --- | --- | --- | --- | --- | --- | --- |
| **Sample** | **Raw reads** | **Clean reads** | **clean bases**  **(GB)** | **Error rate**  **(%)** | **Q20(%)** | **Q30(%)** | **GC content**  **(%)** |
| **C_1** | 64492780 | 62896826 | 9.43 | 0.01 | 97.46 | 93.17 | 55.49 |
| **C_2** | 60861334 | 59066748 | 8.86 | 0.02 | 97.21 | 92.61 | 55.32 |
| **C_3** | 89246720 | 86362530 | 12.95 | 0.01 | 97.84 | 94.20 | 55.29 |
| **D_1** | 68782402 | 66772550 | 10.02 | 0.02 | 97.21 | 92.66 | 53.45 |
| **D_2** | 61842306 | 60035714 | 9.01 | 0.01 | 97.38 | 93.02 | 53.74 |
| **D_3** | 76694926 | 73485888 | 11.02 | 0.02 | 96.83 | 92.00 | 53.89 |
| **T_1** | 67551788 | 65768728 | 9.87 | 0.02 | 97.29 | 92.80 | 54.11 |
| **T_2** | 63085998 | 60385802 | 9.06 | 0.02 | 96.97 | 92.28 | 52.26 |
| **T_3** | 59563026 | 58066732 | 8.71 | 0.01 | 97.51 | 93.30 | 52.99 |
| **Total** | 612121280 | 592841518 | 88.93 | -- | -- | -- | -- |
| **Average** | 68013476 | 65871280 | 9.88 | 0.02 | 97.30 | 92.89 | 54.06 |
| Notes: Three biological replicates were used in this study and marked with 1, 2 and 3, respectively. C, control; D, DTT; T, DTT+TUDCA. | | | | | | | |
