# Supplementary material for: Transcriptome and physiological analyses for revealing genes involved in wheat response to endoplasmic reticulum stress
Source: BMC Plant Biol. 2019 May 9;19:193. doi: 10.1186/s12870-019-1798-7 (PMC6509841; doi:10.1186/s12870-019-1798-7)
Supplement: Supplementary file 4 — Table S2. List of primers used for qRT-PCR. (DOCX 16 kb) [file 12870_2019_1798_MOESM4_ESM.docx]

| **Table S2** List of primers used for qRT-PCR | | |
| --- | --- | --- |
| **Number** | **Primer name** | **Sequence (5' to 3')** |
| **1** | Novel04197-F | CGAGGCGTGGTATGGGATGT |
| **2** | Novel04197-R | GCTCCTGTCGTCGAGCTTCT |
| **3** | Traes_1AL_6E5BA9C6F-F | ATGATTATTCGTCCGATGAG |
| **4** | Traes_1AL_6E5BA9C6F-R | CAGTAATCTTCCAGACAGTC |
| **5** | Traes_1BL_60E2B386F-F | CTGCAAAGGCCAAGCAGGTT |
| **6** | Traes_1BL_60E2B386F-R | ACTCGCCGGTCAAGTAGCTC |
| **7** | Traes_1DL_18BBECFD8-F | CGTTGAAGGGTACCGCGTTG |
| **8** | Traes_1DL_18BBECFD8-R | TCACCTTGAGCTCCGCGAAT |
| **9** | Traes_2AS_6269D889E-F | GAGATCTTCGCGCTCACCGA |
| **10** | Traes_2AS_6269D889E-R | GTTGGTCCTGAAGGGCTGGT |
| **11** | Traes_3AL_0C7CB044E-F | TACATGAGCCAGCACCAGCA |
| **12** | Traes_3AL_0C7CB044E-R | GTCTCGTCGTGTCGTTTGCC |
| **13** | Traes_4BL_9421592BC-F | GGCAACAGTCCTGGAGGTGA |
| **14** | Traes_4BL_9421592BC-R | AGCACGTACACCGCGTAGAT |
| **15** | Traes_4DS_220628F6B-F | CCACGCCGGAGTACTGAGAT |
| **16** | Traes_4DS_220628F6B-R | ACTCCACGACACACACACACT |
| **17** | Traes_5BL_2C06592B7-F | GCGTTACCATGCGCAAGACT |
| **18** | Traes_5BL_2C06592B7-R | AAGGTAGAGCACGCGGTCAG |
| **19** | Traes_5BL_304FAFA26-F | GAGTTCGCCAGGGAGATGCT |
| **20** | Traes_5BL_304FAFA26-R | CTCGAGGCTCTTGCCGATGT |
| **21** | Traes_5BL_C4A77F5DB-F | ACGTTCCGGTGTGGAGATGG |
| **22** | Traes_5BL_C4A77F5DB-R | GGCGCAACATCGATCTCACTG |
| **23** | Traes_5DL_7BEC640FA-F | GGCCAACTTCCAGTCCCTGT |
| **24** | Traes_5DL_7BEC640FA-R | TCGGAGCGGTTGAACCTCAG |
| **25** | Traes_6DL_94DCF0B70-F | AATCCTCTACTGCCACAT |
| **26** | Traes_6DL_94DCF0B70-R | ATCGCTTTATCTCCATCATC |
| **27** | Traes_6DS_51B66DB37-F | GTCCCTCTCCTCCTCGACCT |
| **28** | Traes_6DS_51B66DB37-R | CCCGGTCAGCACCATACCAT |
| **29** | Traes_7BS_6606D4150-F | GCATCACAGCAACAGATA |
| **30** | Traes_7BS_6606D4150-R | CTTGAGAGCACAGAATAGAAT |
| **31** | Traes_7DS_4DEABD294-F | TTCAGCTGCCTGCTGTGGAT |
| **32** | Traes_7DS_4DEABD294-R | TGGCGATGTAGAGGCTCTCG |
| **33** | Traes_5BS_AB86BB5DE-F | GCTTGTCGTCCCTCAAGCTG |
| **34** | Traes_5BS_AB86BB5DE-R | AGGTGGCGCGTCAGATGAAT |
| **35** | Traes_6AS_9E2248EE8-F | GGCCTCGGTTATCGTCGTCA |
| **36** | Traes_6AS_9E2248EE8-R | ATCAGGTACGACGGCACTGG |
| **37** | Traes_4DS_26272902A-F | CAGCGGGAAGAAGGTCTCGT |
| **38** | Traes_4DS_26272902A-R | CTGCCCAGCAGTCTCCTTGT |
| **39** | Traes_5BL_D6603D993-F | GGTGGTGCTGGTGGAGTTCT |
| **40** | Traes_5BL_D6603D993-R | ATATTGCTGCGCGAGCTCCT |
| **41** | Traes_4AL_F23B2CEFB-F | TTGCTGAGAAGCTGCGGTCT |
| **42** | Traes_4AL_F23B2CEFB-R | CCAATGGCCTCTCCACTGCT |
| **43** | Traes_5AL_7AC09C7FF-F | CACCAGTGGATTATGAAGG |
| **44** | Traes_5AL_7AC09C7FF-R | ACCTCCTGATGTCTTACC |
| **45** | Traes_2AL_053EF8B61-F | GTCAAACAATGGGAGGAG |
| **46** | Traes_2AL_053EF8B61-R | CGCCTTCTTCTAACTTCTC |
| **47** | Traes_7AL_E14A72218-F | ACGCCACCAAGAAGGCAGT |
| **48** | Traes_7AL_E14A72218-R | CCGTCGTCTTCCTGGGTGAG |
| **49** | Traes_7BL_A42D6C984-F | ACGCCACCAAGAAGGCAGT |
| **50** | Traes_7BL_A42D6C984-R | GAACGTTCACCGTCGTGGGA |
| **51** | Traes_6AS_7FB8F9A66-F | CAGGCAGAGCAGAGAGCAGT |
| **52** | Traes_6AS_7FB8F9A66-R | TTGGTCGTCGTCGTCTTGGT |
| **53** | Traes_6DS_3522B8EF6-F | TCCGCTTCTCCACCGTCATC |
| **54** | Traes_6DS_3522B8EF6-R | GGGAAGTTGTTGCCGAGCAG |
| **55** | Novel00161-F | GGACCTGTGCGTGGACTACT |
| **56** | Novel00161-R | ATGCGAGGGTCCGACTTGTG |
| **57** | Traes_2AS_15ECF8D51-F | GCTCGAGACCATGTGCAAGC |
| **58** | Traes_2AS_15ECF8D51-R | TTTGCGTTGGTGGAGTCCCT |
| **59** | Traes_6AS_5BAD56BB6-F | CAAGAACCTGGCACCGTTCG |
| **60** | Traes_6AS_5BAD56BB6-R | AGCACCTGGTCGGAGTTGAG |
| **61** | Traes_6BS_0BDACE205-F | AAGCTAAACCCGCGTGACCT |
| **62** | Traes_6BS_0BDACE205-R | CGAATGCCGGGTCGATGTTG |
| **63** | Traes_2AS_A9F768C2B-F | CTTCATGGACTATATGGACTG |
| **64** | Traes_2AS_A9F768C2B-R | GGAGGTAGAGGATGTTGT |
| **65** | Traes_6BL_5B613F9E5-F | CCCTCCTCGCCCACTACAAG |
| **66** | Traes_6BL_5B613F9E5-R | AGGCCTCCTTTGGTTGCTCT |
| **67** | Traes_6DL_94DCF0B70-F | TCCCACAGGGTGGTTCTTGC |
| **68** | Traes_6DL_94DCF0B70-R | AGGCCTCCTTTGGTTGCTCT |
| **69** | Novel13869-F | GAGAACTCGTCGGCGTCCTT |
| **70** | Novel13869-R | TATTCCCTCGCCGTCACCAC |
| **71** | Traes_2AL_A6DF4B935-F | GGACAAGCTGGCAGACAAGC |
| **72** | Traes_2AL_A6DF4B935-R | CTCGGCCGTCTGGTTCTCAT |
| **73** | Traes_2BL_44AF6C8FD-F | AGCTCAACGCCGACCTCTTC |
| **74** | Traes_2BL_44AF6C8FD-R | GCTGCTGCACCTTGGGAATC |
| **75** | Traes_2BS_FF5A68083-F | ACACAGAGAACGCCGAGGAG |
| **76** | Traes_2BS_FF5A68083-R | CATGGCCGAGATGACAGGGT |
| **77** | Novel07753-F | GCATCCTCCCGAAGAAGCCT |
| **78** | Novel07753-R | TCGACCGGCGATTCACTTCA |
| **79** | Novel12259-F | CTGACGAGGTGACCATACTGCT |
| **80** | Novel12259-R | GACCTCCACAAGCTTTGCGT |
| **81** | Traes_1AS_36AF74187-F | GCCACCGATATCCCGGACTT |
| **82** | Traes_1AS_36AF74187-R | TGAGCTCAGTTTGGCGCTCT |
| **83** | TaActin-F | GAACCTCCACTGAGAACAACATTACC |
| **84** | TaActin-R | GTTCCAATCTATGAGGGATACACGC |
