# Supplementary material for: Transcriptome and physiological analyses for revealing genes involved in wheat response to endoplasmic reticulum stress
Source: BMC Plant Biol. 2019 May 9;19:193. doi: 10.1186/s12870-019-1798-7 (PMC6509841; doi:10.1186/s12870-019-1798-7)
Supplement: Supplementary file 6 — Table S3. Statistics of genes in different expression level intervals. (DOCX 13 kb) [file 12870_2019_1798_MOESM6_ESM.docx]

| **Table S3** Statistics of genes in different expression level intervals | | | |
| --- | --- | --- | --- |
| **FPKM Interval** | **C (%)** | **D (%)** | **T (%)** |
| **0~1** | 77895 (59.67) | 75248 (57.64) | 76459 (58.57) |
| **1~3** | 19242 (14.74) | 18562 (14.22) | 18443 (14.13) |
| **3~15** | 25199 (19.30) | 26741 (20.49) | 25742 (19.72) |
| **15~60** | 6646 (5.09) | 8184 (6.27) | 8028 (6.15) |
| **>60** | 1560 (1.20) | 1806 (1.38) | 1870 (1.43) |
| Notes: The data represent the average value of three biological replicates. C, control; D, DTT; T, DTT+TUDCA. | | | |
