# Supplementary material for: Transcriptome and physiological analyses for revealing genes involved in wheat response to endoplasmic reticulum stress
Source: BMC Plant Biol. 2019 May 9;19:193. doi: 10.1186/s12870-019-1798-7 (PMC6509841; doi:10.1186/s12870-019-1798-7)
Supplement: Supplementary file 9 — Table S5. DEGs relevant to the “protein processing in endoplasmic reticulum” pathway under group “D vs. C”. (DOCX 17 kb) [file 12870_2019_1798_MOESM9_ESM.docx]

| **Table S5** DEGs relevant to the “protein processing in endoplasmic reticulum” pathway under group “D vs. C” | | | | |
| --- | --- | --- | --- | --- |
| **Name** | **GeneID** | **log2FC** | ***P*-value** | **Annotation** |
| **Bip** | Traes_2BL_44AF6C8FD | 15.46 | 9.84E-04 | heat shock 70kDa protein 5 |
|  | Traes_2BS_FF5A68083 | 4.89 | 9.86E-03 | heat shock 70kDa protein 5 |
|  | Traes_6AS_236AF32FD | 1.32 | 8.39E-03 | heat shock 70kDa protein 5 |
| **GRP94** | Traes_5BS_AB86BB5DE | -1.00 | 9.93E-07 | heat shock protein 90kDa beta |
| **CNX** | Traes_6AS_9E2248EE8 | 1.21 | 5.88E-07 | calnexin |
| **PDIs** | Traes_4AL_F23B2CEFB | 1.88 | 2.38E-22 | protein disulfide-isomerase A1 [EC:5.3.4.1] |
|  | Traes_4BS_42361CB8D | 1.75 | 3.38E-18 | protein disulfide-isomerase A1 [EC:5.3.4.1] |
|  | Traes_4DS_26272902A | 1.16 | 3.46E-09 | protein disulfide-isomerase A1 [EC:5.3.4.1] |
|  | Traes_5AL_7AC09C7FF | 1.59 | 4.06E-05 | protein disulfide-isomerase A6 [EC:5.3.4.1] |
|  | Traes_5BL_D6603D993 | 1.36 | 2.59E-05 | protein disulfide-isomerase A6 [EC:5.3.4.1] |
|  | Traes_5DL_546404AFD | 1.16 | 3.03E-03 | protein disulfide-isomerase A6 [EC:5.3.4.1] |
| **Hsp40** | TRAES3BF024600080CFD_g | 1.04 | 1.21E-06 | DnaJ homolog subfamily C member 3 |
|  | Traes_3DL_330A01B46 | 1.22 | 2.68E-09 | DnaJ homolog subfamily C member 3 |
|  | Traes_3AL_75B505500 | 1.36 | 7.84E-11 | DnaJ homolog subfamily C member 3 |
| **Hsp70** | Traes_4BL_8C0E579F8 | 1.54 | 2.34E-03 | heat shock 70kDa protein 1/8 |
|  | Traes_4DL_7B7B7B0A8 | 5.27 | 2.07E-04 | heat shock 70kDa protein 1/8 |
|  | Traes_4AS_B978C93FA | 1.47 | 5.79E-13 | heat shock 70kDa protein 1/8 |
|  | Traes_1BL_EBAF41F1E | 1.20 | 1.89E-05 | heat shock 70kDa protein 1/8 |
|  | Traes_4AS_894CED0DA | 1.98 | 3.86E-04 | heat shock 70kDa protein 1/8 |
| **sHSF** | Traes_2BS_72656E777 | -2.24 | 7.47E-06 | HSP20 family protein |
|  | Traes_2DS_EBEF8CBBB | -1.03 | 3.01E-06 | HSP20 family protein |
|  | Traes_3AS_693536752 | 13.50 | 1.88E-03 | HSP20 family protein |
|  | Traes_4BL_EB9CF2010 | 2.13 | 2.91E-03 | HSP20 family protein |
|  | Traes_5BL_9FDF53F78 | -1.31 | 9.20E-04 | HSP20 family protein |
|  | Traes_6AS_27DE86D331 | -1.27 | 6.75E-10 | HSP20 family protein |
|  | Traes_6DL_4E9ADFC33 | 4.16 | 6.24E-04 | HSP20 family protein |
|  | Traes_7BS_A17E3A1E2 | 1.99 | 1.51E-04 | HSP20 family protein |
| **Sec61** | Traes_4AS_2D88ED3F8 | 6.08 | 1.61E-04 | protein transport protein SEC61 subunit alpha |
|  | Traes_4DL_BE50C5130 | 3.08 | 3.03E-04 | protein transport protein SEC61 subunit alpha |
|  | Traes_4BL_268EB5B53 | 4.13 | 2.14E-06 | protein transport protein SEC61 subunit alpha |
| **Glc Ⅰ** | Novel04851 | 1.77 | 3.60E-07 | mannosyl-oligosaccharide glucosidase [EC:3.2.1.106] |
| **NEF** | Traes_4BL_27330C1B8 | 1.37 | 7.99E-04 | hsp70-interacting protein |
|  | Traes_5BL_43C3BDED2 | 1.37 | 6.06E-04 | nucleotide exchange factor SIL1 |
|  | Traes_5DL_AA4815003 | 1.05 | 4.07E-04 | nucleotide exchange factor SIL1 |
|  | Traes_6AL_6518BC798 | 1.32 | 2.34E-04 | hypoxia up-regulated 1 |
|  | Traes_6BL_58BB13700 | 1.09 | 2.00E-03 | hypoxia up-regulated 1 |
|  | Traes_6DL_D9F5169D4 | 1.29 | 1.21E-08 | hypoxia up-regulated 1 |
| **SAR1** | Traes_7AL_FDC2E2F77 | 17.76 | 9.16E-07 | GTP-binding protein SAR1 [EC:3.6.5.-] |
|  | Traes_7DL_8D02A2581 | 5.28 | 1.58E-05 | GTP-binding protein SAR1 [EC:3.6.5.-] |
| **Bap31** | Traes_5DL_299466B1F | 1.34 | 8.55E-06 | B-cell receptor-associated protein 31 |
| **Otu1** | Traes_6DS_9ED841904 | -1.84 | 4.10E-14 | ubiquitin thioesterase OTU1 [EC:3.1.2.-] |
| **P97** | Traes_4AS_365CB9111 | -1.34 | 5.90E-05 | transitional endoplasmic reticulum ATPase |
| **RMA1** | Novel07430 | -1.58 | 5.48E-17 | E3 ubiquitin-protein ligase RNF5 [EC:6.3.2.19] |
|  | Novel07785 | -1.47 | 2.05E-14 | E3 ubiquitin-protein ligase RNF5 [EC:6.3.2.19] |
| **UbcH5** | Traes_6AS_E011BC5BB | -1.09 | 1.22E-05 | ubiquitin-conjugating enzyme E2 D/E [EC:6.3.2.19] |
|  | Traes_6BS_72F59E261 | 1.27 | 4.86E-03 | ubiquitin-conjugating enzyme E2 D/E [EC:6.3.2.19] |
| Notes: C, control; D, DTT. | | | | |
