# Supplementary material for: Transcriptome and physiological analyses for revealing genes involved in wheat response to endoplasmic reticulum stress
Source: BMC Plant Biol. 2019 May 9;19:193. doi: 10.1186/s12870-019-1798-7 (PMC6509841; doi:10.1186/s12870-019-1798-7)
Supplement: Supplementary file 10 — Table S6. DEGs relevant to the “plant hormone signal transduction” pathway under group “D vs. C”. (DOCX 18 kb) [file 12870_2019_1798_MOESM10_ESM.docx]

| **Table S6** DEGs relevant to the “plant hormone signal transduction” pathway under group “D vs. C” | | | | |
| --- | --- | --- | --- | --- |
| **Name** | **geneID** | **log2FC** | ***P*-value** | **Annotation** |
| **AUX1** | Traes_1AL_38EEA508C | -1.59 | 1.08E-03 | auxin influx carrier (AUX1 LAX family) |
|  | Traes_4DL_BF0B81D38 | 2.74 | 2.64E-03 | auxin influx carrier (AUX1 LAX family) |
| **AUX/IAA** | Novel08494 | -3.38 | 3.75E-04 | auxin-responsive protein IAA |
|  | Traes_1AL_859346448 | -1.01 | 4.24E-03 | auxin-responsive protein IAA |
|  | Traes_5AS_B39049539 | -1.60 | 2.73E-04 | auxin-responsive protein IAA |
|  | Traes_5DS_66E5D2F6D | -1.47 | 1.96E-04 | auxin-responsive protein IAA |
| **SAUR** | Traes_2BL_5191CFAEC | -3.00 | 2.41E-03 | SAUR family protein |
|  | Traes_6AS_2CE740C42 | -3.50 | 7.39E-03 | SAUR family protein |
|  | TRAES3BF053500020CFD_g | -2.58 | 1.75E-04 | SAUR family protein |
|  | TRAES3BF053500030CFD_g | -2.41 | 7.83E-03 | SAUR family protein |
| **B-ARR** | Traes_3AL_9D2735F60 | -1.04 | 1.78E-05 | two-component response regulator ARR-B family |
|  | Traes_3DL_D82CA7827 | -1.35 | 2.38E-04 | two-component response regulator ARR-B family |
| **A-ARR** | Traes_2AL_A26170C43 | -3.32 | 5.84E-24 | two-component response regulator ARR-A family |
|  | Traes_2BL_45DDBB56C | -3.26 | 4.71E-03 | two-component response regulator ARR-A family |
|  | Traes_2BL_BD5F3380A | -3.76 | 4.53E-24 | two-component response regulator ARR-A family |
|  | Traes_3DL_9DF0E96E5 | -3.04 | 1.90E-09 | two-component response regulator ARR-A family |
|  | TRAES3BF042500030CFD_g | -3.01 | 5.83E-19 | two-component response regulator ARR-A family |
| **GID1** | Traes_1BL_7517181F2 | 1.42 | 1.87E-03 | gibberellin receptor GID1 [EC:3.-.-.-] |
| **PYR/PYL** | Traes_2BS_6428AA6CC | -2.82 | 1.60E-28 | abscisic acid receptor PYR/PYL family |
|  | Traes_2DS_9A99C3160 | -3.21 | 3.75E-38 | abscisic acid receptor PYR/PYL family |
|  | Traes_4AS_72BEF89AC | -4.66 | 2.18E-26 | abscisic acid receptor PYR/PYL family |
|  | Traes_4BL_E43C1BB11 | -4.43 | 1.03E-45 | abscisic acid receptor PYR/PYL family |
|  | Traes_4DL_3A1814A74 | -4.31 | 1.98E-32 | abscisic acid receptor PYR/PYL family |
| **PP2C** | Novel01791 | 8.35 | 2.41E-13 | protein phosphatase 2C [EC:3.1.3.16] |
|  | Traes_1AL_087978D78 | -1.29 | 4.90E-03 | protein phosphatase 2C [EC:3.1.3.16] |
|  | Traes_1AL_C87568D4D | 4.42 | 5.82E-16 | protein phosphatase 2C [EC:3.1.3.16] |
|  | Traes_1DL_19271CD0B | 2.47 | 2.07E-10 | protein phosphatase 2C [EC:3.1.3.16] |
|  | Traes_2AS_048E13951 | 16.60 | 4.35E-16 | protein phosphatase 2C [EC:3.1.3.16] |
|  | Traes_2BS_C48CB635C | 16.18 | 2.92E-12 | protein phosphatase 2C [EC:3.1.3.16] |
|  | Traes_2DS_629ABEF42 | 9.86 | 1.07E-14 | protein phosphatase 2C [EC:3.1.3.16] |
|  | Traes_2DS_EB5F25337 | 9.09 | 5.90E-12 | protein phosphatase 2C [EC:3.1.3.16] |
|  | Traes_3AL_37DF311BB | 2.23 | 2.25E-14 | protein phosphatase 2C [EC:3.1.3.16] |
|  | Traes_3AL_67BEA8DDA | 4.52 | 4.83E-17 | protein phosphatase 2C [EC:3.1.3.16] |
|  | Traes_3AL_E58742B88 | 16.19 | 3.56E-10 | protein phosphatase 2C [EC:3.1.3.16] |
|  | Traes_3DL_5BFB28004 | 3.17 | 9.90E-07 | protein phosphatase 2C [EC:3.1.3.16] |
|  | Traes_3DL_66B0B40CD | 2.96 | 3.70E-23 | protein phosphatase 2C [EC:3.1.3.16] |
|  | Traes_3DL_71B7E80D2 | 15.46 | 9.76E-08 | protein phosphatase 2C [EC:3.1.3.16] |
|  | Traes_3DL_8C6D663C5 | 5.01 | 1.78E-17 | protein phosphatase 2C [EC:3.1.3.16] |
|  | Traes_4AS_CDC673BD9 | 3.39 | 1.12E-08 | protein phosphatase 2C [EC:3.1.3.16] |
|  | Traes_4BL_3403452C0 | 6.41 | 3.11E-12 | protein phosphatase 2C [EC:3.1.3.16] |
|  | Traes_4DL_EE9166AAD | 5.72 | 1.12E-11 | protein phosphatase 2C [EC:3.1.3.16] |
|  | Traes_5BL_1A9D2AC10 | 9.49 | 4.84E-18 | protein phosphatase 2C [EC:3.1.3.16] |
|  | Traes_5DL_C906A9208 | 16.84 | 1.29E-15 | protein phosphatase 2C [EC:3.1.3.16] |
|  | TRAES3BF017900010CFD_g | 2.34 | 3.68E-05 | protein phosphatase 2C [EC:3.1.3.16] |
|  | TRAES3BF018100030CFD_g | 2.16 | 3.32E-07 | protein phosphatase 2C [EC:3.1.3.16] |
|  | TRAES3BF051200120CFD_g | 17.10 | 2.83E-21 | protein phosphatase 2C [EC:3.1.3.16] |
|  | TRAES3BF069100050CFD_g | 4.58 | 2.06E-16 | protein phosphatase 2C [EC:3.1.3.16] |
|  | TRAES3BF111700080CFD_g | 3.87 | 5.34E-22 | protein phosphatase 2C [EC:3.1.3.16] |
| **SnRK2** | Traes_1AL_5FFFBAB47 | 3.17 | 1.63E-03 | serine/threonine-protein kinase SRK2 [EC:2.7.11.1] |
|  | Traes_1AL_E1701918C | 5.94 | 1.22E-07 | serine/threonine-protein kinase SRK2 [EC:2.7.11.1] |
|  | Traes_1BL_AC5FE4599 | 3.31 | 2.85E-03 | serine/threonine-protein kinase SRK2 [EC:2.7.11.1] |
|  | Traes_1BL_D20749C1D | 4.07 | 1.64E-07 | serine/threonine-protein kinase SRK2 [EC:2.7.11.1] |
|  | Traes_1DL_A332C35A8 | 2.67 | 1.56E-04 | serine/threonine-protein kinase SRK2 [EC:2.7.11.1] |
|  | Traes_1DL_E331E6071 | 5.73 | 9.02E-08 | serine/threonine-protein kinase SRK2 [EC:2.7.11.1] |
|  | Traes_2AL_2FF604DA9 | 3.50 | 1.01E-13 | serine/threonine-protein kinase SRK2 [EC:2.7.11.1] |
|  | Traes_2AL_C37FA7FDC | 1.10 | 6.04E-06 | serine/threonine-protein kinase SRK2 [EC:2.7.11.1] |
|  | Traes_2AS_64E0EF151 | -1.39 | 1.09E-05 | serine/threonine-protein kinase SRK2 [EC:2.7.11.1] |
|  | Traes_2BL_616F08780 | 6.76 | 4.38E-11 | serine/threonine-protein kinase SRK2 [EC:2.7.11.1] |
|  | Traes_2BL_B805C339B | 1.44 | 3.50E-11 | serine/threonine-protein kinase SRK2 [EC:2.7.11.1] |
|  | Traes_2DL_355E90C65 | 1.36 | 8.74E-06 | serine/threonine-protein kinase SRK2 [EC:2.7.11.1] |
|  | Traes_2DL_6CD064E13 | 2.41 | 9.86E-20 | serine/threonine-protein kinase SRK2 [EC:2.7.11.1] |
|  | Traes_4BS_0A234D9E6 | 1.11 | 1.37E-04 | serine/threonine-protein kinase SRK2 [EC:2.7.11.1] |
| **ABF** | Traes_3AL_17988A22C | 3.87 | 2.56E-07 | ABA responsive element binding factor |
|  | Traes_5AL_79E6A58E6 | 1.28 | 1.52E-03 | ABA responsive element binding factor |
|  | Traes_6AL_E8CD2C02B | 2.54 | 1.84E-14 | ABA responsive element binding factor |
|  | Traes_6BL_A530B5920 | 2.24 | 2.88E-12 | ABA responsive element binding factor |
|  | Traes_6DL_E87DBFB1C | 1.61 | 9.80E-12 | ABA responsive element binding factor |
|  | Traes_6DL_F7015CE89 | 1.97 | 1.27E-22 | ABA responsive element binding factor |
|  | Traes_7BS_280163F24 | 3.11 | 8.05E-06 | ABA responsive element binding factor |
|  | Traes_7DS_C6A3C10A6 | 1.99 | 1.98E-07 | ABA responsive element binding factor |
|  | TRAES3BF019000220CFD_g | 2.42 | 9.80E-17 | ABA responsive element binding factor |
| **EIN2** | Novel05921 | 1.37 | 2.41E-09 | ethylene-insensitive protein 2 |
|  | Traes_4DS_2706FACC1 | -1.37 | 1.06E-12 | ethylene-insensitive protein 2 |
| **EIN3** | Traes_7AS_34D476D5D1 | -1.22 | 7.92E-08 | ethylene-insensitive protein 3 |
|  | Traes_7BS_733347AC0 | -1.16 | 4.78E-07 | ethylene-insensitive protein 3 |
| **EBF1/2** | Traes_6AS_5A19432A11 | -1.14 | 4.94E-09 | EIN3-binding F-box protein |
| **BRI1** | Novel01018 | 14.93 | 2.98E-12 | protein brassinosteroid insensitive 1 [EC:2.7.10.1 2.7.11.1] |
|  | Novel03728 | -1.13 | 2.50E-03 | protein brassinosteroid insensitive 1 [EC:2.7.10.1 2.7.11.1] |
|  | Traes_4BL_5CA0D5F67 | 13.55 | 6.48E-05 | protein brassinosteroid insensitive 1 [EC:2.7.10.1 2.7.11.1] |
| **JAR1** | Traes_1BL_ACAA87960 | -1.21 | 5.05E-07 | jasmonic acid-amino synthetase |
|  | TRAES3BF077300030CFD_g | -1.14 | 7.86E-09 | jasmonic acid-amino synthetase |
| **NPR1** | Traes_4BS_28D2F8459 | -1.69 | 1.69E-08 | regulatory protein NPR1 |
|  | Traes_4DS_73E62EF87 | -1.04 | 6.72E-04 | regulatory protein NPR1 |
|  | TRAES3BF089000240CFD_g | -2.23 | 2.04E-03 | regulatory protein NPR1 |
|  | TRAES3BF104900090CFD_g | -1.30 | 2.71E-05 | regulatory protein NPR1 |
| **TGA** | Traes_1AL_3B659AABA | -3.74 | 4.73E-04 | transcription factor TGA |
|  | Traes_2AL_BD0C7A9A5 | 1.36 | 2.27E-06 | transcription factor TGA |
|  | Traes_2BL_9528AAD7C | 1.68 | 3.02E-10 | transcription factor TGA |
| **PR-1** | Traes_5BL_B137C7E4F | -4.49 | 9.34E-03 | pathogenesis-related protein 1 |
|  | Traes_7AS_406885D49 | -1.91 | 1.28E-07 | pathogenesis-related protein 1 |
|  | Traes_7AS_7E99B3482 | -2.40 | 3.52E-21 | pathogenesis-related protein 1 |
|  | Traes_7AS_FEE6CC75E1 | -2.24 | 2.70E-08 | pathogenesis-related protein 1 |
|  | Traes_7BS_8D9D397291 | -1.62 | 2.76E-05 | pathogenesis-related protein 1 |
|  | Traes_7BS_CB04276DC | -1.89 | 8.85E-07 | pathogenesis-related protein 1 |
|  | Traes_7DS_1797D4951 | -1.69 | 2.14E-11 | pathogenesis-related protein 1 |
|  | Traes_7DS_5969C1C34 | -1.74 | 7.36E-06 | pathogenesis-related protein 1 |
|  | Traes_7DS_A6D32CAA9 | -1.98 | 8.31E-14 | pathogenesis-related protein 1 |
|  | Traes_7DS_ABBDA3DBB | -1.43 | 1.10E-06 | pathogenesis-related protein 1 |
| Notes: C, control; D, DTT. | | | | |
