# Supplementary material for: Transcriptome and physiological analyses for revealing genes involved in wheat response to endoplasmic reticulum stress
Source: BMC Plant Biol. 2019 May 9;19:193. doi: 10.1186/s12870-019-1798-7 (PMC6509841; doi:10.1186/s12870-019-1798-7)
Supplement: Supplementary file 14 — Table S9. The FPKM of DEGs related to the “protein processing in endoplasmic reticulum” pathway. (DOCX 17 kb) [file 12870_2019_1798_MOESM14_ESM.docx]

| **Table S9** The FPKM of DEGs related to the “protein processing in endoplasmic reticulum” pathway | | | | |
| --- | --- | --- | --- | --- |
| **Name** | **Gene ID** | **FPKM** | | |
|  |  | **C** | **D** | **T** |
| **Bip** | Traes_1BL_EFDDA2877 | 0.001 | 0.11 | 0.62 |
|  | Traes_2AL_A6DF4B935 | 0.02 | 1.72 | 7.45 |
|  | Traes_2BL_44AF6C8FD | 0.001 | 3.66 | 10.95 |
|  | Traes_2BS_FF5A68083 | 0.01 | 0.46 | 1.66 |
|  | Traes_6AS_236AF32FD | 11.16 | 33.07 | 74.30 |
|  | Traes_7DL_988A0B630 | 0.001 | 0.28 | 0.56 |
| **GRP94** | Traes_5BS_AB86BB5DE | 9.86 | 5.77 | 8.65 |
| **CNX** | Traes_6AS_9E2248EE8 | 4.26 | 11.66 | 18.80 |
| **PDI** | Novel09086 | 3.02 | 5.81 | 7.73 |
|  | Traes_4AL_F23B2CEFB | 15.78 | 68.45 | 103.64 |
|  | Traes_4BS_42361CB8D | 15.40 | 60.33 | 86.49 |
|  | Traes_4DS_26272902A | 16.44 | 43.16 | 75.41 |
|  | Traes_5AL_7AC09C7FF | 4.66 | 16.53 | 41.94 |
|  | Traes_5BL_D6603D993 | 5.02 | 15.23 | 39.93 |
|  | Traes_5DL_546404AFD | 6.59 | 17.34 | 42.76 |
|  | Traes_6AS_5896DC565 | 4.24 | 3.42 | 2.34 |
| **Hsp40** | Traes_3AL_75B505500 | 5.61 | 16.88 | 30.65 |
|  | Traes_3DL_330A01B46 | 11.69 | 32.06 | 51.93 |
|  | TRAES3BF024600080CFD_g | 9.20 | 22.15 | 35.94 |
| **Hsp70** | Traes_1BL_627D564A9 | 1.94 | 3.35 | 11.81 |
|  | Traes_1BL_EBAF41F1E | 1.98 | 5.39 | 30.35 |
|  | Traes_4AS_894CED0DA | 0.46 | 2.11 | 0.98 |
|  | Traes_4AS_B978C93FA | 4.70 | 15.48 | 41.04 |
|  | Traes_4BL_4D370C9FA1 | 0.02 | 0.15 | 0.61 |
|  | Traes_4BL_8C0E579F8 | 1.04 | 3.55 | 2.58 |
|  | Traes_4BL_E73DF7876 | 3.88 | 5.09 | 13.94 |
|  | Traes_4DL_1604A9F06 | 15.67 | 16.96 | 35.53 |
|  | Traes_4DL_7B7B7B0A8 | 0.02 | 1.01 | 0.32 |
| **sHSF** | Traes_2BS_72656E777 | 4.70 | 1.18 | 1.34 |
|  | Traes_2DS_EBEF8CBBB | 18.02 | 10.40 | 10.29 |
|  | Traes_3AS_693536752 | 0.001 | 0.71 | 0.22 |
|  | Traes_4BL_EB9CF2010 | 0.17 | 0.87 | 7.00 |
|  | Traes_5BL_9FDF53F78 | 1.88 | 0.89 | 4.24 |
|  | Traes_6AS_27DE86D331 | 54.34 | 26.38 | 27.96 |
|  | Traes_6DL_4E9ADFC33 | 0.10 | 2.08 | 4.05 |
|  | Traes_7BS_A17E3A1E2 | 0.48 | 2.15 | 3.62 |
| **Sec13/31** | TRAES3BF036500010CFD_g | 3.23 | 5.20 | 7.45 |
| **Sec23/24** | Novel07145 | 4.82 | 4.18 | 2.60 |
|  | Traes_2DS_E77C37438 | 6.79 | 4.79 | 2.79 |
|  | Traes_4BL_FD31350CB | 4.18 | 3.40 | 2.28 |
| **SEC61** | Traes_3DL_559FF19FE | 6.48 | 13.03 | 21.08 |
|  | Traes_4AS_2D88ED3F8 | 0.04 | 3.03 | 10.67 |
|  | Traes_4BL_268EB5B53 | 0.61 | 12.62 | 23.74 |
|  | Traes_4DL_BE50C5130 | 1.57 | 15.80 | 31.87 |
|  | Traes_5AL_B46AC4031 | 14.06 | 9.53 | 7.22 |
|  | Traes_5BL_2CE7AAFD8 | 24.45 | 16.76 | 10.18 |
| **SAR1** | Traes_3DS_FB1CE2622 | 9.94 | 18.85 | 24.44 |
|  | Traes_7AL_FDC2E2F77 | 0.001 | 10.05 | 32.75 |
|  | Traes_7DL_8D02A2581 | 0.03 | 1.50 | 5.63 |
| **RMA1** | Novel07430 | 141.45 | 55.57 | 101.28 |
|  | Novel07785 | 88.15 | 37.61 | 79.07 |
| **UbcH5** | Traes_6AS_E011BC5BB | 11.73 | 6.45 | 4.36 |
|  | Traes_6BS_72F59E261 | 4.16 | 11.79 | 4.51 |
| **Bap31** | Traes_5DL_299466B1F | 1.75 | 5.19 | 3.38 |
| **Glc Ⅰ** | Novel04851 | 0.72 | 2.93 | 2.37 |
| **NEF** | Traes_4BL_27330C1B8 | 0.45 | 1.37 | 1.46 |
|  | Traes_5BL_43C3BDED2 | 1.27 | 3.90 | 9.66 |
|  | Traes_5DL_AA4815003 | 1.57 | 3.83 | 5.21 |
|  | Traes_6AL_6518BC798 | 6.23 | 18.31 | 31.28 |
|  | Traes_6BL_58BB13700 | 6.51 | 16.41 | 29.08 |
|  | Traes_6DL_D9F5169D4 | 4.47 | 12.99 | 24.17 |
| **OSTs** | Traes_2DL_E9D667EFD | 7.79 | 4.86 | 3.34 |
| **OTU1** | Traes_6AS_0FDBBF9E9 | 8.41 | 5.30 | 4.67 |
|  | Traes_6BS_6D541D2C1 | 5.03 | 3.08 | 2.75 |
|  | Traes_6DS_9ED841904 | 11.67 | 3.82 | 4.49 |
| **P97** | Traes_4AS_365CB9111 | 11.70 | 5.44 | 4.15 |
| Notes: C, control; D, DTT; T, DTT+TUDCA. | | | | |
