# Supplementary material for: Transcriptome and physiological analyses for revealing genes involved in wheat response to endoplasmic reticulum stress
Source: BMC Plant Biol. 2019 May 9;19:193. doi: 10.1186/s12870-019-1798-7 (PMC6509841; doi:10.1186/s12870-019-1798-7)
Supplement: Supplementary file 17 — Table S10. The FPKM and annotation of DEGs that may be served as potential candidate genes. (DOCX 14 kb) [file 12870_2019_1798_MOESM17_ESM.docx]

| **Table S10** The FPKM and annotation of DEGs that may be served as potential candidate genes | | | | |
| --- | --- | --- | --- | --- |
| **Gene ID** | **Annotation** | **FPKM** | | |
|  |  | **C** | **D** | **T** |
| **Traes_2BS_DABEABDDC** | mlo protein | 0.13 | 0.58 | 1.65 |
| **Traes_1AL_68FA687CC** | trehalose 6-phosphate phosphatase | 0.33 | 1.28 | 0.53 |
| **TRAES3BF274400020CFD_g** | delta-1-pyrroline-5-carboxylate synthetase | 0.001 | 4.64 | 1.36 |
| **Novel15990** | Protein SRG1 [Aegilops tauschii] | 6.65 | 3.42 | 7.56 |
| **Traes_2DL_F2D3DED15** | 2-oxoglutarate (2OG) and Fe(II)-dependent oxygenase superfamily protein | 2.55 | 7.03 | 15.11 |
| **Traes_2BS_C02C5A463** | Pectin lyase-like superfamily protein | 0.001 | 0.73 | 0.001 |
| **Novel16561** | Dehydrin DHN3 [Triticum urartu] | 0.07 | 43.51 | 9.39 |
| **Novel09719** | Cold shock protein CS66 [Triticum urartu] | 0.02 | 12.76 | 3.35 |
| **Traes_7BL_4253AC95E** | novel plant SNARE | 1.42 | 4.37 | 1.95 |
| **Traes_4BS_E79CB87B7** | thiamin C /phosphomethylpyrimidine synthase [EC:4.1.99.17] | 14.91 | 28.53 | 13.65 |
| Notes: C, control; D, DTT; T, DTT+TUDCA. | | | | |
